# Assessment of performance and challenges of small and micro enterprises: Fireweyni town, Ethiopia

**DOI:** 10.1371/journal.pone.0320681

**Published:** 2025-04-08

**Authors:** Tuem Gebre Abraha, Haftom Teshale Gebre

**Affiliations:** 1 Department of Statistics, College of natural and computational sciences, Adigrat University, Adigrat, Ethiopia; 2 Department of Geography and Environmental Science, College of Social Science and Humanities, Adigrat University, Adigrat, Ethiopia; King Khalid University, SAUDI ARABIA

## Abstract

Micro and small enterprises are crucial drivers of economic growth, job creation, and poverty reduction in developing countries. Recognizing this, Ethiopia has implemented measures to enhance the operation of micro and small enterprises. However, many micro and small enterprises in the country face significant challenges and exhibit deteriorating performance, with limited progression from one enterprise level to the next. This study aims to assess the performance and challenges of micro and small enterprises in Fireweyni town. Employing a mixed-methods approach, the study integrates both qualitative and quantitative research. Primary data were collected using a structured questionnaire. A probability sampling technique, combining convenience and stratified random sampling, was applied to a sample of 373 micro and small enterprises in Fireweyni town. The study is descriptive and cross-sectional, focusing on the performance of MSEs and the obstacles they encounter. Data collection included responses from 5483 micro and small enterprise operators, distributed across service (853), manufacturing (341), urban agriculture (1073), construction (155), and trade (3061) sectors. The data were analyzed using descriptive and inferential statistical methods, with the aid of the Statistical Package for Social Sciences (SPSS) version 20 and STATA version 16. Results of the study revealed that, every variable (factor) incorporated in this study was statistically significant, impacting the performance of SMEs and businesses within the study area. The Pearson correlation coefficient test results showed that all independent variables have a significant relationship with MSE business performance. The study’s conclusions indicate that financial control, marketing strategy, managerial expertise, government regulations, and business information services significantly affect the performance of small and micro businesses in Fireweyni town.

## Background of the study

Small and micro-sized enterprises (SMEs) are critical drivers of economic growth, job creation, and poverty reduction in developing countries due to their contributions to GDP and employment [1,2]. The classification of SMEs varies globally, typically based on the number of employees and annual revenue [3,4]. India introduced the term MSME (Micro, Small, and Medium Enterprises) to acknowledge the role of micro-sized businesses in economic development. This classification has since been adopted by several other countries [4,5].

In the Maldives, SMEs are classified based on the number of employees, total annual revenue, and net profit, as outlined in the Small and Medium Enterprise. This classification helps businesses identify appropriate financing options, government support, and business structuring strategies [5].

In Ethiopia, SMEs are essential for economic development, providing significant employment opportunities with low capital requirements [6]. Despite governmental support, many SMEs face substantial challenges, including financial constraints, inadequate infrastructure, and limited market access [7].

In this study, activities are made to answer some of the questions that are remained unanswered regarding the Assessment of Performance and Challenges of Small and Micro Enterprises: Fireweyni Town, Ethiopia. Those: - What looks like the connections between MSE performance and financial control systems, management experience, business information services, and government regulation, Is there relationship between various factors and the performance of MSE operators in Fireweyni town, and what are the determinant factors of Performance and Challenges of Small and Micro Enterprises: Fireweyni Town, Ethiopia.

This study investigates the performance and challenges of micro and small enterprises in Fireweyni town using a mixed-methods approach. Primary data were collected through structured questionnaires, while secondary data were sourced from books, articles, and previous studies. A sample of 373 SMEs was selected through stratified and simple random sampling techniques. The study employs descriptive and cross-sectional analysis to explore SME performance and obstacles. Data analysis was conducted using SPSS version 20, applying both descriptive and inferential statistical tools. Results indicate that SMEs in Fireweyni town are significantly hindered by access to finance, infrastructure, and market linkages, affecting their growth and performance.

## Research methodology

### Verbal consent script for ethics statement

We have gotten Ethical clearance letter from Department of Statistics, College of Natural and computational Sciences, Adigrat University Research Review Committee. Moreover, we deal verbal consent and get verbal agreement from the participants as:

### Description of the study’s purpose for the research participant

We aim to gather information that will help us understand the current state of small and micro enterprises in Fireweyni Town, the challenges they face, and potential solutions to improve their performance.

#### Procedures.

If you agree to participate, you will be asked to answer several questions about your business. This interview will take approximately 30 minutes. Your responses will be recorded for accuracy.

#### Voluntary participation.

Your participation in this study is entirely voluntary. You may choose not to answer any questions that make you uncomfortable, and you can stop the interview at any time without any penalty.

#### Consent and confidentiality.

All the information you provide will be kept confidential. Your identity will not be disclosed in any reports or publications resulting from this study. The data will be anonymized to protect your privacy.

Do you agree to participate in this study?

(Wait for the participant to respond.)

#### Respond of respondents (participants) for the consent.

“Based on the request, you have asked us to participate in the study you have submitted to do this. We are agreeing and willing to participate in the study because of the purpose of the study and its benefits to our society and organization.”

### Recruitment period of the study

This study was conducted on the scheduled time from January 20, 2024 to May 30, 2024.

### Description of study area

#### Location.

Seasia tsaeda emba woreda is found in the eastern zone of Tigray region. It is located between 13^0^55’00”N and 14^0^23’30”N latitude and 39^0^28’30”E and 39^0^53’00”E longitude [8]. This woreda is located 78 km away from Mekelle town and it is 37 km far from Adigrat town via northern zone. Relatively, Seasia tsaeda emba woreda lies north of Gulemekeda woreda; east of Atsbi-wenberta woreda and Afar region; south of Klteawlaelo, Hawuzian and Ganta-afeshum woredas [9].

### Research design

This study employs an explanatory research design, incorporating both qualitative and quantitative approaches to assess the performance of micro and small enterprises (MSEs). The quantitative aspects of the study include age, total number of households, and yearly profit, while the qualitative aspects cover gender, sector types, job types, education level, work sheds, and types of enterprises.

The mixed-methods approach is suitable for verifying observations related to the research questions. Qualitative data were gathered through personal interviews and observations of business owners, while quantitative data were collected using structured questionnaires. The questionnaire was designed to be straightforward to encourage meaningful participation from respondents.

### Data collection

The study utilized both primary and secondary data sources:

- **Primary Data:** Collected directly from MSE owners using a well-designed, structured questionnaire S1 File.
- **Secondary Data:** list of MSE sectors, number of participants with their corresponding MSE sectors were obtained from un-published recorded files from central statistics agency plan and finance office of Tigray region, 2024.

This combination of data sources ensured a comprehensive understanding of the factors influencing MSE performance.

### Data type and source

To achieve the objective of this study, both primary and secondary data have been utilized. Primary data was collected through questionnaires and other techniques. These questionnaires were provided with translations in both English and the local language to ensure comprehensibility among respondents. Additionally, in-depth interviews were conducted with purposefully selected key informants to gather detailed insights. While secondary data was sourced from recorded files regarding profiles of the respondents.

### Study population/target population

The population of the study consists of 5,483 members of MSEs in Fireweyni town, categorized into urban agriculture, trade, service, manufacturing, and construction sectors obtained from central statistics agency plan and finance office of Tigray region, 2024. Because there was finite population, the sample size determined using the formula developed by Taro Yamane’s [10]. According to Yamane’s formula, the sample size n is determined using the equation:

$n=N/[1+N{\left(e\right)}^{2}]$ , n is sample size, N is population size, and e is margin of error.

where N represents the total population, and e is the sampling error. For this study, a 5% sampling error and a 95% confidence level were used. Applying the formula, the sample size calculation is as follows:


$$n=N/[1+N{\left(e\right)}^{2}]n=5483/[1+5483{\left(0.05\right)}^{2}]=>372.80=>373$$

Therefore, the required sample size for the study is 373 Table 1.

**Table 1:** Sample size of each Stratum.

Stratum based on category	Population(N)	Proportion population stratum (PI)	Sample size from each stratum(${\text{n}}_{h}=\frac{{\text{N}}_{h}}{N}*n$)
Urban agriculture	1073	19.5%	73
Trade	3061	55.8%	208
Service	853	15.5%	58
Manufacturing	341	6.2%	23
Construction	155	2.8%	11
Total	5483	100%	373

### Sampling technique and sampling size

Following this from the total of 5483 MSE members in the frame samples of 373 are randomly selected with convenience. The total members of MSEs of in Fireweyni were collected from central statistics agency plan and finance office of Tigray region, 2024.

Table 1 is formulated by the stratified formula; ${\text{n}}_{h}=\frac{{\text{N}}_{h}}{N}*n$ [10].

Where, n_h_ = sample size of h^th^ strata, N_h_ = population size of h^th^ strata, n = total sample size, and N = total population size.

### Data analysis

The processed data was analyzed to identify patterns and relationships between and among data groups using both descriptive and inferential statistical analysis. The Statistical Package for Social Science (SPSS) version 20 and STATA version 16 were employed to analyze the primary data. Descriptive statistics, such as mean, standard deviation, and bar graphs, were used to summarize the data. Additionally, inferential statistics, including correlation and regression analyses, were applied to explore the relationships and draw conclusions from the data.

### Descriptive analysis

Descriptive analysis was employed to condense the data into a summary format through tabulation and measures of central tendency, specifically mean and standard deviation. Tables were used to describe the general characteristics of the enterprises. Descriptive statistics were essential for comparing different factors. Additionally, interview responses were analyzed using descriptive narrations, facilitated by a concurrent triangulation strategy. This approach ensured a comprehensive understanding by combining quantitative data with qualitative insights

### Inferential analysis

In our study the dependent variable is performance which is measured in the continuous manner and follows normal distribution. Hence, if the data has known form of distribution the appropriate test is parametric test with appropriate distribution. So, we have conducted parametric test for this study.

According to [11], inferential statistics enable the analysis of relationships between two or more variables and help determine how several independent variables may explain the variance in a dependent variable. The following inferential statistical methods were used in this study:

1. **Correlation Analysis:** To examine the strength and direction of the relationship between variables.
2. **Regression Analysis:** To identify the extent to which independent variables predict the dependent variable.
3. **ANOVA (Analysis of Variance):** To compare means among different groups and determine if any significant differences exist.

### The Pearson product moment correlation coefficient

According to [12], inferences play a crucial role in management research as conclusions are typically based on results. Generalizations for the population are made based on sample data. They highlight that the Pearson Product Moment Correlation Coefficient is a widely used statistical method for measuring the linear relationship between two continuous variables. To determine if a statistically significant relationship exists between financial factors, marketing skills and strategies, government policy and regulation, business information services, management factors, and a firm’s performance, the Product Moment Correlation Coefficient was employed.

[13] explains that the correlation coefficient can range from -1 to + 1. A value of -1 indicates a perfect negative correlation, + 1 indicates a perfect positive correlation, and 0 indicates no relationship. The standard [13], of the correlation coefficient values is as follows Table 2:

**Table 2:** Standard Unit for Correlation coefficient.

Coefficient Range	Interpretation
±0.91 to ± 1.00	Very Strong
±0.71 to ± 0.90	High
±0.41 to ± 0.70	Moderate
±0.21 to ± 0.40	Small but definite relationship
±0.01 to ± 0.20	Slight, almost negligible

This method was used to ascertain the relationships between the mentioned variables and the performance of firms.

### Linear regression analysis

Linear regression is a method used to estimate or predict the value of a dependent variable based on the values of one or more independent variables. While correlations examine the association between variables, the primary purpose of regression is prediction [14]. In this study, multiple regression analysis was employed. This method considers the inter-correlations among all involved variables and the correlations among the predictor scores [15]. Multiple regression analysis involves regressing more than one predictor jointly against the criterion variable to determine if the independent variables explain the variance in the dependent variable.

### Regression functions

The regression equation in this study constructed around two sets of variables: the dependent variable (performance) and the independent variables (financial factors, marketing skills and strategies, government policy and regulation, business information services, and management). The primary objective of using regression equations is to enhance the study’s ability to describe, understand, and predict the relationships among these variables.

### Regress performance on selected variables


$$\text{Yi}={\text{β}}_{0}+{\text{β}}_{1}{\text{X}}_{1}+{\text{β}}_{2}{\text{X}}_{2}+{\text{β}}_{3}{\text{X}}_{3}+{\text{β}}_{4}{\text{X}}_{4}+{\text{β}}_{5}{\text{X}}_{5}+\text{e}$$

Where:

Y is the response or dependent variable- performance

X1 = Financial, X2 = government policy and regulation, X3 = business information services, X4 = marketing skill and strategy, X5 = Management experience are the explanatory variables.

β0 is the intercept term- constant which would be equal to the mean if all slope coefficients are 0.

β1, β2, β3, β4, and β5, are the coefficients associated with each independent variable which measures the change in the mean value of Y, per unit change in their respective independent variables.

Accordingly, this statistical technique was used to explain the following relationships: Regress performance (as a dependent variable) on the selected linear combination of the independent variables using multiple regressions.

## Results and discussions

### Response rate

To evaluate the performance and challenges faced by MSEs, a total of 373 questionnaires were distributed to individuals, members, and SMEs currently operating in the case area, encompassing the construction, urban agriculture, trade, manufacturing, and service sectors. Of the distributed questionnaires, 336 were returned, resulting in a response rate of 90.08%.

#### Demographic characteristics of respondents.

This section begins with a frequency and percentage analysis of the demographic information collected from the respondents. Table 3 presents the attributes of the general respondents, including sex, age, marital status, educational level, and employment history.

**Table 3:** Demographic Characteristics of Respondents.

Variables	Frequency	Percent
**Gender**		
Male	229	68.2
Female	107	31.8
Total	336	100.0
Age category	Frequency	Percent
18-25 years	77	22.9
26-33 years	92	27.4
34-41 years	109	32.4
above 41	58	17.3
Total	336	100.0
**Education levels**	Frequency	Percent
Finished Ethiopian Secondary school 10 + 3	94	28.0
Earned a diploma from a recognized institution	54	16.1
BA degree	34	10.1
Education level below 10	154	45.8
Total	336	100.0
**Enterprise category**	Frequency	Percent
Construction	12	3.6
Manufacturing	18	5.4
Service	48	14.3
Urban agriculture	58	17.3
Trade	200	59.5
Total	336	100.0
**Age of the business**	Frequency	Percent
Less than 1 year	159	47.3
1–3 years	41	12.2
4–6 years	50	14.9
Above 6 years	86	25.6
Total	336	100.0

As Table 3 indicates, the gender distribution of the respondents indicates that 68.2% are male (229 respondents), while 31.8% are female (107 respondents). This description of data shows that the male population is more represented in this study, which could reflect the gender representation in this study within the SME sector in Fireweyni town. It is essential to consider this gender imbalance when analyzing the results, as it might influence the interpretation of findings related to gender-specific issues in business operations. The age distribution reveals a diverse range of respondents. Specifically, 22.9% (77 respondents) are between 18–25 years, 27.4% (92 respondents) are between 26–33 years, 32.4% (109 respondents) are between 34–41 years, and 17.3% (58 respondents) are above 41 years. The majority of respondents fall within the 26–41 age range of the data we collected. The relatively lower percentage of respondents above 41 years suggests fewer older individuals are engaged in SMEs study, possibly due to retirement or transitioning to fewer demanding roles. The educational background of respondents is varied. About 28.0% (94 respondents) have finished Ethiopian Secondary school 10 + 3, 16.1% (54 respondents) have earned a diploma from a recognized institution, 10.1% (34 respondents) hold a BA degree, and 45.8% (154 respondents) have an education level below 10. The data indicates a significant portion of respondents have lower educational attainment, with 45.8% having education levels below 10. This highlights the need for targeted educational programs and training to enhance the skills and competencies of SME operators. The 28.0% with secondary education and 16.1% with diplomas suggest a moderately educated workforce, while the 10.1% with BA degrees indicate a smaller group with higher education. The respondents are engaged in various enterprise categories. Specifically, 3.6% (12 respondents) are in construction, 5.4% (18 respondents) are in manufacturing, 14.3% (48 respondents) are in service, 17.3% (58 respondents) are in urban agriculture, and 59.5% (200 respondents) are in trade. Trade is the predominant category, accounting for 59.5% of respondents. This dominance indicates that trading activities of these respondents are the primary economic drivers in Fireweyni town’s SME sector. The presence of respondents in urban agriculture (17.3%), service (14.3%), manufacturing (5.4%), and construction (3.6%) showcases a diversified economic base, though these sectors are less represented compared to trade. The business age distribution is as follows: 47.3% (159 respondents) have businesses less than 1 year old, 12.2% (41 respondents) have been operating for 1–3 years, 14.9% (50 respondents) have been operating for 4–6 years, and 25.6% (86 respondents) have businesses older than 6 years. Nearly half of the businesses (47.3%) are less than a year old, indicating a high rate of new business ventures. This could suggest a dynamic entrepreneurial environment, though it also raises questions about the sustainability and longevity of these enterprises. The 25.6% of businesses older than six years reflect a degree of stability and established presence in the market.

According Table 4, the majority of respondents believe that government policies and regulations significantly impact the performance of SME businesses, as reflected by a high mean of 4.33 and a standard deviation of 1.258. Specifically, 73.5% of respondents strongly agree with this statement, while only 7.7% strongly disagree. This high level of agreement underscores the critical role that regulatory frameworks play in shaping the operational environment of SMEs. Recent studies corroborate these findings, emphasizing that supportive regulatory environments are essential for SME growth and sustainability [16]. The influence of government policies can range from ease of doing business to access to finance and market opportunities, making it a pivotal factor in SME performance. The mean score of 4.07 and a standard deviation of 1.534 indicate that respondents perceive taxation policy and regulation as major factors affecting business performance. Notably, 72.3% strongly agree with this sentiment, while 27.7% are in disagreement. The lack of neutral responses suggests a polarized view on taxation’s impact. This aligns with the literature, where high tax rates and complex tax compliance processes are often cited as significant challenges for SMEs [17]. Effective tax policies should balance revenue generation with the need to support business development, ensuring that tax burdens do not stifle SME growth. The variable concerning license processing to start a business has a mean of 4.30 and a standard deviation of 1.296, with 77.1% of respondents strongly agreeing that it affects business performance. This high agreement indicates that cumbersome licensing processes are perceived as significant barriers. The finding aligns with research showing that streamlining business registration and licensing processes can substantially improve SME productivity and growth (Smith et al., 2024). Simplified procedures reduce the time and cost associated with starting a business, enabling entrepreneurs to focus on business development rather than bureaucratic hurdles. The impact of government regulations on the liberalization of the economy has a mean score of 3.82 and a standard deviation of 1.758. Among respondents, 67.9% strongly agree that these regulations affect SME performance, while a notable 25% strongly disagree. This mixed response suggests that while many see economic liberalization as beneficial, a significant minority perceive it as problematic. This divergence could be due to the varying impacts of liberalization across different sectors and regions. Recent discussions have highlighted that while economic liberalization can create opportunities by opening up markets and encouraging competition, it may also pose challenges by exposing SMEs to increased competition and market volatility [18].

**Table 4:** Descriptive statistics on government regulation (means and standard deviations).

Item Variables		Cases in percent & Frequency					Mean	SD
		Strongly disagree	Disagree	Neutral	Agree	Strongly agree		
The government policies and regulations affect performance of SME businesses	F	26	21	27	15	247	4.33	1.258
	%	7.7	6.3	8.0	4.5	73.5		
Taxation policy and regulation affect business performance of SME	F	35	58	0	0	243	4.07	1.534
	%	10.4	17.3	0.0	0.0	72.3		
License processing to start the business affect business performance of SME	F	9	53	15	0	259	4.30	1.296
	%	2.7	15.8	4.5	0.0	77.1		
Government regulations on liberalization of the economy affect business performance of SME	F	84	13	11	0	228	3.82	1.758
	%	25.0	3.9	3.3	0.0	67.9		

According to Table 5, the availability of business information to respondents is highly rated, with a mean of 4.28 and a standard deviation of 1.271. A significant 74.4% of respondents strongly agree that business information is readily available, while only 2.7% strongly disagree. This suggests that the majority of SMEs feel well-informed about the business environment. Access to timely and relevant information is crucial for strategic decision-making and operational efficiency in SMEs, as emphasized by recent studies [19]. When assessing the relevance of available information, the mean score is 4.01 with a standard deviation of 1.529. Here, 69.0% of respondents strongly agree that the information is relevant to their business needs, whereas 10.7% strongly disagree. The high agreement level indicates that the information provided is pertinent and useful for business operations. This is crucial for SMEs, as relevant information aids in identifying market opportunities and threats [20]. The mean score for the availability of information regarding changes in the business environment is 4.15 with a standard deviation of 1.424. Among respondents, 72.6% strongly agree that the information helps them stay informed about environmental changes, while 7.1% strongly disagree. Staying updated with the business environment is essential for SMEs to adapt to market dynamics and regulatory changes. Effective information dissemination helps businesses remain competitive and responsive [21]. The mean score for the availability of information about business registration requirements is 4.08 with a standard deviation of 1.479. A substantial 70.8% of respondents strongly agree that they are well-informed about registration requirements, while 8.9% strongly disagree. Knowledge of registration processes is critical for compliance and legal operations. Clear and accessible information on regulatory requirements facilitates smoother business setup and operation [22]. The mean score for the timeliness of necessary information for business growth is 3.93 with a standard deviation of 1.581. Here, 67.0% of respondents strongly agree that they receive necessary information on time, while 12.8% strongly disagree. Timely information is vital for making informed decisions that drive business growth and development. Delays in information dissemination can hinder business planning and execution [23].

**Table 5:** Descriptive statistics on business information service (means and standard Deviations).

Item Variables		Cases in percent & Frequency					Mean	SD
		Strongly disagree	Disagree	Neutral	Agree	Strongly agree		
Business information is readily available to us	F	9	53	24	0	250	4.28	1.271
	%	2.7	15.8	7.1	0.0	74.4		
the information available is relevant for our business	F	36	53	15	0	232	4.01	1.529
	%	10.7	15.8	4.5	0.0	69.0		
The information available informs us of the changes in the business environment	F	24	53	15	0	244	4.15	1.424
	%	7.1	15.8	4.5	0.0	72.6		
The information available inform us of the business registration requirements	F	30	53	15	0	238	4.08	1.479
	%	8.9	15.8	4.5	0.0	70.8		
The information necessary for our business growth is availed on time	F	43	53	15	0	225	3.93	1.581
	%	12.8	15.8	4.5	0.0	67.0		

According to Table 6, the majority of respondents feel well-prepared to face changes in the business environment and plan appropriate technological changes, as indicated by a mean score of 4.09 and a standard deviation of 1.463. Specifically, 70.8% of respondents strongly agree with this statement, while only 8.0% strongly disagree. This high level of confidence suggests that many SMEs are proactive in adapting to technological advancements and market shifts. Effective preparedness is crucial for maintaining competitiveness and leveraging new opportunities [23]. A significant 70.8% of respondents strongly agree that the most significant challenge in improving SME performance is the lack of adequate managerial skills, with a mean score of 4.08 and a standard deviation of 1.479. The high agreement level underscores the critical role of managerial competencies in driving business success. Recent studies highlight that managerial skills, including strategic planning, financial management, and leadership, are essential for the sustainability and growth of SMEs [22]. The mean score for the impact of a lack of managerial experience on business performance is 3.96 with a standard deviation of 1.559. Among respondents, 67.9% strongly agree that inadequate managerial experience negatively affects performance, while 11.9% strongly disagree. This finding indicates that managerial experience is a key factor in business operations, affecting decision-making, problem-solving, and strategic execution [20]. Respondents strongly agree that management provision of training and development programs affects SME performance, with a mean score of 4.20 and a standard deviation of 1.384. A substantial 73.8% strongly agree with this statement, suggesting that continuous professional development is seen as vital for enhancing managerial capabilities and overall business performance. Training programs help managers stay updated with industry trends and best practices, thus fostering a more competent and efficient workforce [21]. The mean score for the reliance on the effective execution of basic managerial functions for improving business performance is 3.89, with a standard deviation of 1.601. Here, 66.1% of respondents strongly agree that planning, organizing, staffing, directing, and controlling are fundamental to business success, while 13.7% strongly disagree. This finding emphasizes the importance of foundational managerial practices in achieving operational efficiency and strategic goals [19].

**Table 6:** Descriptive statistics on manager experience effect (means and standard Deviations).

Item Variables		Cases in percent & Frequency					Mean	SD
		Strongly disagree	Disagree	Neutral	Agree	Strongly agree		
We are well prepared to face changes in the business environment and to plan appropriate changes in technology	F	27	56	15	0	238	4.09	1.463
	%	8.0	16.7	4.5	0.0	70.8		
The most significant challenge in improving the performance of SMEs is the lack of adequate managerial skills	F	30	53	15	0	238	4.08	1.479
	%	8.9	15.8	4.5	0.0	70.8		
Lack of managerial experience affects the performance of a business	F	40	53	15	0	228	3.96	1.559
	%	11.9	15.8	4.5	0.0	67.9		
management provision of training and development program affects performance of sme	F	20	53	15	0	248	4.20	1.384
	%	6.0	15.8	4.5	0.0	73.8		
Improving business performance relies heavily on the effective execution of basic managerial functions, including planning, organizing, staffing, directing, and controlling	F	46	53	15	0	222	3.89	1.601
	%	13.7	15.8	4.5	0.0	66.1		

Table 7 The majority of respondents strongly agree that the available sources of capital for their business, including bank loans, savings from financial institutions, and contributions from family and friends, are adequate and meet the financial requirements of their enterprise. This is indicated by a high mean score of 4.45 and a standard deviation of 1.155, with 81.0% of respondents strongly agreeing and only 1.8% strongly disagreeing. This high level of agreement suggests that access to financial resources is perceived as sufficient for business operations, which is crucial for the sustainability and growth of SMEs [16]. Effective financial communication within the organization is deemed important, with a mean score of 4.06 and a standard deviation of 1.496. About 70.2% of respondents strongly agree that financial communication promotes transparency and enables informed decision-making, while 9.5% strongly disagree. Effective communication is vital for financial management, as it ensures that information is shared clearly and efficiently, thereby supporting strategic decisions [22]. The financial control mechanisms’ ability to assure the statement reconciliation process has a mean score of 4.39 and a standard deviation of 1.227. Here, 79.5% of respondents strongly agree, indicating strong confidence in their financial control mechanisms, while only 3.3% strongly disagree. Effective reconciliation processes are critical for maintaining accurate financial records and ensuring the integrity of financial data [23]. The mean score for the assurance that all transactions are initiated and posted within a reasonable period is 4.36, with a standard deviation of 1.266. A substantial 78.6% of respondents strongly agree that their financial control mechanisms ensure timely transaction postings, while 4.2% strongly disagree. Timely recording of transactions is essential for accurate financial reporting and operational efficiency [20]. The reliability and integrity of financial information ensured by financial control mechanisms have a mean score of 4.10 and a standard deviation of 1.461. Among respondents, 71.4% strongly agree with this statement, while 8.3% strongly disagree. This indicates a high level of trust in the financial control systems in place, which is fundamental for sound financial management and stakeholder confidence [21].

**Table 7:** Descriptive statistics on Financial control mechanism (means and standard deviations).

Item Variables		Cases in percent & Frequency					Mean	SD
		Strongly disagree	Disagree	Neutral	Agree	Strongly agree		
The available sources of capital for your business, including bank loans, savings from financial institutions, and contributions from family and friends, are deemed to be adequate and meet the financial requirements of your enterprise	F	6	44	14	0	272	4.45	1.155
	%	1.8	13.1	4.2	0.0	81.0		
Effective financial communication within the organization ensures that information is clearly and efficiently shared, promoting transparency and enabling informed decision-making processes to occur smoothly	F	32	53	15	0	236	4.06	1.496
	%	9.5	15.8	4.5	0.0	70.2		
Our financial control mechanism assure the statement reconciliation process	F	11	44	14	0	267	4.39	1.227
	%	3.3	13.1	4.2	0.0	79.5		
verify that all transactions are initiated posted in a reasonable period	F	14	44	14	0	264	4.36	1.266
	%	4.2	13.1	4.2	0.0	78.6		
The financial controlling mechanism ensure the reliability and integrity of financial information	F	28	53	15	0	240	4.10	1.461
	%	8.3	15.8	4.5	0.0	71.4		

Table 8 shows that, the perception of an inadequate market for products is high, with a mean score of 4.27 and a standard deviation of 1.350. A significant 76.5% of respondents strongly agree that their products face an inadequate market, while only 6.3% strongly disagree. This suggests that a large majority of SMEs struggle with market demand for their products. Recent studies highlight that inadequate market demand can hinder business growth and sustainability, emphasizing the need for market development strategies [19]. The difficulty in searching for new markets for products is reflected in a mean score of 4.15 and a standard deviation of 1.454. Here, 73.5% of respondents strongly agree that finding new markets is challenging, while 9.2% strongly disagree. This indicates a prevalent struggle among SMEs to expand their market reach, which is crucial for business expansion and profitability. Effective market research and development initiatives are essential to overcome these challenges [22]. The perception that the quality of products is not up to market standards has a mean score of 4.23 and a standard deviation of 1.394. A substantial 75.3% of respondents strongly agree with this statement, indicating concerns about meeting market quality requirements. Only 7.4% strongly disagree. This highlights the importance of quality control and assurance processes in maintaining competitive advantage and customer satisfaction [23]. The deficiency in establishing a network within the market has a mean score of 4.10 and a standard deviation of 1.499. Among respondents, 72.0% strongly agree that networking within the market is deficient, while 10.7% strongly disagree. Effective networking is critical for market penetration and business collaborations, which can significantly impact business growth and success [20]. Insufficient promotion efforts hindering the attraction of potential users have a mean score of 4.02 and a standard deviation of 1.549. A notable 70.2% of respondents strongly agree that their promotional efforts are inadequate, while 12.5% strongly disagree. This suggests that many SMEs recognize the need for improved marketing and promotional strategies to attract and retain customers. Investing in marketing initiatives is essential for increasing market visibility and customer base [21].

**Table 8:** Descriptive statistics on Marketing Skill and Strategy Factor (means and standard deviations).

Item Variables		Cases in percent & Frequency					Mean	SD
		Strongly disagree	Disagree	Neutral	Agree	Strongly agree		
Inadequate market for my product	F	21	44	14	0	257	4.27	1.350
	%	6.3	13.1	4.2	0.0	76.5		
Searching new market for our products is so difficult	F	31	44	14	0	247	4.15	1.454
	%	9.2	13.1	4.2	0.0	73.5		
The quality of my products is not up to the standards required for the market	F	25	44	14	0	253	4.23	1.394
	%	7.4	13.1	4.2	0.0	75.3		
There is a deficiency in establishing a network within the market	F	36	44	14	0	242	4.10	1.499
	%	10.7	13.1	4.2	0.0	72.0		
Insufficient promotion efforts are hindering the attraction of potential users	F	42	44	14	0	236	4.02	1.549
	%	12.5	13.1	4.2	0.0	70.2		

Table 9 The profitability of businesses is perceived positively by the majority of respondents, with a mean score of 4.12 and a standard deviation of 1.481. Specifically, 72.6% of respondents strongly agree that their business is profitable, while 10.1% strongly disagree. This indicates a generally optimistic view of business profitability among SMEs. Profitable operations are essential for business sustainability and growth, and this positive perception suggests that many SMEs are effectively managing their financial resources [22]. The perception of sales turnover is more varied, with a mean score of 3.59 and a standard deviation of 1.734. About 58.6% of respondents strongly agree that their business has a good sales turnover, while 21.1% strongly disagree. This indicates mixed experiences among SMEs regarding sales performance, which could be influenced by market demand, competition, and marketing effectiveness. Improving sales strategies and market outreach can help enhance sales turnover [23]. The perception of progress in reducing business challenges has a mean score of 3.63 and a standard deviation of 1.721. Here, 59.5% of respondents strongly agree that their business is making progress in overcoming challenges, while 20.2% strongly disagree. This suggests that while many SMEs see improvements, a significant portion still faces considerable obstacles. Addressing these challenges through better management practices and supportive policies can aid in further progress [20].

**Table 9:** Descriptive statistics on current performance of MSE based on business practice.

Item Variables		Cases in percent & Frequency					Mean	SD
		Strongly disagree	Disagree	Neutral	Agree	Strongly agree		
My business is Profitable	F	34	44	14	0	244	4.12	1.481
	%	10.1	13.1	4.2	0.0	72.6		
My business has a good Sales turnover	F	71	53	15	0	197	3.59	1.734
	%	21.1	15.8	4.5	0.0	58.6		
My business is showing progress in reducing	F	68	53	15	0	200	3.63	1.721
	%	20.2	15.8	4.5	0.0	59.5		

Table 10 The regression analysis evaluates the impact of various factors—Financial, Government, Business, Market, and Management—on the performance of Small and Medium-Sized Enterprises (SMSEs). The summary of the regression results includes 336 observations, an F-statistic of 259.26, a Prob >  F of 0.0000, an R-squared of 0.7971, an adjusted R-squared of 0.7940, and a root MSE of 0.55034. The high F-statistic value (259.26) and the Prob >  F (0.0000) indicate that the overall regression model is statistically significant, meaning the predictors collectively have a significant impact on the performance of SMSEs. The R-squared (0.7971) and adjusted R-squared (0.7940) values suggest that approximately 79.71% of the variance in the performance of SMSEs can be explained by the independent variables included in the model. The adjusted R-squared value, which adjusts for the number of predictors in the model, is also high, reinforcing the model’s robustness. The performance of SMSEs is highly correlated with most factors. The strongest correlations are observed with Business Information Services (0.8785), Financial Control (0.8577), and Management Experience (0.8571). This suggests that these factors play a significant role in enhancing SMSE performance. Government Policy and Regulation also show a strong positive correlation (0.8469) with SMSE performance, indicating that supportive government policies are crucial for the success of SMSEs. The correlation between Marketing Skill and Strategy and SMSE performance is moderate (0.5666), implying that while marketing skills are important, and other factors may have a more substantial impact on performance. There are very high inter-correlations among Business Information Services, Management Experience, and Financial Control factors. For instance, the correlation between Management Experience and Financial Control is exceptionally high (0.9699), indicating these factors are closely related and often influence each other. Government Policy and Regulation are also strongly correlated with Business Information Services (0.9176), Management Experience (0.8718), and Financial Control (0.8739). This demonstrates the pervasive impact of government policies across various operational aspects of SMSEs. Marketing Skill and Strategy, while moderately correlated with the other factors, has the weakest correlations overall, with the highest being with Business Information Services (0.6930). This suggests that while marketing is important, it might operate somewhat independently compared to other factors. The high correlation coefficients between the performance of SMSEs and factors such as Business Information Services, Financial Control, and Management Experience underscore the critical importance of these elements in driving business success. The data suggests that enhancing these areas could lead to substantial improvements in SMSE performance. For instance, investing in robust financial control mechanisms and ensuring access to relevant business information can provide a solid foundation for strategic decision-making and operational efficiency [23]. Moreover, the strong influence of Government Policy and Regulation on SMSE performance highlights the necessity for a supportive regulatory environment. Policies that facilitate access to finance, simplify business registration processes, and provide necessary business support services can significantly bolster SMSE growth and sustainability [16]. The moderate correlation with Marketing Skill and Strategy suggests that while marketing is essential, its impact might be more nuanced and dependent on the effective execution of the other core factors. Effective marketing strategies need to be backed by strong management practices and financial controls to realize their full potential [22].

**Table 10:** Correlation between dependent and independents variables.

Variables	PSMSEs	GPRF	BISF	MEF	FCF	MSSF
PSMSEs	1.0000					
GPRF	0.8469	1.0000				
BISF	0.8785	0.9176	1.0000			
MEF	0.8571	0.8718	0.9118	1.0000		
FCF	0.8577	0.8739	0.9461	0.9699	1.0000	
MSSF	0.5666	0.4765	0.6930	0.6216	0.6924	1.0000

In conclusion, the correlation analysis reveals that Business Information Services, Financial Control, and Management Experience are pivotal in enhancing SMSE performance. Government policies also play a crucial role, while marketing strategies, though important; appear to be less influential in isolation. Addressing these key areas can significantly improve the operational and financial performance of SMSEs.

Table 11 The marginal effects measure the change in the dependent variable (performance of SMSEs) for a one-unit change in each independent variable, holding all other variables constant. This helps to understand the direct impact of each factor on the performance of SMSEs. The financial factor has a coefficient of 0.1442 and a p-value of 0.035, indicating a positive and statistically significant impact on the performance of SMSEs. A unit increase in the financial factor is associated with a 0.1442 increase in the performance score. This underscores the importance of adequate financial resources and sound financial management in enhancing business performance. Government policy and regulation are also highly significant and positively associated with SMSE performance, with a coefficient of 0.5272 and a p-value of 0.000. A unit increase in this factor leads to a 0.5272 increase in performance. Supportive government policies, such as favorable regulations and tax policies play a crucial role in fostering conducive environment for SMEs to thrive [16]. The business information services factor significantly enhances SMSE performance, with a coefficient of 0.3429 and a p-value of 0.000. A unit increase in this factor results in a 0.3429 increase in performance. Access to relevant business information is critical for strategic decision-making and operational efficiency [22]. The market factor, with a coefficient of -0.1460 and a p-value of 0.238, is not statistically significant. This indicates that the perceived difficulty in accessing markets or market-related issues does not have a substantial direct impact on SMSE performance in this model. Other factors might mediate the relationship between market challenges and performance. Management experience has a coefficient of -0.0112 and a p-value of 0.785, indicating it does not have a statistically significant impact on SMSE performance in this model. This could imply that while management experience is important, its effect might be overshadowed by other factors such as financial control and government policies in this specific context.

**Table 11:** Regression result.

Table 11 a. ANOVA Table of the analysis						
Source	SS	df	MS	No. of obs = 336, F (5, 330) = 259.26		
Model	392.61228	5	78.5224561	Prob > F = 0.0000		
Residual	99.9472435	330	.302870435	R-squared = 0.7971		
Total	492.559524	335	1.47032694	Root MSE = .55034		
				Adj-R-squared = 0.7940		
**Table 11 b. Regression Coefficients analysis**						
**Performance of SMSEs**	Coef.	Std. Err.	t	P > |t|	[95% Conf. Interval] (Lower, Upper)	
Financial	.1441613	.068206	2.11	0.035	.0099879	.2783347
Government	.5271884	.1095769	4.81	0.000	.3116311	.7427457
Business	.3429426	.0925056	3.71	0.000	.1609676	.5249176
Market	-.1459598	.1235944	-1.18	0.238	-.3890921	.0971726
Management	-.0112346	.0411632	-0.27	0.785	-.0922101	.0697408
_cons	.5753977	.1204958	4.78	0.000	.3383609	.8124345

In conclusion, the regression analysis highlights the significant roles of financial resources, government policies, and business information services in enhancing the performance of SMSEs. These findings suggest that improving access to financial capital, ensuring supportive regulatory environments, and providing relevant business information can substantially boost SME performance. Conversely, market challenges and management experience, while important; do not show a significant direct impact within this model. This indicates the need for a holistic approach that considers multiple facets of business operations to drive SMSE success.

Table 12 The marginal effects measure the change in the performance of SMSEs for a one-unit change in each independent variable, holding all other variables constant. This helps to understand the direct impact of each factor on the performance of SMSEs. A one-unit increase in the financial factor is associated with a 0.1442 unit increase in the performance of SMSEs, holding other factors constant. This effect is statistically significant (p-value =  0.035), indicating that financial resources are crucial for improving business performance. A one-unit increase in the government policy and regulation factor leads to a 0.5272 unit increase in SMSE performance, holding other factors constant. This effect is highly significant (p-value =  0.000), underscoring the importance of supportive government policies in enhancing business performance [16]. A one-unit increase in the business information services factor results in a 0.3429 unit increase in the performance of SMSEs, holding other factors constant. This effect is also highly significant (p-value =  0.000), highlighting the critical role of access to relevant business information in driving performance [22]. A one-unit increase in the market factor is associated with a 0.1460 unit decrease in the performance of SMSEs, holding other factors constant. This effect is not statistically significant (p-value =  0.238), suggesting that market-related challenges might not have a direct impact on performance when other factors are controlled. A one-unit increase in the management experience factor results in a 0.0112 unit decrease in the performance of SMSEs, holding other factors constant. This effect is not statistically significant (p-value =  0.785), indicating that management experience alone might not significantly influence performance when considering other factors. The marginal effects analysis reinforces the significant roles of financial resources, government policies, and business information services in enhancing the performance of SMSEs. These factors have positive and substantial impacts, suggesting that improving financial access, implementing supportive policies, and providing relevant information can lead to significant improvements in business performance. Access to adequate financial resources enables SMSEs to invest in essential areas such as technology, infrastructure, and human capital, thereby enhancing their operational efficiency and competitiveness [23]. Supportive government policies and regulations create conducive environment for business growth. This includes simplified business registration processes, favorable tax policies, and access to grants and subsidies [16]. Additionally, access to timely and relevant business information helps SMSEs make informed decisions, identify market opportunities, and respond to market changes effectively. This is crucial for strategic planning and operational efficiency [22].

**Table 12:** Marginal effect.

Variables	Delta-method					
	dy/dx	Std. Err.	t	P>|t |	[95% Conf. Interval]	
Financial	.1441613	.068206	2.11	0.035	.0099879	.2783347
Government	.5271884	.1095769	4.81	0.000	.3116311	.7427457
Business	.3429426	.0925056	3.71	0.000	.1609676	.5249176
Market	-.1459598	.1235944	-1.18	0.238	-.3890921	.0971726
Management	-.0112346	.0411632	-0.27	0.785	-.0922101	.0697408

margins,dydx(Financial, Government, Business, Market, Management).

Average marginal effects Number of obs =  336.

Model VCE: OLS.

Expression: Linear prediction, predict().

dy/dx w.r.t.: Financial, Government, Business, Market, Management.

**Fig 1:**
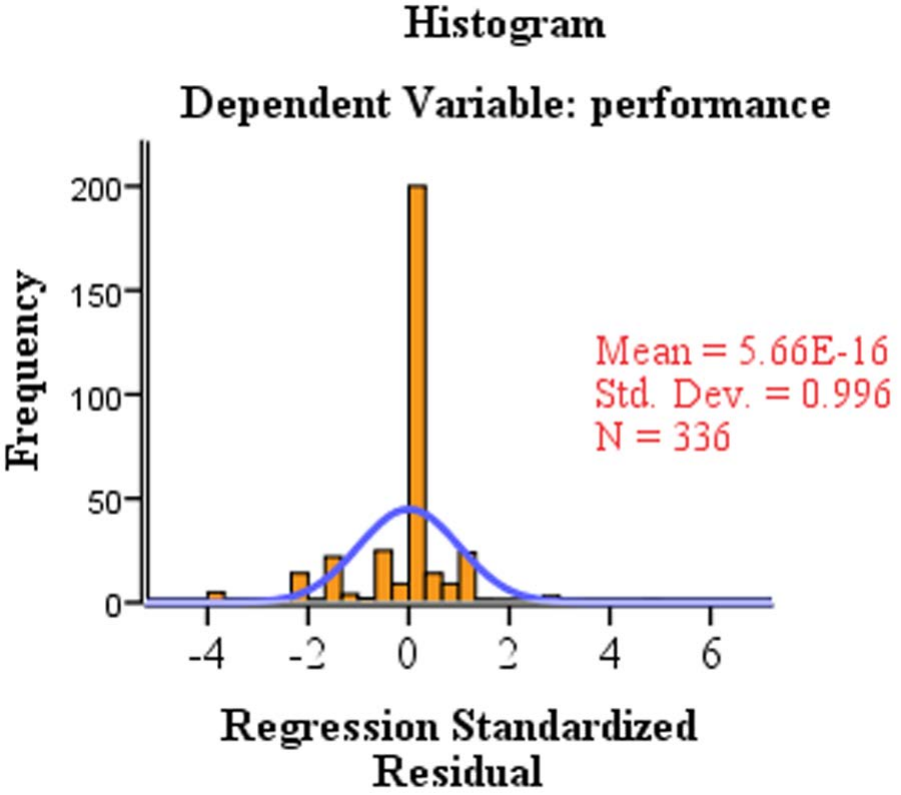
Histogram of regression standardized residual for dependent variable: performance.

Fig 1 Histogram of regression standardized residual for dependent variable: performance. The marginal effects analysis highlights the significant influence of financial resources, government policies, and business information services on the performance of SMSEs. These factors are critical for driving business success and should be prioritized in policies and strategic initiatives aimed at supporting SMSEs. Conversely, market challenges and management experience, while important; do not show a significant direct impact on performance within this model.

## Conclusions

The main goal of this study was to evaluate the relationship between various factors and the performance of MSE operators in Fireweyni town manufacturing, construction, trade service, and urban agriculture sectors. Specifically, the study aimed to investigate the connections between MSE performance and financial control systems, management experience, business information services, and government regulation. The study’s findings led to the following conclusions.

Consistent with the findings, the correlation analysis revealed that every variable (factor) incorporated in this study was statistically significant, impacting the performance of SMEs and businesses within the study area. The Pearson correlation coefficient test results showed that all independent variables have a significant relationship with MSE business performance. One variable had a coefficient range of ± 0.41 to ± 0.70, indicating a moderate relationship with the dependent variable. Four variables had a coefficient range of ± 0.71 to ± 0.90, indicating a high relationship with the dependent variable. Specifically, management factors had a value of 0.8571, government regulation was 0.849, business information service value was 0.8785, marketing skill and strategy value was 0.5666, financial control mechanism value was 0.8577, and marketing strategy value was 0.815.

## Recommendations

The study’s conclusions indicate that financial control, marketing strategy, managerial expertise, government regulations, and business information services significantly affect the performance of small and micro businesses in Fireweyni City. Therefore, when developing plans to enhance their performance, managers, directors, government agencies, and other stakeholders should consider these factors.

- **Management Experience**: Businesses operated by individuals with prior management experience perform better than those without such expertise. Therefore, MSE Enterprise and other government organizations should focus on developing management training programs and establishing opportunities for experience sharing, particularly for newcomers to the industry.
- **Marketing Skills**: MSE operators should improve their marketing abilities through appropriate training and experience sharing with successful medium- and large-scale businesses. Government agencies, NGOs, sub-city micro and small enterprise agencies, and other stakeholders can support these operators by helping them market their products effectively. This includes competitive pricing, fostering positive customer relationships, and promoting their outputs.
- **Market Assessments**: Conducting market assessments is crucial for addressing the challenges of launching and establishing MSE businesses. These assessments can help identify and rank the types of MSE businesses, pinpoint input sources, and find suitable locations. Additionally, MSEs need training and guidance on developing a saving culture and generating initial capital.
- **Funding**: Most MSE operators in the research area primarily obtain funding from microfinance banks through loans. However, the informal sector cannot provide as much credit as needed, and the formal sector (banks) often requires collateral. Therefore, highlighting the informal sector’s role is essential.
- **Capacity Building**: To make MSEs competitive and profitable, the government should enhance the operators’ capacity, knowledge, and skills, share experiences from successful enterprises, and provide advice and consultancy. Continuous capacity-building initiatives and access to relevant technologies should also be available.
- **Government Support**: The government should play a leading role in educating SME practitioners about available incentives and how to access them. These incentives should be delivered through establishments that genuinely care about the success and sustainability of SMEs in the country.

## Supporting information

S1 File Raw Data SME 2024.
(DOCX)

## Abbreviations

MSEs: Micro and small enterprises
SME: small micro enterprise
NGO: non-governmental organization
OLS: ordinary least square.
