# Supplementary material for: Assessment of performance and challenges of small and micro enterprises: Fireweyni town, Ethiopia
Source: PLoS One. 2025 Apr 8;20(4):e0320681. doi: 10.1371/journal.pone.0320681 (PMC11978033; doi:10.1371/journal.pone.0320681)
Supplement: S1 File — Raw Data SME 2024. (DOCX) [file pone.0320681.s001.docx]

| Gender | Age | Education | type | duration | govt1 | govt2 | gov3 | govt4 | BUS1 | BUS2 | BUS3 | BUS4 |
| --- | --- | --- | --- | --- | --- | --- | --- | --- | --- | --- | --- | --- |
| male | above 41 | Education level below 10 | urban agriculture | less than 1 year | strongly agree | strongly agree | strongly agree | strongly agree | strongly agree | strongly disagree | strongly disagree | strongly disagree |
| male | 34-41 years | Education level below 10 | urban agriculture | less than 1 year | strongly agree | strongly agree | strongly agree | strongly agree | strongly agree | strongly disagree | strongly disagree | strongly disagree |
| male | 18-25 years | BA degree | construction | less than 1 year | strongly agree | strongly agree | strongly agree | strongly agree | strongly agree | strongly disagree | strongly disagree | strongly disagree |
| male | 34-41 years | BA degree | Trade | less than 1 year | strongly agree | strongly agree | strongly agree | strongly agree | strongly agree | strongly disagree | strongly disagree | strongly disagree |
| male | 18-25 years | Earned a deploma from a recognized institution | construction | less than 1 year | strongly agree | strongly agree | strongly agree | strongly agree | strongly agree | strongly disagree | strongly disagree | strongly disagree |
| male | 34-41 years | Earned a deploma from a recognized institution | manufacturing | less than 1 year | strongly agree | strongly agree | strongly agree | strongly agree | strongly agree | strongly disagree | strongly disagree | strongly disagree |
| female | 34-41 years | BA degree | Trade | less than 1 year | strongly agree | strongly agree | strongly agree | strongly agree | strongly agree | strongly disagree | strongly disagree | strongly disagree |
| male | 26-33 years | BA degree | construction | less than 1 year | strongly agree | strongly agree | strongly agree | strongly agree | strongly agree | strongly disagree | strongly disagree | strongly disagree |
| female | 26-33 years | BA degree | Trade | less than 1 year | strongly agree | strongly agree | strongly agree | strongly agree | strongly agree | strongly disagree | strongly disagree | strongly disagree |
| female | 26-33 years | BA degree | manufacturing | less than 1 year | strongly agree | strongly agree | strongly agree | strongly agree | strongly agree | strongly disagree | strongly disagree | strongly disagree |
| male | 26-33 years | finished Ethiopian Secondary school 10+3 | Trade | less than 1 year | strongly agree | strongly agree | strongly agree | strongly agree | strongly agree | strongly disagree | strongly disagree | strongly disagree |
| male | 34-41 years | Education level below 10 | urban agriculture | less than 1 year | strongly agree | strongly agree | strongly agree | strongly agree | strongly agree | strongly disagree | strongly disagree | strongly disagree |
| male | 18-25 years | finished Ethiopian Secondary school 10+3 | Trade | less than 1 year | strongly agree | strongly agree | strongly agree | strongly agree | strongly agree | strongly disagree | strongly disagree | strongly disagree |
| male | 18-25 years | finished Ethiopian Secondary school 10+3 | manufacturing | less than 1 year | strongly agree | strongly agree | strongly agree | strongly agree | strongly agree | strongly disagree | strongly disagree | strongly disagree |
| male | 34-41 years | finished Ethiopian Secondary school 10+3 | Trade | less than 1 year | strongly agree | strongly agree | strongly agree | strongly agree | strongly agree | strongly disagree | strongly disagree | strongly disagree |
| female | 18-25 years | finished Ethiopian Secondary school 10+3 | urban agriculture | less than 1 year | strongly agree | strongly agree | strongly agree | strongly agree | strongly agree | strongly disagree | strongly agree | strongly disagree |
| female | 18-25 years | finished Ethiopian Secondary school 10+3 | urban agriculture | less than 1 year | strongly agree | strongly agree | strongly agree | strongly agree | strongly agree | strongly disagree | strongly agree | strongly disagree |
| female | 18-25 years | finished Ethiopian Secondary school 10+3 | manufacturing | 4-6 years | strongly agree | strongly agree | strongly agree | strongly agree | strongly agree | strongly disagree | strongly agree | strongly disagree |
| male | 34-41 years | finished Ethiopian Secondary school 10+3 | Trade | less than 1 year | strongly agree | strongly agree | strongly agree | strongly agree | strongly agree | strongly disagree | strongly agree | strongly disagree |
| female | 34-41 years | finished Ethiopian Secondary school 10+3 | manufacturing | less than 1 year | strongly agree | strongly agree | strongly agree | strongly agree | strongly agree | strongly disagree | strongly agree | strongly disagree |
| female | 34-41 years | finished Ethiopian Secondary school 10+3 | Trade | 4-6 years | strongly agree | strongly agree | strongly agree | strongly agree | strongly agree | strongly disagree | strongly agree | strongly disagree |
| male | 34-41 years | BA degree | manufacturing | less than 1 year | strongly agree | strongly agree | strongly agree | strongly agree | strongly agree | strongly disagree | strongly agree | strongly agree |
| male | 26-33 years | finished Ethiopian Secondary school 10+3 | Trade | 4-6 years | strongly agree | strongly agree | strongly agree | strongly agree | strongly agree | strongly disagree | strongly agree | strongly agree |
| male | 26-33 years | BA degree | Trade | less than 1 year | strongly agree | strongly agree | strongly agree | strongly agree | strongly agree | strongly disagree | strongly agree | strongly agree |
| male | 26-33 years | BA degree | Trade | less than 1 year | strongly agree | strongly agree | strongly agree | strongly agree | strongly agree | strongly disagree | strongly agree | strongly agree |
| male | 34-41 years | BA degree | Trade | above 6 years | strongly agree | strongly agree | strongly agree | strongly agree | strongly agree | strongly disagree | strongly agree | strongly agree |
| male | 34-41 years | Earned a deploma from a recognized institution | Trade | less than 1 year | strongly agree | strongly agree | strongly agree | strongly agree | strongly agree | strongly disagree | strongly agree | strongly agree |
| male | 34-41 years | BA degree | manufacturing | less than 1 year | strongly agree | strongly agree | strongly agree | strongly agree | strongly agree | strongly agree | strongly agree | strongly agree |
| female | 34-41 years | BA degree | Trade | less than 1 year | strongly agree | strongly agree | strongly agree | strongly agree | strongly agree | strongly agree | strongly agree | strongly agree |
| female | 34-41 years | BA degree | Trade | 4-6 years | strongly agree | strongly agree | strongly agree | strongly agree | strongly agree | strongly agree | strongly agree | strongly agree |
| female | above 41 | BA degree | Trade | less than 1 year | strongly agree | strongly agree | strongly agree | strongly agree | strongly agree | strongly agree | strongly agree | strongly agree |
| female | above 41 | BA degree | service | 4-6 years | strongly agree | strongly agree | strongly agree | strongly agree | strongly agree | strongly agree | strongly agree | strongly agree |
| male | above 41 | Education level below 10 | urban agriculture | above 6 years | strongly agree | strongly agree | strongly agree | strongly agree | strongly agree | strongly agree | strongly agree | strongly agree |
| male | 18-25 years | Education level below 10 | urban agriculture | above 6 years | strongly agree | strongly agree | strongly agree | strongly agree | strongly agree | strongly agree | strongly agree | strongly agree |
| female | 26-33 years | Education level below 10 | urban agriculture | above 6 years | strongly agree | strongly agree | strongly agree | strongly agree | strongly agree | strongly agree | strongly agree | strongly agree |
| male | 18-25 years | Education level below 10 | urban agriculture | above 6 years | strongly agree | strongly agree | strongly agree | strongly agree | strongly agree | strongly agree | strongly agree | strongly agree |
| female | 18-25 years | Education level below 10 | urban agriculture | above 6 years | strongly agree | strongly agree | strongly agree | strongly agree | strongly agree | strongly agree | strongly agree | strongly agree |
| male | 18-25 years | Education level below 10 | Trade | less than 1 year | strongly agree | strongly agree | strongly agree | strongly agree | strongly agree | strongly agree | strongly agree | strongly agree |
| female | 34-41 years | Education level below 10 | urban agriculture | 4-6 years | strongly agree | strongly agree | strongly agree | strongly agree | strongly agree | strongly agree | strongly agree | strongly agree |
| male | 18-25 years | finished Ethiopian Secondary school 10+3 | Trade | 4-6 years | strongly agree | strongly agree | strongly agree | strongly agree | strongly agree | strongly agree | strongly agree | strongly agree |
| male | 18-25 years | Education level below 10 | Trade | less than 1 year | strongly agree | strongly agree | strongly agree | strongly agree | strongly agree | strongly agree | strongly agree | strongly agree |
| male | 18-25 years | Education level below 10 | Trade | less than 1 year | strongly agree | strongly agree | strongly agree | strongly agree | strongly agree | strongly agree | strongly agree | strongly agree |
| male | 26-33 years | Education level below 10 | Trade | 4-6 years | strongly agree | strongly agree | strongly agree | strongly agree | strongly agree | strongly agree | strongly agree | strongly agree |
| male | 26-33 years | finished Ethiopian Secondary school 10+3 | Trade | less than 1 year | strongly agree | strongly agree | strongly agree | strongly agree | strongly agree | strongly agree | strongly agree | strongly agree |
| male | 26-33 years | Earned a deploma from a recognized institution | manufacturing | less than 1 year | strongly agree | strongly agree | strongly agree | strongly agree | strongly agree | strongly agree | strongly agree | strongly agree |
| male | 26-33 years | Education level below 10 | Trade | less than 1 year | strongly agree | strongly agree | strongly agree | strongly agree | strongly agree | strongly agree | strongly agree | strongly agree |
| male | 26-33 years | finished Ethiopian Secondary school 10+3 | Trade | less than 1 year | strongly agree | strongly agree | strongly agree | strongly agree | strongly agree | strongly agree | strongly agree | strongly agree |
| male | 26-33 years | Education level below 10 | manufacturing | 4-6 years | strongly agree | strongly agree | strongly agree | strongly agree | strongly agree | strongly agree | strongly agree | strongly agree |
| male | 26-33 years | finished Ethiopian Secondary school 10+3 | Trade | less than 1 year | strongly agree | strongly agree | strongly agree | strongly agree | strongly agree | strongly agree | strongly agree | strongly agree |
| male | above 41 | finished Ethiopian Secondary school 10+3 | Trade | less than 1 year | strongly agree | strongly agree | strongly agree | strongly agree | strongly agree | strongly agree | strongly agree | strongly agree |
| male | above 41 | finished Ethiopian Secondary school 10+3 | manufacturing | 4-6 years | strongly agree | strongly agree | strongly agree | strongly agree | strongly agree | strongly agree | strongly agree | strongly agree |
| male | 34-41 years | finished Ethiopian Secondary school 10+3 | Trade | 4-6 years | strongly agree | strongly agree | strongly agree | strongly agree | strongly agree | strongly agree | strongly agree | strongly agree |
| male | 18-25 years | finished Ethiopian Secondary school 10+3 | manufacturing | less than 1 year | strongly agree | strongly agree | strongly agree | strongly agree | strongly agree | strongly agree | strongly agree | strongly agree |
| male | 34-41 years | finished Ethiopian Secondary school 10+3 | Trade | less than 1 year | strongly agree | strongly agree | strongly agree | strongly agree | strongly agree | strongly agree | strongly agree | strongly agree |
| male | 34-41 years | finished Ethiopian Secondary school 10+3 | manufacturing | 4-6 years | strongly agree | strongly agree | strongly agree | strongly agree | strongly agree | strongly agree | strongly agree | strongly agree |
| female | 18-25 years | finished Ethiopian Secondary school 10+3 | urban agriculture | 4-6 years | strongly agree | strongly agree | strongly agree | strongly agree | strongly agree | strongly agree | strongly agree | strongly agree |
| male | 34-41 years | Education level below 10 | Trade | 4-6 years | strongly agree | strongly agree | strongly agree | strongly agree | strongly agree | strongly agree | strongly agree | strongly agree |
| male | 18-25 years | finished Ethiopian Secondary school 10+3 | Trade | 4-6 years | strongly agree | strongly agree | strongly agree | strongly agree | strongly agree | strongly agree | strongly agree | strongly agree |
| male | 18-25 years | finished Ethiopian Secondary school 10+3 | manufacturing | less than 1 year | strongly agree | strongly agree | strongly agree | strongly agree | strongly agree | strongly agree | strongly agree | strongly agree |
| male | 26-33 years | finished Ethiopian Secondary school 10+3 | manufacturing | 1-3 years | strongly agree | strongly agree | strongly agree | strongly agree | strongly agree | strongly agree | strongly agree | strongly agree |
| male | 26-33 years | finished Ethiopian Secondary school 10+3 | Trade | less than 1 year | strongly agree | strongly agree | strongly agree | strongly agree | strongly agree | strongly agree | strongly agree | strongly agree |
| male | 18-25 years | finished Ethiopian Secondary school 10+3 | Trade | less than 1 year | strongly agree | strongly agree | strongly agree | strongly agree | strongly agree | strongly agree | strongly agree | strongly agree |
| male | 18-25 years | Education level below 10 | service | less than 1 year | strongly agree | strongly agree | strongly agree | strongly agree | strongly agree | strongly agree | strongly agree | strongly agree |
| male | 18-25 years | Education level below 10 | urban agriculture | less than 1 year | strongly agree | strongly agree | strongly agree | strongly agree | strongly agree | strongly agree | strongly agree | strongly agree |
| female | 26-33 years | Education level below 10 | Trade | 4-6 years | strongly agree | strongly agree | strongly agree | strongly agree | strongly agree | strongly agree | strongly agree | strongly agree |
| male | 26-33 years | Education level below 10 | Trade | less than 1 year | strongly agree | strongly agree | strongly agree | strongly agree | strongly agree | strongly agree | strongly agree | strongly agree |
| male | 26-33 years | Education level below 10 | urban agriculture | 4-6 years | strongly agree | strongly agree | strongly agree | strongly agree | strongly agree | strongly agree | strongly agree | strongly agree |
| male | 34-41 years | Earned a deploma from a recognized institution | manufacturing | 4-6 years | strongly agree | strongly agree | strongly agree | strongly agree | strongly agree | strongly agree | strongly agree | strongly agree |
| male | 34-41 years | Earned a deploma from a recognized institution | manufacturing | less than 1 year | strongly agree | strongly agree | strongly agree | strongly agree | strongly agree | strongly agree | strongly agree | strongly agree |
| male | 34-41 years | Earned a deploma from a recognized institution | Trade | 4-6 years | strongly agree | strongly agree | strongly agree | strongly agree | strongly agree | strongly agree | strongly agree | strongly agree |
| male | 34-41 years | Earned a deploma from a recognized institution | Trade | less than 1 year | strongly agree | strongly agree | strongly agree | strongly agree | strongly agree | strongly agree | strongly agree | strongly agree |
| male | 34-41 years | Earned a deploma from a recognized institution | service | less than 1 year | strongly agree | strongly agree | strongly agree | strongly agree | strongly agree | strongly agree | strongly agree | strongly agree |
| male | 34-41 years | Earned a deploma from a recognized institution | Trade | less than 1 year | strongly agree | strongly agree | strongly agree | strongly agree | strongly agree | strongly agree | strongly agree | strongly agree |
| male | 34-41 years | finished Ethiopian Secondary school 10+3 | Trade | less than 1 year | strongly agree | strongly agree | strongly agree | strongly agree | strongly agree | strongly agree | strongly agree | strongly agree |
| male | 34-41 years | finished Ethiopian Secondary school 10+3 | Trade | above 6 years | strongly agree | strongly agree | strongly agree | strongly agree | strongly agree | strongly agree | strongly agree | strongly agree |
| male | 34-41 years | finished Ethiopian Secondary school 10+3 | service | above 6 years | strongly agree | strongly agree | strongly agree | strongly agree | strongly agree | strongly agree | strongly agree | strongly agree |
| female | above 41 | finished Ethiopian Secondary school 10+3 | Trade | 4-6 years | strongly agree | strongly agree | strongly agree | strongly agree | strongly agree | strongly agree | strongly agree | strongly agree |
| male | above 41 | finished Ethiopian Secondary school 10+3 | Trade | less than 1 year | strongly agree | strongly agree | strongly agree | strongly agree | strongly agree | strongly agree | strongly agree | strongly agree |
| male | above 41 | Education level below 10 | Trade | less than 1 year | strongly agree | strongly agree | strongly agree | strongly agree | strongly agree | strongly agree | strongly agree | strongly agree |
| female | above 41 | finished Ethiopian Secondary school 10+3 | Trade | less than 1 year | strongly agree | strongly agree | strongly agree | strongly agree | strongly agree | strongly agree | strongly agree | strongly agree |
| female | 26-33 years | Education level below 10 | urban agriculture | less than 1 year | strongly agree | strongly agree | strongly agree | strongly agree | strongly agree | strongly agree | strongly agree | strongly agree |
| female | 26-33 years | Education level below 10 | Trade | less than 1 year | strongly agree | strongly agree | strongly agree | strongly agree | strongly agree | strongly agree | strongly agree | strongly agree |
| male | 34-41 years | Education level below 10 | Trade | less than 1 year | strongly agree | strongly agree | strongly agree | strongly agree | strongly agree | strongly agree | strongly agree | strongly agree |
| male | 34-41 years | Education level below 10 | urban agriculture | less than 1 year | strongly agree | strongly agree | strongly agree | strongly agree | strongly agree | strongly agree | strongly agree | strongly agree |
| male | above 41 | Education level below 10 | Trade | less than 1 year | strongly agree | strongly agree | strongly agree | strongly agree | Neutral | strongly agree | strongly agree | strongly agree |
| male | 34-41 years | Education level below 10 | urban agriculture | less than 1 year | strongly agree | strongly agree | strongly agree | strongly agree | Neutral | strongly agree | strongly agree | strongly agree |
| male | 34-41 years | Education level below 10 | urban agriculture | 1-3 years | strongly agree | strongly agree | strongly agree | strongly agree | Neutral | strongly agree | strongly agree | strongly agree |
| male | above 41 | Education level below 10 | urban agriculture | less than 1 year | strongly agree | strongly agree | strongly agree | strongly agree | Neutral | strongly agree | strongly agree | strongly agree |
| male | 34-41 years | Education level below 10 | Trade | less than 1 year | strongly agree | strongly agree | strongly agree | strongly agree | Neutral | strongly agree | strongly agree | strongly agree |
| male | 34-41 years | Education level below 10 | urban agriculture | less than 1 year | strongly agree | strongly agree | strongly agree | strongly agree | Neutral | strongly agree | strongly agree | strongly agree |
| male | 34-41 years | Education level below 10 | urban agriculture | 1-3 years | strongly agree | strongly agree | strongly agree | strongly agree | Neutral | strongly agree | strongly agree | strongly agree |
| male | above 41 | Education level below 10 | Trade | 1-3 years | strongly agree | strongly agree | strongly agree | strongly agree | Neutral | strongly agree | strongly agree | strongly agree |
| male | 34-41 years | Education level below 10 | Trade | less than 1 year | strongly agree | strongly agree | strongly agree | strongly agree | Neutral | strongly agree | strongly agree | strongly agree |
| male | 34-41 years | Education level below 10 | urban agriculture | less than 1 year | strongly agree | strongly agree | strongly agree | strongly agree | strongly agree | strongly agree | strongly agree | strongly agree |
| male | 26-33 years | Education level below 10 | Trade | less than 1 year | strongly agree | strongly agree | strongly agree | strongly agree | strongly agree | strongly agree | strongly agree | strongly agree |
| male | 34-41 years | Education level below 10 | urban agriculture | above 6 years | strongly agree | strongly agree | strongly agree | strongly agree | strongly agree | strongly agree | strongly agree | strongly agree |
| male | 34-41 years | Education level below 10 | urban agriculture | 1-3 years | strongly agree | strongly agree | strongly agree | strongly agree | strongly agree | strongly agree | strongly agree | strongly agree |
| male | 34-41 years | Education level below 10 | Trade | less than 1 year | strongly agree | strongly agree | strongly agree | strongly agree | strongly agree | strongly agree | strongly agree | strongly agree |
| male | 18-25 years | Education level below 10 | urban agriculture | above 6 years | strongly agree | strongly agree | strongly agree | strongly agree | strongly agree | strongly agree | strongly agree | strongly agree |
| male | 26-33 years | Education level below 10 | urban agriculture | less than 1 year | strongly agree | strongly agree | strongly agree | strongly agree | strongly agree | strongly agree | strongly agree | strongly agree |
| male | 26-33 years | Education level below 10 | urban agriculture | above 6 years | strongly agree | strongly agree | strongly agree | strongly agree | strongly agree | strongly agree | strongly agree | strongly agree |
| male | 26-33 years | Education level below 10 | Trade | less than 1 year | strongly agree | strongly agree | strongly agree | strongly agree | strongly agree | strongly agree | strongly agree | strongly agree |
| male | above 41 | Education level below 10 | service | above 6 years | strongly agree | strongly agree | strongly agree | strongly agree | strongly agree | strongly agree | strongly agree | strongly agree |
| male | above 41 | Education level below 10 | Trade | above 6 years | strongly agree | strongly agree | strongly agree | strongly agree | strongly agree | strongly agree | strongly agree | strongly agree |
| male | 18-25 years | Education level below 10 | service | less than 1 year | strongly agree | strongly agree | strongly agree | strongly agree | strongly agree | strongly agree | strongly agree | strongly agree |
| male | 26-33 years | finished Ethiopian Secondary school 10+3 | Trade | less than 1 year | strongly agree | strongly agree | strongly agree | strongly agree | strongly agree | strongly agree | strongly agree | strongly agree |
| male | 26-33 years | finished Ethiopian Secondary school 10+3 | Trade | above 6 years | strongly agree | strongly agree | strongly agree | strongly agree | strongly agree | strongly agree | strongly agree | strongly agree |
| male | 34-41 years | Education level below 10 | Trade | above 6 years | strongly agree | strongly agree | strongly agree | strongly agree | strongly agree | strongly agree | strongly agree | strongly agree |
| male | 34-41 years | Education level below 10 | Trade | above 6 years | strongly agree | strongly agree | strongly agree | strongly agree | strongly agree | strongly agree | strongly agree | strongly agree |
| male | 26-33 years | finished Ethiopian Secondary school 10+3 | service | above 6 years | strongly agree | strongly agree | strongly agree | strongly agree | strongly agree | strongly agree | strongly agree | strongly agree |
| female | 26-33 years | Education level below 10 | Trade | above 6 years | strongly agree | strongly agree | strongly agree | strongly agree | strongly agree | strongly agree | strongly agree | strongly agree |
| female | 26-33 years | Education level below 10 | Trade | above 6 years | strongly agree | strongly agree | strongly agree | strongly agree | strongly agree | strongly agree | strongly agree | strongly agree |
| female | 34-41 years | finished Ethiopian Secondary school 10+3 | service | above 6 years | strongly agree | strongly agree | strongly agree | strongly agree | strongly agree | strongly agree | strongly agree | strongly agree |
| female | 34-41 years | finished Ethiopian Secondary school 10+3 | Trade | 1-3 years | strongly agree | strongly agree | strongly agree | strongly agree | strongly agree | strongly agree | strongly agree | strongly agree |
| male | 34-41 years | finished Ethiopian Secondary school 10+3 | service | 1-3 years | strongly agree | strongly agree | strongly agree | strongly agree | strongly agree | strongly agree | strongly agree | strongly agree |
| female | 34-41 years | Education level below 10 | Trade | above 6 years | strongly agree | strongly agree | strongly agree | strongly agree | strongly agree | strongly agree | strongly agree | strongly agree |
| female | above 41 | finished Ethiopian Secondary school 10+3 | Trade | less than 1 year | strongly agree | strongly agree | strongly agree | strongly agree | strongly agree | strongly agree | strongly agree | strongly agree |
| male | above 41 | finished Ethiopian Secondary school 10+3 | service | above 6 years | strongly agree | strongly agree | strongly agree | strongly agree | strongly agree | strongly agree | strongly agree | strongly agree |
| male | above 41 | Education level below 10 | Trade | less than 1 year | strongly agree | strongly agree | strongly agree | strongly agree | strongly agree | strongly agree | strongly agree | strongly agree |
| male | 26-33 years | Education level below 10 | manufacturing | above 6 years | strongly agree | strongly agree | strongly agree | strongly agree | strongly agree | strongly agree | strongly agree | strongly agree |
| male | above 41 | finished Ethiopian Secondary school 10+3 | Trade | less than 1 year | strongly agree | strongly agree | strongly agree | strongly agree | strongly agree | strongly agree | strongly agree | strongly agree |
| male | above 41 | Education level below 10 | Trade | above 6 years | strongly agree | strongly agree | strongly agree | strongly agree | strongly agree | strongly agree | strongly agree | strongly agree |
| female | above 41 | BA degree | Trade | less than 1 year | strongly agree | strongly agree | strongly agree | strongly agree | strongly agree | strongly agree | strongly agree | strongly agree |
| female | above 41 | BA degree | service | above 6 years | strongly agree | strongly agree | strongly agree | strongly agree | strongly agree | strongly agree | strongly agree | strongly agree |
| female | 34-41 years | Education level below 10 | Trade | less than 1 year | strongly agree | strongly agree | strongly agree | strongly agree | strongly agree | strongly agree | strongly agree | strongly agree |
| female | above 41 | BA degree | Trade | above 6 years | strongly agree | strongly agree | strongly agree | strongly agree | strongly agree | strongly agree | strongly agree | strongly agree |
| female | above 41 | finished Ethiopian Secondary school 10+3 | Trade | less than 1 year | strongly agree | strongly agree | strongly agree | strongly agree | strongly agree | strongly agree | strongly agree | strongly agree |
| female | 18-25 years | BA degree | Trade | above 6 years | strongly agree | strongly agree | strongly agree | strongly agree | strongly agree | strongly agree | strongly agree | strongly agree |
| male | 18-25 years | Education level below 10 | service | less than 1 year | strongly agree | strongly agree | strongly agree | strongly agree | strongly agree | strongly agree | strongly agree | strongly agree |
| female | 26-33 years | Education level below 10 | Trade | above 6 years | strongly agree | strongly agree | strongly agree | strongly agree | strongly agree | strongly agree | strongly agree | strongly agree |
| female | 18-25 years | Earned a deploma from a recognized institution | service | less than 1 year | strongly agree | strongly agree | strongly agree | strongly agree | strongly agree | strongly agree | strongly agree | strongly agree |
| female | 18-25 years | Education level below 10 | Trade | above 6 years | strongly agree | strongly agree | strongly agree | strongly agree | strongly agree | strongly agree | strongly agree | strongly agree |
| female | 34-41 years | Education level below 10 | service | 1-3 years | strongly agree | strongly agree | strongly agree | strongly agree | strongly agree | strongly agree | strongly agree | strongly agree |
| female | 18-25 years | Earned a deploma from a recognized institution | Trade | 1-3 years | strongly agree | strongly agree | strongly agree | strongly agree | strongly agree | strongly agree | strongly agree | strongly agree |
| male | 18-25 years | Education level below 10 | service | above 6 years | strongly agree | strongly agree | strongly agree | strongly agree | strongly agree | strongly agree | strongly agree | strongly agree |
| female | 18-25 years | Education level below 10 | Trade | less than 1 year | strongly agree | strongly agree | strongly agree | strongly agree | strongly agree | strongly agree | strongly agree | strongly agree |
| male | 18-25 years | Education level below 10 | Trade | above 6 years | strongly agree | strongly agree | strongly agree | strongly agree | strongly agree | strongly agree | strongly agree | strongly agree |
| male | 18-25 years | Education level below 10 | Trade | less than 1 year | strongly agree | strongly agree | strongly agree | strongly agree | strongly agree | strongly agree | strongly agree | strongly agree |
| male | 18-25 years | Earned a deploma from a recognized institution | service | 4-6 years | strongly agree | strongly agree | strongly agree | strongly agree | strongly agree | strongly agree | strongly agree | strongly agree |
| male | 26-33 years | Education level below 10 | Trade | less than 1 year | strongly agree | strongly agree | strongly agree | strongly agree | strongly agree | strongly agree | strongly agree | strongly agree |
| male | 18-25 years | Earned a deploma from a recognized institution | service | 4-6 years | strongly agree | strongly agree | strongly agree | strongly agree | strongly agree | strongly agree | strongly agree | strongly agree |
| male | 18-25 years | Earned a deploma from a recognized institution | Trade | less than 1 year | strongly agree | strongly agree | strongly agree | strongly agree | strongly agree | strongly agree | strongly agree | strongly agree |
| male | 26-33 years | Education level below 10 | Trade | 4-6 years | strongly agree | strongly agree | strongly agree | strongly agree | strongly agree | strongly agree | strongly agree | strongly agree |
| male | 18-25 years | Education level below 10 | service | 1-3 years | strongly agree | strongly agree | strongly agree | strongly agree | strongly agree | strongly agree | strongly agree | strongly agree |
| female | 18-25 years | Education level below 10 | Trade | 4-6 years | strongly agree | strongly agree | strongly agree | strongly agree | strongly agree | strongly agree | strongly agree | strongly agree |
| male | 18-25 years | Earned a deploma from a recognized institution | service | 4-6 years | strongly agree | strongly agree | strongly agree | strongly agree | strongly agree | strongly agree | strongly agree | strongly agree |
| male | 34-41 years | Education level below 10 | service | 4-6 years | strongly agree | strongly agree | strongly agree | strongly agree | strongly agree | strongly agree | strongly agree | strongly agree |
| male | 18-25 years | Education level below 10 | Trade | 4-6 years | strongly agree | strongly agree | strongly agree | strongly agree | strongly agree | strongly agree | strongly agree | strongly agree |
| male | 18-25 years | Earned a deploma from a recognized institution | service | 4-6 years | strongly agree | strongly agree | strongly agree | strongly agree | strongly agree | strongly agree | strongly agree | strongly agree |
| male | 18-25 years | Education level below 10 | Trade | less than 1 year | strongly agree | strongly agree | strongly agree | strongly agree | strongly agree | strongly agree | strongly agree | strongly agree |
| male | 34-41 years | Education level below 10 | Trade | 4-6 years | strongly agree | strongly agree | strongly agree | strongly agree | strongly agree | strongly agree | strongly agree | strongly agree |
| male | above 41 | finished Ethiopian Secondary school 10+3 | Trade | less than 1 year | strongly agree | strongly agree | strongly agree | strongly agree | strongly agree | strongly agree | strongly agree | strongly agree |
| male | above 41 | Education level below 10 | service | 4-6 years | strongly agree | strongly agree | strongly agree | strongly agree | strongly agree | strongly agree | strongly agree | strongly agree |
| male | 26-33 years | Education level below 10 | Trade | less than 1 year | strongly agree | strongly agree | strongly agree | strongly agree | strongly agree | strongly agree | strongly agree | strongly agree |
| male | 26-33 years | finished Ethiopian Secondary school 10+3 | Trade | 1-3 years | strongly agree | strongly agree | strongly agree | strongly agree | strongly agree | strongly agree | strongly agree | strongly agree |
| male | 18-25 years | Education level below 10 | Trade | 1-3 years | strongly agree | strongly agree | strongly agree | strongly agree | strongly agree | strongly agree | strongly agree | strongly agree |
| male | 26-33 years | Education level below 10 | service | 1-3 years | strongly agree | strongly agree | strongly agree | strongly agree | strongly agree | strongly agree | strongly agree | strongly agree |
| male | 34-41 years | finished Ethiopian Secondary school 10+3 | construction | 4-6 years | strongly agree | strongly agree | strongly agree | strongly agree | strongly agree | strongly agree | strongly agree | strongly agree |
| male | above 41 | finished Ethiopian Secondary school 10+3 | Trade | 4-6 years | strongly agree | strongly agree | strongly agree | strongly agree | strongly agree | strongly agree | strongly agree | strongly agree |
| male | above 41 | finished Ethiopian Secondary school 10+3 | construction | less than 1 year | strongly agree | strongly agree | strongly agree | strongly agree | strongly agree | strongly agree | strongly agree | strongly agree |
| male | above 41 | finished Ethiopian Secondary school 10+3 | Trade | above 6 years | strongly agree | strongly agree | strongly agree | strongly agree | strongly agree | strongly agree | strongly agree | strongly agree |
| male | 18-25 years | Education level below 10 | Trade | less than 1 year | strongly agree | strongly agree | strongly agree | strongly agree | strongly agree | strongly agree | strongly agree | strongly agree |
| male | 18-25 years | finished Ethiopian Secondary school 10+3 | construction | above 6 years | strongly agree | strongly agree | strongly agree | strongly agree | strongly agree | strongly agree | strongly agree | strongly agree |
| male | 18-25 years | Education level below 10 | Trade | less than 1 year | strongly agree | strongly agree | strongly agree | strongly agree | strongly agree | strongly agree | strongly agree | strongly agree |
| male | 34-41 years | Education level below 10 | construction | above 6 years | strongly agree | strongly agree | strongly agree | strongly agree | strongly agree | strongly agree | strongly agree | strongly agree |
| male | 34-41 years | Earned a deploma from a recognized institution | Trade | less than 1 year | strongly agree | strongly agree | strongly agree | strongly agree | strongly agree | strongly agree | strongly agree | strongly agree |
| female | 34-41 years | Earned a deploma from a recognized institution | Trade | 4-6 years | strongly agree | strongly agree | strongly agree | strongly agree | strongly agree | strongly agree | strongly agree | strongly agree |
| female | 34-41 years | Earned a deploma from a recognized institution | construction | less than 1 year | strongly agree | strongly agree | strongly agree | strongly agree | strongly agree | strongly agree | strongly agree | strongly agree |
| female | 34-41 years | Earned a deploma from a recognized institution | Trade | above 6 years | strongly agree | strongly agree | strongly agree | strongly agree | strongly agree | strongly agree | strongly agree | strongly agree |
| female | 34-41 years | Earned a deploma from a recognized institution | Trade | less than 1 year | strongly agree | strongly agree | strongly agree | strongly agree | strongly agree | strongly agree | strongly agree | strongly agree |
| female | 34-41 years | Earned a deploma from a recognized institution | service | above 6 years | strongly agree | strongly agree | strongly agree | strongly agree | strongly agree | strongly agree | strongly agree | strongly agree |
| female | 34-41 years | Education level below 10 | Trade | 1-3 years | strongly agree | strongly agree | strongly agree | strongly agree | strongly agree | strongly agree | strongly agree | strongly agree |
| female | 34-41 years | Education level below 10 | service | above 6 years | strongly agree | strongly agree | strongly agree | strongly agree | strongly agree | strongly agree | strongly agree | strongly agree |
| female | 34-41 years | Earned a deploma from a recognized institution | Trade | above 6 years | strongly agree | strongly agree | strongly agree | strongly agree | strongly agree | strongly agree | strongly agree | strongly agree |
| female | 18-25 years | Education level below 10 | urban agriculture | less than 1 year | strongly agree | strongly agree | strongly agree | strongly agree | strongly agree | strongly agree | strongly agree | strongly agree |
| female | 34-41 years | Education level below 10 | Trade | 4-6 years | strongly agree | strongly agree | strongly agree | strongly agree | strongly agree | strongly agree | strongly agree | strongly agree |
| female | 34-41 years | Earned a deploma from a recognized institution | Trade | less than 1 year | strongly agree | strongly agree | strongly agree | strongly agree | strongly agree | strongly agree | strongly agree | strongly agree |
| female | 34-41 years | Education level below 10 | urban agriculture | 4-6 years | strongly agree | strongly agree | strongly agree | strongly agree | strongly agree | strongly agree | strongly agree | strongly agree |
| female | above 41 | Education level below 10 | urban agriculture | less than 1 year | strongly agree | strongly agree | strongly agree | strongly agree | strongly agree | strongly agree | strongly agree | strongly agree |
| female | 34-41 years | Earned a deploma from a recognized institution | Trade | 4-6 years | strongly agree | strongly agree | strongly agree | strongly agree | strongly agree | strongly agree | strongly agree | strongly agree |
| female | 34-41 years | Education level below 10 | urban agriculture | less than 1 year | strongly agree | strongly agree | strongly agree | strongly agree | strongly agree | strongly agree | strongly agree | strongly agree |
| male | 26-33 years | Education level below 10 | urban agriculture | 4-6 years | strongly agree | strongly agree | strongly agree | strongly agree | strongly agree | strongly agree | strongly agree | strongly agree |
| male | 26-33 years | Earned a deploma from a recognized institution | Trade | 1-3 years | strongly agree | strongly agree | strongly agree | strongly agree | strongly agree | strongly agree | strongly agree | strongly agree |
| male | 18-25 years | Earned a deploma from a recognized institution | Trade | 4-6 years | strongly agree | strongly agree | strongly agree | strongly agree | strongly agree | strongly agree | strongly agree | strongly agree |
| female | 26-33 years | Earned a deploma from a recognized institution | Trade | less than 1 year | strongly agree | strongly agree | strongly agree | strongly agree | strongly agree | strongly agree | strongly agree | strongly agree |
| male | 34-41 years | Education level below 10 | Trade | 4-6 years | strongly agree | strongly agree | strongly agree | strongly agree | strongly agree | strongly agree | strongly agree | strongly agree |
| male | 18-25 years | Education level below 10 | Trade | less than 1 year | strongly agree | strongly agree | strongly agree | strongly agree | strongly agree | strongly agree | strongly agree | strongly agree |
| male | 18-25 years | Education level below 10 | Trade | 1-3 years | strongly agree | strongly agree | strongly agree | strongly agree | strongly agree | strongly agree | strongly agree | strongly agree |
| male | 18-25 years | Education level below 10 | Trade | 1-3 years | strongly agree | strongly agree | strongly agree | strongly agree | strongly agree | strongly agree | strongly agree | strongly agree |
| male | 18-25 years | Earned a deploma from a recognized institution | construction | 4-6 years | strongly agree | strongly agree | strongly agree | strongly agree | strongly agree | strongly agree | strongly agree | strongly agree |
| male | 26-33 years | Earned a deploma from a recognized institution | Trade | 1-3 years | strongly agree | strongly agree | strongly agree | strongly agree | strongly agree | strongly agree | strongly agree | strongly agree |
| male | 18-25 years | Earned a deploma from a recognized institution | construction | 4-6 years | strongly agree | strongly agree | strongly agree | strongly agree | strongly agree | strongly agree | strongly agree | strongly agree |
| male | 34-41 years | Earned a deploma from a recognized institution | construction | 4-6 years | strongly agree | strongly agree | strongly agree | strongly agree | strongly agree | strongly agree | strongly agree | strongly agree |
| male | 18-25 years | BA degree | construction | 4-6 years | strongly agree | strongly agree | strongly agree | strongly agree | strongly agree | strongly agree | strongly agree | strongly agree |
| female | 18-25 years | BA degree | Trade | above 6 years | strongly agree | strongly agree | strongly agree | strongly agree | strongly agree | strongly agree | strongly agree | strongly agree |
| male | 26-33 years | Earned a deploma from a recognized institution | Trade | above 6 years | strongly agree | strongly agree | strongly agree | strongly agree | strongly agree | strongly agree | strongly agree | strongly agree |
| male | 18-25 years | BA degree | Trade | 1-3 years | strongly agree | strongly agree | strongly agree | strongly agree | strongly agree | strongly agree | strongly agree | strongly agree |
| male | 18-25 years | Earned a deploma from a recognized institution | Trade | above 6 years | strongly agree | strongly agree | strongly agree | strongly agree | strongly agree | strongly agree | strongly agree | strongly agree |
| male | above 41 | Earned a deploma from a recognized institution | Trade | 1-3 years | strongly agree | strongly agree | strongly agree | strongly agree | strongly agree | strongly agree | strongly agree | strongly agree |
| male | 18-25 years | BA degree | manufacturing | 1-3 years | strongly agree | strongly agree | strongly agree | strongly agree | strongly agree | strongly agree | strongly agree | strongly agree |
| male | 18-25 years | Earned a deploma from a recognized institution | Trade | 1-3 years | strongly agree | strongly agree | strongly agree | strongly agree | strongly agree | strongly agree | strongly agree | strongly agree |
| female | 26-33 years | BA degree | Trade | 4-6 years | strongly agree | strongly agree | strongly agree | strongly agree | strongly agree | strongly agree | strongly agree | strongly agree |
| female | 18-25 years | BA degree | Trade | above 6 years | strongly agree | strongly agree | strongly agree | strongly agree | strongly agree | strongly agree | strongly agree | strongly agree |
| female | above 41 | Education level below 10 | service | 1-3 years | strongly agree | strongly agree | strongly agree | strongly agree | strongly agree | strongly agree | strongly agree | strongly agree |
| male | 18-25 years | BA degree | Trade | above 6 years | strongly agree | strongly agree | strongly agree | strongly agree | strongly agree | strongly agree | strongly agree | strongly agree |
| male | 18-25 years | Education level below 10 | service | 1-3 years | strongly agree | strongly agree | strongly agree | strongly agree | strongly agree | strongly agree | strongly agree | strongly agree |
| male | 26-33 years | BA degree | Trade | above 6 years | strongly agree | strongly agree | strongly agree | strongly agree | strongly agree | strongly agree | strongly agree | strongly agree |
| male | 26-33 years | Education level below 10 | service | 1-3 years | strongly agree | strongly agree | strongly agree | strongly agree | strongly agree | strongly agree | strongly agree | strongly agree |
| male | 26-33 years | Education level below 10 | Trade | above 6 years | strongly agree | strongly agree | strongly agree | strongly agree | strongly agree | strongly agree | strongly agree | strongly agree |
| male | 34-41 years | Education level below 10 | Trade | 1-3 years | strongly agree | strongly agree | strongly agree | strongly agree | strongly agree | strongly agree | strongly agree | strongly agree |
| male | 26-33 years | Education level below 10 | service | above 6 years | strongly agree | strongly agree | strongly agree | strongly agree | strongly agree | strongly agree | strongly agree | strongly agree |
| male | 34-41 years | BA degree | service | 1-3 years | strongly agree | strongly agree | strongly agree | strongly agree | strongly agree | strongly agree | strongly agree | strongly agree |
| male | 26-33 years | Education level below 10 | Trade | 1-3 years | strongly agree | strongly agree | strongly agree | strongly agree | strongly agree | strongly agree | strongly agree | strongly agree |
| male | 26-33 years | BA degree | Trade | above 6 years | strongly agree | strongly agree | strongly agree | strongly agree | strongly agree | strongly agree | strongly agree | strongly agree |
| male | 26-33 years | Education level below 10 | service | above 6 years | strongly agree | strongly agree | strongly agree | strongly agree | strongly agree | strongly agree | strongly agree | strongly agree |
| female | 26-33 years | BA degree | Trade | less than 1 year | strongly agree | strongly agree | strongly agree | strongly agree | strongly agree | strongly agree | strongly agree | strongly agree |
| female | 26-33 years | BA degree | service | 1-3 years | strongly agree | strongly agree | strongly agree | strongly agree | strongly agree | strongly agree | strongly agree | strongly agree |
| female | above 41 | finished Ethiopian Secondary school 10+3 | Trade | 1-3 years | strongly agree | strongly agree | strongly agree | strongly agree | strongly agree | strongly agree | strongly agree | strongly agree |
| female | above 41 | Education level below 10 | service | 1-3 years | strongly agree | strongly agree | strongly agree | strongly agree | strongly agree | strongly agree | strongly agree | strongly agree |
| female | 34-41 years | finished Ethiopian Secondary school 10+3 | Trade | 4-6 years | strongly agree | strongly agree | strongly agree | strongly agree | strongly agree | strongly agree | strongly agree | strongly agree |
| female | 34-41 years | finished Ethiopian Secondary school 10+3 | Trade | less than 1 year | strongly agree | strongly agree | strongly agree | strongly agree | strongly agree | strongly agree | strongly agree | strongly agree |
| female | 34-41 years | Education level below 10 | Trade | 4-6 years | strongly agree | strongly agree | strongly agree | strongly agree | strongly agree | strongly agree | strongly agree | strongly agree |
| female | 26-33 years | Education level below 10 | Trade | less than 1 year | strongly agree | strongly agree | strongly agree | strongly agree | strongly agree | strongly agree | strongly agree | strongly agree |
| female | 26-33 years | Education level below 10 | service | 4-6 years | strongly agree | strongly agree | strongly agree | strongly agree | strongly agree | strongly agree | strongly agree | strongly agree |
| female | 26-33 years | Education level below 10 | Trade | less than 1 year | strongly agree | strongly agree | strongly agree | strongly agree | strongly agree | strongly agree | strongly agree | strongly agree |
| female | 34-41 years | finished Ethiopian Secondary school 10+3 | Trade | above 6 years | strongly agree | strongly agree | strongly agree | strongly agree | strongly agree | strongly agree | strongly agree | strongly agree |
| female | 26-33 years | Education level below 10 | Trade | less than 1 year | strongly agree | strongly agree | strongly agree | strongly agree | strongly agree | strongly agree | strongly agree | strongly agree |
| female | 26-33 years | finished Ethiopian Secondary school 10+3 | service | above 6 years | strongly agree | strongly agree | strongly agree | strongly agree | strongly agree | strongly agree | strongly agree | strongly agree |
| female | 26-33 years | Education level below 10 | Trade | less than 1 year | strongly disagree | strongly disagree | strongly agree | strongly disagree | strongly agree | strongly agree | strongly agree | strongly agree |
| male | 26-33 years | finished Ethiopian Secondary school 10+3 | Trade | above 6 years | strongly agree | strongly agree | strongly agree | strongly disagree | strongly agree | strongly agree | strongly agree | strongly agree |
| male | 26-33 years | Education level below 10 | Trade | less than 1 year | strongly agree | strongly agree | strongly agree | strongly disagree | strongly agree | strongly agree | strongly agree | strongly agree |
| male | 26-33 years | finished Ethiopian Secondary school 10+3 | Trade | 1-3 years | strongly agree | strongly agree | strongly agree | strongly disagree | strongly agree | strongly agree | strongly agree | strongly agree |
| male | 34-41 years | Education level below 10 | Trade | 1-3 years | strongly agree | strongly agree | strongly agree | strongly disagree | strongly agree | strongly agree | strongly agree | strongly agree |
| male | 34-41 years | finished Ethiopian Secondary school 10+3 | Trade | above 6 years | strongly agree | strongly agree | strongly agree | strongly disagree | strongly agree | strongly agree | strongly agree | strongly agree |
| male | 34-41 years | finished Ethiopian Secondary school 10+3 | Trade | less than 1 year | strongly agree | strongly agree | strongly agree | strongly disagree | strongly agree | strongly agree | strongly agree | strongly agree |
| male | 34-41 years | Education level below 10 | service | above 6 years | strongly agree | strongly agree | strongly agree | strongly disagree | strongly agree | strongly agree | strongly agree | strongly agree |
| male | 34-41 years | Education level below 10 | Trade | less than 1 year | strongly agree | strongly agree | strongly agree | strongly disagree | strongly agree | strongly agree | strongly agree | strongly agree |
| male | 34-41 years | Education level below 10 | service | above 6 years | strongly agree | strongly agree | strongly agree | strongly disagree | strongly agree | strongly agree | strongly agree | strongly agree |
| male | 34-41 years | Education level below 10 | service | less than 1 year | strongly agree | strongly agree | strongly agree | strongly disagree | strongly agree | strongly agree | strongly agree | strongly agree |
| male | 34-41 years | Education level below 10 | service | less than 1 year | strongly disagree | strongly agree | strongly agree | strongly disagree | strongly agree | strongly agree | strongly agree | strongly agree |
| male | 34-41 years | Education level below 10 | Trade | above 6 years | strongly disagree | strongly agree | strongly agree | strongly disagree | strongly agree | strongly agree | strongly agree | strongly agree |
| male | 34-41 years | Education level below 10 | Trade | less than 1 year | strongly disagree | strongly agree | strongly agree | strongly disagree | strongly agree | strongly agree | strongly agree | strongly agree |
| male | above 41 | finished Ethiopian Secondary school 10+3 | Trade | above 6 years | strongly disagree | strongly agree | strongly agree | disagree | strongly agree | strongly agree | strongly agree | strongly agree |
| male | above 41 | finished Ethiopian Secondary school 10+3 | Trade | less than 1 year | strongly disagree | strongly agree | strongly agree | strongly disagree | strongly agree | strongly agree | strongly agree | strongly agree |
| male | above 41 | finished Ethiopian Secondary school 10+3 | Trade | above 6 years | strongly disagree | strongly disagree | strongly agree | strongly disagree | strongly agree | strongly agree | strongly agree | strongly agree |
| male | above 41 | finished Ethiopian Secondary school 10+3 | Trade | less than 1 year | strongly disagree | strongly disagree | strongly agree | strongly disagree | strongly agree | strongly agree | strongly agree | strongly agree |
| male | above 41 | finished Ethiopian Secondary school 10+3 | Trade | above 6 years | strongly disagree | disagree | strongly agree | strongly disagree | strongly agree | strongly agree | strongly agree | strongly agree |
| male | above 41 | finished Ethiopian Secondary school 10+3 | Trade | less than 1 year | disagree | strongly disagree | strongly agree | Neutral | strongly agree | strongly agree | strongly agree | strongly agree |
| male | 26-33 years | BA degree | Trade | above 6 years | disagree | disagree | strongly agree | strongly disagree | strongly agree | strongly agree | strongly agree | strongly agree |
| male | 18-25 years | finished Ethiopian Secondary school 10+3 | Trade | less than 1 year | disagree | strongly disagree | strongly agree | disagree | strongly agree | strongly agree | strongly agree | strongly agree |
| male | 18-25 years | finished Ethiopian Secondary school 10+3 | Trade | less than 1 year | disagree | disagree | strongly agree | strongly disagree | strongly agree | strongly agree | strongly agree | strongly agree |
| male | 18-25 years | finished Ethiopian Secondary school 10+3 | Trade | less than 1 year | disagree | strongly disagree | strongly agree | strongly disagree | strongly agree | strongly agree | strongly agree | strongly agree |
| male | 18-25 years | Earned a deploma from a recognized institution | Trade | less than 1 year | disagree | disagree | strongly agree | strongly disagree | strongly agree | strongly agree | strongly agree | strongly agree |
| male | 34-41 years | Earned a deploma from a recognized institution | service | 1-3 years | disagree | strongly disagree | strongly agree | strongly disagree | strongly agree | strongly agree | strongly agree | strongly agree |
| male | 26-33 years | BA degree | Trade | above 6 years | disagree | strongly disagree | strongly agree | disagree | strongly agree | strongly agree | strongly agree | strongly agree |
| male | 26-33 years | Earned a deploma from a recognized institution | Trade | less than 1 year | disagree | disagree | strongly agree | strongly disagree | strongly agree | strongly agree | strongly agree | strongly agree |
| male | 34-41 years | BA degree | Trade | above 6 years | disagree | strongly disagree | strongly agree | strongly disagree | strongly agree | strongly agree | strongly agree | strongly agree |
| male | 34-41 years | finished Ethiopian Secondary school 10+3 | Trade | less than 1 year | disagree | strongly disagree | strongly agree | strongly disagree | strongly agree | strongly agree | strongly agree | strongly agree |
| female | 18-25 years | finished Ethiopian Secondary school 10+3 | Trade | less than 1 year | disagree | disagree | strongly agree | Neutral | strongly agree | strongly agree | strongly agree | strongly agree |
| female | 18-25 years | finished Ethiopian Secondary school 10+3 | Trade | less than 1 year | disagree | disagree | disagree | strongly disagree | disagree | disagree | disagree | disagree |
| female | 34-41 years | Earned a deploma from a recognized institution | Trade | less than 1 year | disagree | strongly disagree | strongly disagree | strongly disagree | strongly disagree | strongly disagree | strongly disagree | strongly disagree |
| male | 34-41 years | Earned a deploma from a recognized institution | Trade | above 6 years | strongly disagree | disagree | disagree | strongly disagree | disagree | disagree | disagree | disagree |
| male | 34-41 years | finished Ethiopian Secondary school 10+3 | Trade | less than 1 year | disagree | strongly disagree | disagree | strongly disagree | disagree | disagree | disagree | disagree |
| female | 34-41 years | Earned a deploma from a recognized institution | Trade | above 6 years | strongly disagree | disagree | disagree | strongly disagree | disagree | disagree | disagree | disagree |
| female | 34-41 years | Earned a deploma from a recognized institution | Trade | less than 1 year | strongly disagree | disagree | disagree | strongly disagree | disagree | disagree | disagree | disagree |
| male | 34-41 years | finished Ethiopian Secondary school 10+3 | Trade | above 6 years | Neutral | disagree | strongly disagree | disagree | strongly disagree | strongly disagree | strongly disagree | strongly disagree |
| male | 34-41 years | finished Ethiopian Secondary school 10+3 | service | less than 1 year | strongly disagree | disagree | disagree | strongly disagree | disagree | disagree | disagree | disagree |
| female | 34-41 years | Earned a deploma from a recognized institution | Trade | less than 1 year | strongly disagree | disagree | strongly disagree | strongly disagree | strongly disagree | strongly disagree | strongly disagree | strongly disagree |
| male | 34-41 years | finished Ethiopian Secondary school 10+3 | Trade | 1-3 years | disagree | disagree | Neutral | Neutral | Neutral | Neutral | Neutral | Neutral |
| female | 34-41 years | finished Ethiopian Secondary school 10+3 | Trade | above 6 years | Neutral | disagree | disagree | strongly disagree | disagree | disagree | disagree | disagree |
| male | above 41 | Earned a deploma from a recognized institution | service | less than 1 year | Neutral | strongly disagree | disagree | strongly disagree | disagree | disagree | disagree | disagree |
| female | above 41 | finished Ethiopian Secondary school 10+3 | Trade | above 6 years | Neutral | disagree | disagree | strongly disagree | disagree | disagree | disagree | disagree |
| female | above 41 | finished Ethiopian Secondary school 10+3 | Trade | less than 1 year | Neutral | strongly disagree | disagree | disagree | disagree | disagree | disagree | disagree |
| male | above 41 | finished Ethiopian Secondary school 10+3 | Trade | above 6 years | Neutral | strongly disagree | disagree | Neutral | disagree | disagree | disagree | disagree |
| female | 18-25 years | Earned a deploma from a recognized institution | Trade | less than 1 year | Neutral | strongly disagree | disagree | strongly disagree | disagree | disagree | disagree | disagree |
| male | 18-25 years | finished Ethiopian Secondary school 10+3 | Trade | less than 1 year | Neutral | strongly disagree | disagree | strongly disagree | disagree | disagree | disagree | disagree |
| male | 18-25 years | finished Ethiopian Secondary school 10+3 | Trade | above 6 years | Neutral | strongly disagree | strongly disagree | strongly disagree | strongly disagree | strongly disagree | strongly disagree | strongly disagree |
| male | 26-33 years | finished Ethiopian Secondary school 10+3 | Trade | less than 1 year | Neutral | disagree | disagree | strongly disagree | disagree | disagree | disagree | disagree |
| male | 34-41 years | finished Ethiopian Secondary school 10+3 | service | less than 1 year | Neutral | disagree | disagree | strongly disagree | disagree | disagree | disagree | disagree |
| male | 34-41 years | finished Ethiopian Secondary school 10+3 | Trade | above 6 years | Neutral | strongly disagree | strongly disagree | disagree | strongly disagree | strongly disagree | strongly disagree | strongly disagree |
| male | 18-25 years | Education level below 10 | Trade | less than 1 year | Neutral | disagree | strongly disagree | strongly disagree | strongly disagree | strongly disagree | strongly disagree | strongly disagree |
| male | 34-41 years | Education level below 10 | service | 1-3 years | Neutral | strongly disagree | Neutral | strongly disagree | Neutral | Neutral | Neutral | Neutral |
| male | 34-41 years | Education level below 10 | service | 1-3 years | Neutral | disagree | disagree | Neutral | disagree | disagree | disagree | disagree |
| female | 26-33 years | finished Ethiopian Secondary school 10+3 | Trade | above 6 years | Neutral | strongly disagree | strongly disagree | strongly disagree | strongly disagree | strongly disagree | strongly disagree | strongly disagree |
| female | 26-33 years | Education level below 10 | Trade | less than 1 year | Neutral | disagree | disagree | strongly disagree | disagree | disagree | disagree | disagree |
| female | 26-33 years | Education level below 10 | Trade | above 6 years | Neutral | disagree | strongly disagree | strongly disagree | strongly disagree | strongly disagree | strongly disagree | strongly disagree |
| female | 18-25 years | finished Ethiopian Secondary school 10+3 | Trade | less than 1 year | Neutral | disagree | disagree | strongly disagree | disagree | disagree | disagree | disagree |
| female | 18-25 years | finished Ethiopian Secondary school 10+3 | Trade | above 6 years | Neutral | disagree | strongly disagree | disagree | strongly disagree | strongly disagree | strongly disagree | strongly disagree |
| male | 34-41 years | Education level below 10 | service | less than 1 year | Neutral | disagree | disagree | Neutral | disagree | disagree | disagree | disagree |
| male | 34-41 years | Education level below 10 | service | above 6 years | Neutral | disagree | disagree | strongly disagree | disagree | disagree | disagree | disagree |
| male | 26-33 years | finished Ethiopian Secondary school 10+3 | service | less than 1 year | Neutral | disagree | disagree | strongly disagree | disagree | disagree | disagree | disagree |
| female | 26-33 years | Education level below 10 | Trade | above 6 years | Neutral | strongly disagree | disagree | strongly disagree | disagree | disagree | disagree | disagree |
| male | 26-33 years | Education level below 10 | Trade | less than 1 year | Neutral | strongly disagree | disagree | strongly disagree | disagree | disagree | disagree | disagree |
| female | 26-33 years | finished Ethiopian Secondary school 10+3 | Trade | above 6 years | Neutral | disagree | Neutral | strongly disagree | Neutral | Neutral | Neutral | Neutral |
| female | 26-33 years | Education level below 10 | Trade | less than 1 year | Neutral | disagree | disagree | disagree | disagree | disagree | disagree | disagree |
| female | 26-33 years | Education level below 10 | urban agriculture | above 6 years | disagree | disagree | Neutral | Neutral | Neutral | Neutral | Neutral | Neutral |
| male | 26-33 years | Education level below 10 | Trade | 1-3 years | disagree | disagree | disagree | strongly disagree | disagree | disagree | disagree | disagree |
| male | 26-33 years | Education level below 10 | urban agriculture | above 6 years | disagree | strongly disagree | Neutral | strongly disagree | Neutral | Neutral | Neutral | Neutral |
| female | 26-33 years | finished Ethiopian Secondary school 10+3 | urban agriculture | less than 1 year | disagree | disagree | disagree | strongly disagree | disagree | disagree | disagree | disagree |
| female | 26-33 years | Education level below 10 | Trade | above 6 years | disagree | strongly disagree | disagree | disagree | disagree | disagree | disagree | disagree |
| female | 26-33 years | Education level below 10 | urban agriculture | less than 1 year | strongly disagree | disagree | disagree | strongly disagree | disagree | disagree | disagree | disagree |
| female | 26-33 years | finished Ethiopian Secondary school 10+3 | urban agriculture | less than 1 year | strongly disagree | strongly disagree | disagree | Neutral | disagree | disagree | disagree | disagree |
| female | 26-33 years | Education level below 10 | urban agriculture | less than 1 year | strongly disagree | disagree | disagree | strongly disagree | disagree | disagree | disagree | disagree |
| female | 26-33 years | finished Ethiopian Secondary school 10+3 | Trade | less than 1 year | strongly disagree | disagree | Neutral | strongly disagree | Neutral | Neutral | Neutral | Neutral |
| male | 26-33 years | Education level below 10 | urban agriculture | above 6 years | strongly disagree | disagree | disagree | strongly disagree | disagree | disagree | disagree | disagree |
| male | 26-33 years | Education level below 10 | urban agriculture | less than 1 year | strongly disagree | strongly disagree | Neutral | strongly disagree | Neutral | Neutral | Neutral | Neutral |
| female | above 41 | Education level below 10 | urban agriculture | above 6 years | strongly disagree | disagree | disagree | strongly disagree | disagree | disagree | disagree | disagree |
| male | above 41 | Earned a deploma from a recognized institution | Trade | 1-3 years | strongly disagree | strongly disagree | Neutral | strongly disagree | Neutral | Neutral | Neutral | Neutral |
| female | above 41 | Education level below 10 | Trade | less than 1 year | strongly disagree | disagree | disagree | disagree | disagree | disagree | disagree | disagree |
| male | above 41 | finished Ethiopian Secondary school 10+3 | Trade | above 6 years | agree | strongly disagree | Neutral | strongly disagree | Neutral | Neutral | Neutral | Neutral |
| male | above 41 | Education level below 10 | Trade | less than 1 year | strongly disagree | disagree | Neutral | strongly disagree | Neutral | Neutral | Neutral | Neutral |
| male | 34-41 years | Education level below 10 | urban agriculture | less than 1 year | agree | strongly disagree | disagree | strongly disagree | disagree | disagree | disagree | disagree |
| male | above 41 | Education level below 10 | urban agriculture | less than 1 year | agree | disagree | Neutral | Neutral | Neutral | Neutral | Neutral | Neutral |
| male | above 41 | Earned a deploma from a recognized institution | Trade | less than 1 year | agree | disagree | disagree | strongly disagree | disagree | disagree | disagree | disagree |
| female | 18-25 years | Education level below 10 | urban agriculture | 1-3 years | agree | strongly disagree | Neutral | strongly disagree | Neutral | Neutral | Neutral | Neutral |
| male | 18-25 years | Education level below 10 | urban agriculture | less than 1 year | agree | disagree | disagree | strongly disagree | disagree | disagree | disagree | disagree |
| female | above 41 | Earned a deploma from a recognized institution | Trade | above 6 years | agree | disagree | disagree | strongly disagree | disagree | disagree | disagree | disagree |
| female | 34-41 years | Education level below 10 | urban agriculture | less than 1 year | agree | strongly disagree | disagree | strongly disagree | disagree | disagree | disagree | disagree |
| male | 26-33 years | Education level below 10 | urban agriculture | 1-3 years | agree | disagree | disagree | disagree | disagree | disagree | disagree | disagree |
| male | 18-25 years | Education level below 10 | urban agriculture | less than 1 year | agree | disagree | disagree | strongly disagree | disagree | disagree | disagree | disagree |
| male | 26-33 years | Education level below 10 | urban agriculture | above 6 years | strongly agree | strongly disagree | disagree | Neutral | disagree | disagree | disagree | disagree |
| male | above 41 | Earned a deploma from a recognized institution | Trade | less than 1 year | strongly agree | disagree | disagree | strongly disagree | disagree | disagree | disagree | disagree |
| male | 26-33 years | Education level below 10 | Trade | above 6 years | strongly agree | disagree | disagree | strongly disagree | disagree | disagree | disagree | disagree |
| male | 34-41 years | Earned a deploma from a recognized institution | urban agriculture | less than 1 year | strongly agree | disagree | disagree | strongly disagree | disagree | disagree | disagree | disagree |
| male | 26-33 years | Education level below 10 | urban agriculture | above 6 years | strongly agree | strongly disagree | disagree | strongly disagree | disagree | disagree | disagree | disagree |
| male | above 41 | Education level below 10 | urban agriculture | less than 1 year | strongly agree | disagree | disagree | strongly disagree | disagree | disagree | disagree | disagree |
| male | 26-33 years | Earned a deploma from a recognized institution | Trade | above 6 years | agree | disagree | disagree | strongly disagree | disagree | disagree | disagree | disagree |
| female | 26-33 years | Education level below 10 | Trade | less than 1 year | agree | disagree | Neutral | disagree | Neutral | Neutral | Neutral | Neutral |
| female | 26-33 years | Education level below 10 | urban agriculture | less than 1 year | agree | strongly disagree | disagree | strongly disagree | disagree | disagree | disagree | disagree |
| female | 34-41 years | Earned a deploma from a recognized institution | Trade | 4-6 years | agree | disagree | disagree | strongly disagree | disagree | disagree | disagree | disagree |
| male | above 41 | Education level below 10 | urban agriculture | less than 1 year | agree | disagree | disagree | Neutral | disagree | disagree | disagree | disagree |
| male | above 41 | Education level below 10 | urban agriculture | 4-6 years | strongly agree | disagree | disagree | strongly disagree | disagree | disagree | disagree | disagree |
| male | 34-41 years | Education level below 10 | urban agriculture | less than 1 year | strongly agree | disagree | disagree | strongly disagree | disagree | disagree | disagree | disagree |
| male | above 41 | finished Ethiopian Secondary school 10+3 | urban agriculture | 4-6 years | strongly agree | disagree | Neutral | strongly disagree | Neutral | Neutral | Neutral | Neutral |
| male | 18-25 years | Education level below 10 | Trade | less than 1 year | strongly disagree | disagree | disagree | disagree | disagree | disagree | disagree | disagree |
| male | above 41 | Education level below 10 | urban agriculture | less than 1 year | strongly disagree | disagree | Neutral | strongly disagree | Neutral | Neutral | Neutral | Neutral |

| businessrowth | magment1 | magment2 | magment3 | magment4 | magment5 | financial1 | financial2 | financial3 | financial4 | financial5 | market1 | market2 |
| --- | --- | --- | --- | --- | --- | --- | --- | --- | --- | --- | --- | --- |
| strongly disagree | disagree | strongly disagree | strongly agree | strongly agree | strongly disagree | strongly agree | strongly agree | strongly disagree | strongly disagree | strongly agree | strongly disagree | strongly disagree |
| strongly disagree | disagree | strongly disagree | strongly agree | strongly agree | strongly disagree | strongly agree | strongly agree | strongly disagree | strongly disagree | strongly agree | strongly disagree | strongly disagree |
| strongly disagree | disagree | strongly disagree | strongly agree | strongly agree | strongly disagree | strongly agree | strongly agree | strongly disagree | strongly disagree | strongly agree | strongly disagree | strongly disagree |
| strongly disagree | strongly disagree | strongly disagree | strongly disagree | strongly agree | strongly disagree | strongly agree | strongly agree | strongly disagree | strongly disagree | strongly agree | strongly disagree | strongly disagree |
| strongly disagree | strongly disagree | strongly disagree | strongly disagree | strongly agree | strongly disagree | strongly agree | strongly disagree | strongly disagree | strongly disagree | strongly agree | strongly disagree | strongly disagree |
| strongly disagree | strongly disagree | strongly disagree | strongly disagree | strongly agree | strongly disagree | strongly agree | strongly disagree | strongly agree | strongly disagree | strongly agree | strongly disagree | strongly disagree |
| strongly disagree | strongly disagree | strongly disagree | strongly disagree | strongly agree | strongly disagree | strongly agree | strongly disagree | strongly agree | strongly disagree | strongly agree | strongly disagree | strongly disagree |
| strongly disagree | strongly disagree | strongly disagree | strongly disagree | strongly agree | strongly disagree | strongly agree | strongly disagree | strongly agree | strongly disagree | strongly agree | strongly disagree | strongly disagree |
| strongly disagree | strongly disagree | strongly disagree | strongly disagree | strongly agree | strongly disagree | strongly agree | strongly disagree | strongly agree | strongly agree | strongly disagree | strongly disagree | strongly disagree |
| strongly disagree | strongly disagree | strongly disagree | strongly disagree | strongly agree | strongly disagree | strongly agree | strongly disagree | strongly agree | strongly agree | strongly disagree | strongly disagree | strongly disagree |
| strongly disagree | strongly disagree | strongly disagree | strongly disagree | strongly disagree | strongly disagree | strongly agree | strongly disagree | strongly agree | strongly agree | strongly disagree | strongly disagree | strongly disagree |
| strongly disagree | strongly disagree | strongly disagree | strongly disagree | strongly disagree | strongly disagree | strongly agree | strongly disagree | strongly agree | strongly agree | strongly disagree | strongly disagree | strongly disagree |
| strongly disagree | strongly disagree | strongly disagree | strongly disagree | strongly disagree | strongly disagree | strongly agree | strongly disagree | strongly agree | strongly agree | strongly disagree | strongly disagree | strongly disagree |
| strongly disagree | strongly disagree | strongly disagree | strongly disagree | strongly disagree | strongly disagree | strongly agree | strongly disagree | strongly agree | strongly agree | strongly disagree | strongly disagree | strongly disagree |
| strongly disagree | strongly disagree | strongly disagree | strongly disagree | strongly disagree | strongly disagree | strongly agree | strongly disagree | strongly agree | strongly agree | strongly disagree | strongly disagree | strongly disagree |
| strongly disagree | strongly disagree | strongly disagree | strongly disagree | strongly disagree | strongly disagree | strongly agree | strongly disagree | strongly agree | strongly agree | strongly disagree | strongly agree | strongly disagree |
| strongly disagree | strongly disagree | strongly disagree | strongly disagree | strongly disagree | strongly disagree | strongly agree | strongly disagree | strongly agree | strongly agree | strongly disagree | strongly agree | strongly disagree |
| strongly disagree | strongly disagree | strongly disagree | strongly disagree | strongly disagree | strongly disagree | strongly agree | strongly disagree | strongly agree | strongly agree | strongly disagree | strongly agree | strongly disagree |
| strongly disagree | strongly disagree | strongly disagree | strongly disagree | strongly disagree | strongly disagree | strongly agree | strongly disagree | strongly agree | strongly agree | strongly disagree | strongly agree | strongly disagree |
| strongly disagree | strongly disagree | strongly disagree | strongly disagree | strongly disagree | strongly disagree | strongly agree | strongly disagree | strongly agree | strongly agree | strongly disagree | strongly agree | strongly disagree |
| strongly disagree | strongly disagree | strongly disagree | strongly disagree | strongly disagree | strongly disagree | strongly agree | strongly disagree | strongly agree | strongly agree | strongly disagree | strongly agree | strongly disagree |
| strongly disagree | strongly agree | strongly agree | strongly disagree | strongly agree | strongly disagree | strongly agree | strongly disagree | strongly agree | strongly agree | strongly disagree | strongly agree | strongly disagree |
| strongly disagree | strongly agree | strongly agree | strongly disagree | strongly agree | strongly disagree | strongly agree | strongly disagree | strongly agree | strongly agree | strongly disagree | strongly agree | strongly disagree |
| strongly disagree | strongly agree | strongly agree | strongly disagree | strongly agree | strongly disagree | strongly agree | strongly disagree | strongly agree | strongly agree | strongly disagree | strongly agree | strongly disagree |
| strongly disagree | strongly agree | strongly agree | strongly disagree | strongly agree | strongly disagree | strongly agree | strongly disagree | strongly agree | strongly agree | strongly disagree | strongly agree | strongly disagree |
| strongly disagree | strongly agree | strongly agree | strongly disagree | strongly agree | strongly disagree | strongly agree | strongly disagree | strongly agree | strongly agree | strongly disagree | strongly agree | strongly agree |
| strongly disagree | strongly agree | strongly agree | strongly disagree | strongly agree | strongly disagree | strongly agree | strongly disagree | strongly agree | strongly agree | strongly disagree | strongly agree | strongly agree |
| strongly disagree | strongly agree | strongly agree | strongly disagree | strongly agree | strongly disagree | strongly agree | strongly agree | strongly agree | strongly agree | strongly agree | strongly agree | strongly agree |
| strongly disagree | strongly agree | strongly agree | strongly disagree | strongly agree | strongly disagree | strongly agree | strongly agree | strongly agree | strongly agree | strongly agree | strongly agree | strongly agree |
| strongly disagree | strongly agree | strongly agree | strongly disagree | strongly agree | strongly disagree | strongly agree | strongly agree | strongly agree | strongly agree | strongly agree | strongly agree | strongly agree |
| strongly disagree | strongly agree | strongly agree | strongly disagree | strongly agree | strongly disagree | strongly agree | strongly agree | strongly agree | strongly agree | strongly agree | strongly agree | strongly agree |
| strongly disagree | strongly agree | strongly agree | strongly disagree | strongly agree | strongly disagree | strongly agree | strongly agree | strongly agree | strongly agree | strongly agree | strongly agree | strongly agree |
| strongly disagree | strongly agree | strongly agree | strongly disagree | strongly agree | strongly disagree | strongly agree | strongly agree | strongly agree | strongly agree | strongly agree | strongly agree | strongly agree |
| strongly disagree | strongly agree | strongly agree | strongly disagree | strongly agree | strongly disagree | strongly agree | strongly agree | strongly agree | strongly agree | strongly agree | strongly agree | strongly agree |
| strongly agree | strongly agree | strongly agree | strongly agree | strongly agree | strongly agree | strongly agree | strongly agree | strongly agree | strongly agree | strongly agree | strongly agree | strongly agree |
| strongly agree | strongly agree | strongly agree | strongly agree | strongly agree | strongly agree | strongly agree | strongly agree | strongly agree | strongly agree | strongly agree | strongly agree | strongly agree |
| strongly agree | strongly agree | strongly agree | strongly agree | strongly agree | strongly agree | strongly agree | strongly agree | strongly agree | strongly agree | strongly agree | strongly agree | strongly agree |
| strongly agree | strongly agree | strongly agree | strongly agree | strongly agree | strongly agree | strongly agree | strongly agree | strongly agree | strongly agree | strongly agree | strongly agree | strongly agree |
| strongly agree | strongly agree | strongly agree | strongly agree | strongly agree | strongly agree | strongly agree | strongly agree | strongly agree | strongly agree | strongly agree | strongly agree | strongly agree |
| strongly agree | strongly agree | strongly agree | strongly agree | strongly agree | strongly disagree | strongly agree | strongly agree | strongly agree | strongly agree | strongly agree | strongly agree | strongly agree |
| strongly agree | strongly agree | strongly agree | strongly agree | strongly agree | strongly disagree | strongly agree | strongly agree | strongly agree | strongly agree | strongly agree | strongly agree | strongly agree |
| strongly agree | strongly agree | strongly agree | strongly agree | strongly agree | strongly disagree | strongly agree | strongly agree | strongly agree | strongly agree | strongly agree | strongly agree | strongly agree |
| strongly agree | strongly agree | strongly agree | strongly agree | strongly agree | strongly agree | strongly agree | strongly agree | strongly agree | strongly agree | strongly agree | strongly agree | strongly agree |
| strongly agree | strongly agree | strongly agree | strongly agree | strongly agree | strongly agree | strongly agree | strongly agree | strongly agree | strongly agree | strongly agree | strongly agree | strongly agree |
| strongly agree | strongly agree | strongly agree | strongly agree | strongly agree | strongly agree | strongly agree | strongly agree | strongly agree | strongly agree | strongly agree | strongly agree | strongly agree |
| strongly agree | strongly agree | strongly agree | strongly agree | strongly agree | strongly agree | strongly agree | strongly agree | strongly agree | strongly agree | strongly agree | strongly agree | strongly agree |
| strongly agree | strongly agree | strongly agree | strongly agree | strongly agree | strongly agree | strongly agree | strongly agree | strongly agree | strongly agree | strongly agree | strongly agree | strongly agree |
| strongly agree | strongly agree | strongly agree | strongly agree | strongly agree | strongly agree | strongly agree | strongly agree | strongly agree | strongly agree | strongly agree | strongly agree | strongly agree |
| strongly agree | strongly agree | strongly agree | strongly agree | strongly agree | strongly agree | strongly agree | strongly agree | strongly agree | strongly agree | strongly agree | strongly agree | strongly agree |
| strongly agree | strongly agree | strongly agree | strongly agree | strongly agree | strongly agree | strongly agree | strongly agree | strongly agree | strongly agree | strongly agree | strongly agree | strongly agree |
| strongly agree | strongly agree | strongly agree | strongly agree | strongly agree | strongly agree | strongly agree | strongly agree | strongly agree | strongly agree | strongly agree | strongly agree | strongly agree |
| strongly agree | strongly agree | strongly agree | strongly agree | strongly agree | strongly agree | strongly agree | strongly agree | strongly agree | strongly agree | strongly agree | strongly agree | strongly agree |
| strongly agree | strongly agree | strongly agree | strongly agree | strongly agree | strongly agree | strongly agree | strongly agree | strongly agree | strongly agree | strongly agree | strongly agree | strongly agree |
| strongly agree | strongly agree | strongly agree | strongly agree | strongly agree | strongly agree | strongly agree | strongly agree | strongly agree | strongly agree | strongly agree | strongly agree | strongly agree |
| strongly agree | strongly agree | strongly agree | strongly agree | strongly agree | strongly agree | strongly agree | strongly agree | strongly agree | strongly agree | strongly agree | strongly agree | strongly agree |
| strongly agree | strongly agree | strongly agree | strongly agree | strongly agree | strongly agree | strongly agree | strongly agree | strongly agree | strongly agree | strongly agree | strongly agree | strongly agree |
| strongly agree | strongly agree | strongly agree | strongly agree | strongly agree | strongly agree | strongly agree | strongly agree | strongly agree | strongly agree | strongly agree | strongly agree | strongly agree |
| strongly agree | strongly agree | strongly agree | strongly agree | strongly agree | strongly agree | strongly agree | strongly agree | strongly agree | strongly agree | strongly agree | strongly agree | strongly agree |
| strongly agree | strongly agree | strongly agree | strongly agree | strongly agree | strongly agree | strongly agree | strongly agree | strongly agree | strongly agree | strongly agree | strongly agree | strongly agree |
| strongly agree | strongly agree | strongly agree | strongly agree | strongly agree | strongly agree | strongly agree | strongly agree | strongly agree | strongly agree | strongly agree | strongly agree | strongly agree |
| strongly agree | strongly agree | strongly agree | strongly agree | strongly agree | strongly agree | strongly agree | strongly agree | strongly agree | strongly agree | strongly agree | strongly agree | strongly agree |
| strongly agree | strongly agree | strongly agree | strongly agree | strongly agree | strongly agree | strongly agree | strongly agree | strongly agree | strongly agree | strongly agree | strongly agree | strongly agree |
| strongly agree | strongly agree | strongly agree | strongly agree | strongly agree | strongly agree | strongly agree | strongly agree | strongly agree | strongly agree | strongly agree | strongly agree | strongly agree |
| strongly agree | strongly agree | strongly agree | strongly agree | strongly agree | strongly agree | strongly agree | strongly agree | strongly agree | strongly agree | strongly agree | strongly agree | strongly agree |
| strongly agree | strongly agree | strongly agree | strongly agree | strongly agree | strongly agree | strongly agree | strongly agree | strongly agree | strongly agree | strongly agree | strongly agree | strongly agree |
| strongly agree | strongly agree | strongly agree | strongly agree | strongly agree | strongly agree | strongly agree | strongly agree | strongly agree | strongly agree | strongly agree | strongly agree | strongly agree |
| strongly agree | strongly agree | strongly agree | strongly agree | strongly agree | strongly agree | strongly agree | strongly agree | strongly agree | strongly agree | strongly agree | strongly agree | strongly agree |
| strongly agree | strongly agree | strongly agree | strongly agree | strongly agree | strongly agree | strongly agree | strongly agree | strongly agree | strongly agree | strongly agree | strongly agree | strongly agree |
| strongly agree | strongly agree | strongly agree | strongly agree | strongly agree | strongly agree | strongly agree | strongly agree | strongly agree | strongly agree | strongly agree | strongly agree | strongly agree |
| strongly agree | strongly agree | strongly agree | strongly agree | strongly agree | strongly agree | strongly agree | strongly agree | strongly agree | strongly agree | strongly agree | strongly agree | strongly agree |
| strongly agree | strongly agree | strongly agree | strongly agree | strongly agree | strongly agree | strongly agree | strongly agree | strongly agree | strongly agree | strongly agree | strongly agree | strongly agree |
| strongly agree | strongly agree | strongly agree | strongly agree | strongly agree | strongly agree | strongly agree | strongly agree | strongly agree | strongly agree | strongly agree | strongly agree | strongly agree |
| strongly agree | strongly agree | strongly agree | strongly agree | strongly agree | strongly agree | strongly agree | strongly agree | strongly agree | strongly agree | strongly agree | strongly agree | strongly agree |
| strongly agree | strongly agree | strongly agree | strongly agree | strongly agree | strongly agree | strongly agree | strongly agree | strongly agree | strongly agree | strongly agree | strongly agree | strongly agree |
| strongly agree | strongly agree | strongly agree | strongly agree | strongly agree | strongly agree | strongly agree | strongly agree | strongly agree | strongly agree | strongly agree | strongly agree | strongly agree |
| strongly agree | strongly agree | strongly agree | strongly agree | strongly agree | strongly agree | strongly agree | strongly agree | strongly agree | strongly agree | strongly agree | strongly agree | strongly agree |
| strongly agree | strongly agree | strongly agree | strongly agree | strongly agree | strongly agree | strongly agree | strongly agree | strongly agree | strongly agree | strongly agree | strongly agree | strongly agree |
| strongly agree | strongly agree | strongly agree | strongly agree | strongly agree | strongly agree | strongly agree | strongly agree | strongly agree | strongly agree | strongly agree | strongly agree | strongly agree |
| strongly agree | strongly agree | strongly agree | strongly agree | strongly agree | strongly agree | strongly agree | strongly agree | strongly agree | strongly agree | strongly agree | strongly agree | strongly agree |
| strongly agree | strongly agree | strongly agree | strongly agree | strongly agree | strongly agree | strongly agree | strongly agree | strongly agree | strongly agree | strongly agree | strongly agree | strongly agree |
| strongly agree | strongly agree | strongly agree | strongly agree | strongly agree | strongly agree | strongly agree | strongly agree | strongly agree | strongly agree | strongly agree | strongly agree | strongly agree |
| strongly agree | strongly agree | strongly agree | strongly agree | strongly agree | strongly agree | strongly agree | strongly agree | strongly agree | strongly agree | strongly agree | strongly agree | strongly agree |
| strongly agree | strongly agree | strongly agree | strongly agree | strongly agree | strongly agree | strongly agree | strongly agree | strongly agree | strongly agree | strongly agree | strongly agree | strongly agree |
| strongly agree | strongly agree | strongly agree | strongly agree | strongly agree | strongly agree | strongly agree | strongly agree | strongly agree | strongly agree | strongly agree | strongly agree | strongly agree |
| strongly agree | strongly agree | strongly agree | strongly agree | strongly agree | strongly agree | strongly agree | strongly agree | strongly agree | strongly agree | strongly agree | strongly agree | strongly agree |
| strongly agree | strongly agree | strongly agree | strongly agree | strongly agree | strongly agree | strongly agree | strongly agree | strongly agree | strongly agree | strongly agree | strongly agree | strongly agree |
| strongly agree | strongly agree | strongly agree | strongly agree | strongly agree | strongly agree | strongly agree | strongly agree | strongly agree | strongly agree | strongly agree | strongly agree | strongly agree |
| strongly agree | strongly agree | strongly agree | strongly agree | strongly agree | strongly agree | strongly agree | strongly agree | strongly agree | strongly agree | strongly agree | strongly agree | strongly agree |
| strongly agree | strongly agree | strongly agree | strongly agree | strongly agree | strongly agree | strongly agree | strongly agree | strongly agree | strongly agree | strongly agree | strongly agree | strongly agree |
| strongly agree | strongly agree | strongly agree | strongly agree | strongly agree | strongly agree | strongly agree | strongly agree | strongly agree | strongly agree | strongly agree | strongly agree | strongly agree |
| strongly agree | strongly agree | strongly agree | strongly agree | strongly agree | strongly agree | strongly agree | strongly agree | strongly agree | strongly agree | strongly agree | strongly agree | strongly agree |
| strongly agree | strongly agree | strongly agree | strongly agree | strongly agree | strongly agree | strongly agree | strongly agree | strongly agree | strongly agree | strongly agree | strongly agree | strongly agree |
| strongly agree | strongly agree | strongly agree | strongly agree | strongly agree | strongly agree | strongly agree | strongly agree | strongly agree | strongly agree | strongly agree | strongly agree | strongly agree |
| strongly agree | strongly agree | strongly agree | strongly agree | strongly agree | strongly agree | strongly agree | strongly agree | strongly agree | strongly agree | strongly agree | strongly agree | strongly agree |
| strongly agree | strongly agree | strongly agree | strongly agree | strongly agree | strongly agree | strongly agree | strongly agree | strongly agree | strongly agree | strongly agree | strongly agree | strongly agree |
| strongly agree | strongly agree | strongly agree | strongly agree | strongly agree | strongly agree | strongly agree | strongly agree | strongly agree | strongly agree | strongly agree | strongly agree | strongly agree |
| strongly agree | strongly agree | strongly agree | strongly agree | strongly agree | strongly agree | strongly agree | strongly agree | strongly agree | strongly agree | strongly agree | strongly agree | strongly agree |
| strongly agree | strongly agree | strongly agree | strongly agree | strongly agree | strongly agree | strongly agree | strongly agree | strongly agree | strongly agree | strongly agree | strongly agree | strongly agree |
| strongly agree | strongly agree | strongly agree | strongly agree | strongly agree | strongly agree | strongly agree | strongly agree | strongly agree | strongly agree | strongly agree | strongly agree | strongly agree |
| strongly agree | strongly agree | strongly agree | strongly agree | strongly agree | strongly agree | strongly agree | strongly agree | strongly agree | strongly agree | strongly agree | strongly agree | strongly agree |
| strongly agree | strongly agree | strongly agree | strongly agree | strongly agree | strongly agree | strongly agree | strongly agree | strongly agree | strongly agree | strongly agree | strongly agree | strongly agree |
| strongly agree | strongly agree | strongly agree | strongly agree | strongly agree | strongly agree | strongly agree | strongly agree | strongly agree | strongly agree | strongly agree | strongly agree | strongly agree |
| strongly agree | strongly agree | strongly agree | strongly agree | strongly agree | strongly agree | strongly agree | strongly agree | strongly agree | strongly agree | strongly agree | strongly agree | strongly agree |
| strongly agree | strongly agree | strongly agree | strongly agree | strongly agree | strongly agree | strongly agree | strongly agree | strongly agree | strongly agree | strongly agree | strongly agree | strongly agree |
| strongly agree | strongly agree | strongly agree | strongly agree | strongly agree | strongly agree | strongly agree | strongly agree | strongly agree | strongly agree | strongly agree | strongly agree | strongly agree |
| strongly agree | strongly agree | strongly agree | strongly agree | strongly agree | strongly agree | strongly agree | strongly agree | strongly agree | strongly agree | strongly agree | strongly agree | strongly agree |
| strongly agree | strongly agree | strongly agree | strongly agree | strongly agree | strongly agree | strongly agree | strongly agree | strongly agree | strongly agree | strongly agree | strongly agree | strongly agree |
| strongly agree | strongly agree | strongly agree | strongly agree | strongly agree | strongly agree | strongly agree | strongly agree | strongly agree | strongly agree | strongly agree | strongly agree | strongly agree |
| strongly agree | strongly agree | strongly agree | strongly agree | strongly agree | strongly agree | strongly agree | strongly agree | strongly agree | strongly agree | strongly agree | strongly agree | strongly agree |
| strongly agree | strongly agree | strongly agree | strongly agree | strongly agree | strongly agree | strongly agree | strongly agree | strongly agree | strongly agree | strongly agree | strongly agree | strongly agree |
| strongly agree | strongly agree | strongly agree | strongly agree | strongly agree | strongly agree | strongly agree | strongly agree | strongly agree | strongly agree | strongly agree | strongly agree | strongly agree |
| strongly agree | strongly agree | strongly agree | strongly agree | strongly agree | strongly agree | strongly agree | strongly agree | strongly agree | strongly agree | strongly agree | strongly agree | strongly agree |
| strongly agree | strongly agree | strongly agree | strongly agree | strongly agree | strongly agree | strongly agree | strongly agree | strongly agree | strongly agree | strongly agree | strongly agree | strongly agree |
| strongly agree | strongly agree | strongly agree | strongly agree | strongly agree | strongly agree | strongly agree | strongly agree | strongly agree | strongly agree | strongly agree | strongly agree | strongly agree |
| strongly agree | strongly agree | strongly agree | strongly agree | strongly agree | strongly agree | strongly agree | strongly agree | strongly agree | strongly agree | strongly agree | strongly agree | strongly agree |
| strongly agree | strongly agree | strongly agree | strongly agree | strongly agree | strongly agree | strongly agree | strongly agree | strongly agree | strongly agree | strongly agree | strongly agree | strongly agree |
| strongly agree | strongly agree | strongly agree | strongly agree | strongly agree | strongly agree | strongly agree | strongly agree | strongly agree | strongly agree | strongly agree | strongly agree | strongly agree |
| strongly agree | strongly agree | strongly agree | strongly agree | strongly agree | strongly agree | strongly agree | strongly agree | strongly agree | strongly agree | strongly agree | strongly agree | strongly agree |
| strongly agree | strongly agree | strongly agree | strongly agree | strongly agree | strongly agree | strongly agree | strongly agree | strongly agree | strongly agree | strongly agree | strongly agree | strongly agree |
| strongly agree | strongly agree | strongly agree | strongly agree | strongly agree | strongly agree | strongly agree | strongly agree | strongly agree | strongly agree | strongly agree | strongly agree | strongly agree |
| strongly agree | strongly agree | strongly agree | strongly agree | strongly agree | strongly agree | strongly agree | strongly agree | strongly agree | strongly agree | strongly agree | strongly agree | strongly agree |
| strongly agree | strongly agree | strongly agree | strongly agree | strongly agree | strongly agree | strongly agree | strongly agree | strongly agree | strongly agree | strongly agree | strongly agree | strongly agree |
| strongly agree | strongly agree | strongly agree | strongly agree | strongly agree | strongly agree | strongly agree | strongly agree | strongly agree | strongly agree | strongly agree | strongly agree | strongly agree |
| strongly agree | strongly agree | strongly agree | strongly agree | strongly agree | strongly agree | strongly agree | strongly agree | strongly agree | strongly agree | strongly agree | strongly agree | strongly agree |
| strongly agree | strongly agree | strongly agree | strongly agree | strongly agree | strongly agree | strongly agree | strongly agree | strongly agree | strongly agree | strongly agree | strongly agree | strongly agree |
| strongly agree | strongly agree | strongly agree | strongly agree | strongly agree | strongly agree | strongly agree | strongly agree | strongly agree | strongly agree | strongly agree | strongly agree | strongly agree |
| strongly agree | strongly agree | strongly agree | strongly agree | strongly agree | strongly agree | strongly agree | strongly agree | strongly agree | strongly agree | strongly agree | strongly agree | strongly agree |
| strongly agree | strongly agree | strongly agree | strongly agree | strongly agree | strongly agree | strongly agree | strongly agree | strongly agree | strongly agree | strongly agree | strongly agree | strongly agree |
| strongly agree | strongly agree | strongly agree | strongly agree | strongly agree | strongly agree | strongly agree | strongly agree | strongly agree | strongly agree | strongly agree | strongly agree | strongly agree |
| strongly agree | strongly agree | strongly agree | strongly agree | strongly agree | strongly agree | strongly agree | strongly agree | strongly agree | strongly agree | strongly agree | strongly agree | strongly agree |
| strongly agree | strongly agree | strongly agree | strongly agree | strongly agree | strongly agree | strongly agree | strongly agree | strongly agree | strongly agree | strongly agree | strongly agree | strongly agree |
| strongly agree | strongly agree | strongly agree | strongly agree | strongly agree | strongly agree | strongly agree | strongly agree | strongly agree | strongly agree | strongly agree | strongly agree | strongly agree |
| strongly agree | strongly agree | strongly agree | strongly agree | strongly agree | strongly agree | strongly agree | strongly agree | strongly agree | strongly agree | strongly agree | strongly agree | strongly agree |
| strongly agree | strongly agree | strongly agree | strongly agree | strongly agree | strongly agree | strongly agree | strongly agree | strongly agree | strongly agree | strongly agree | strongly agree | strongly agree |
| strongly agree | strongly agree | strongly agree | strongly agree | strongly agree | strongly agree | strongly agree | strongly agree | strongly agree | strongly agree | strongly agree | strongly agree | strongly agree |
| strongly agree | strongly agree | strongly agree | strongly agree | strongly agree | strongly agree | strongly agree | strongly agree | strongly agree | strongly agree | strongly agree | strongly agree | strongly agree |
| strongly agree | strongly agree | strongly agree | strongly agree | strongly agree | strongly agree | strongly agree | strongly agree | strongly agree | strongly agree | strongly agree | strongly agree | strongly agree |
| strongly agree | strongly agree | strongly agree | strongly agree | strongly agree | strongly agree | strongly agree | strongly agree | strongly agree | strongly agree | strongly agree | strongly agree | strongly agree |
| strongly agree | strongly agree | strongly agree | strongly agree | strongly agree | strongly agree | strongly agree | strongly agree | strongly agree | strongly agree | strongly agree | strongly agree | strongly agree |
| strongly agree | strongly agree | strongly agree | strongly agree | strongly agree | strongly agree | strongly agree | strongly agree | strongly agree | strongly agree | strongly agree | strongly agree | strongly agree |
| strongly agree | strongly agree | strongly agree | strongly agree | strongly agree | strongly agree | strongly agree | strongly agree | strongly agree | strongly agree | strongly agree | strongly agree | strongly agree |
| strongly agree | strongly agree | strongly agree | strongly agree | strongly agree | strongly agree | strongly agree | strongly agree | strongly agree | strongly agree | strongly agree | strongly agree | strongly agree |
| strongly agree | strongly agree | strongly agree | strongly agree | strongly agree | strongly agree | strongly agree | strongly agree | strongly agree | strongly agree | strongly agree | strongly agree | strongly agree |
| strongly agree | strongly agree | strongly agree | strongly agree | strongly agree | strongly agree | strongly agree | strongly agree | strongly agree | strongly agree | strongly agree | strongly agree | strongly agree |
| strongly agree | strongly agree | strongly agree | strongly agree | strongly agree | strongly agree | strongly agree | strongly agree | strongly agree | strongly agree | strongly agree | strongly agree | strongly agree |
| strongly agree | strongly agree | strongly agree | strongly agree | strongly agree | strongly agree | strongly agree | strongly agree | strongly agree | strongly agree | strongly agree | strongly agree | strongly agree |
| strongly agree | strongly agree | strongly agree | strongly agree | strongly agree | strongly agree | strongly agree | strongly agree | strongly agree | strongly agree | strongly agree | strongly agree | strongly agree |
| strongly agree | strongly agree | strongly agree | strongly agree | strongly agree | strongly agree | strongly agree | strongly agree | strongly agree | strongly agree | strongly agree | strongly agree | strongly agree |
| strongly agree | strongly agree | strongly agree | strongly agree | strongly agree | strongly agree | strongly agree | strongly agree | strongly agree | strongly agree | strongly agree | strongly agree | strongly agree |
| strongly agree | strongly agree | strongly agree | strongly agree | strongly agree | strongly agree | strongly agree | strongly agree | strongly agree | strongly agree | strongly agree | strongly agree | strongly agree |
| strongly agree | strongly agree | strongly agree | strongly agree | strongly agree | strongly agree | strongly agree | strongly agree | strongly agree | strongly agree | strongly agree | strongly agree | strongly agree |
| strongly agree | strongly agree | strongly agree | strongly agree | strongly agree | strongly agree | strongly agree | strongly agree | strongly agree | strongly agree | strongly agree | strongly agree | strongly agree |
| strongly agree | strongly agree | strongly agree | strongly agree | strongly agree | strongly agree | strongly agree | strongly agree | strongly agree | strongly agree | strongly agree | strongly agree | strongly agree |
| strongly agree | strongly agree | strongly agree | strongly agree | strongly agree | strongly agree | strongly agree | strongly agree | strongly agree | strongly agree | strongly agree | strongly agree | strongly agree |
| strongly agree | strongly agree | strongly agree | strongly agree | strongly agree | strongly agree | strongly agree | strongly agree | strongly agree | strongly agree | strongly agree | strongly agree | strongly agree |
| strongly agree | strongly agree | strongly agree | strongly agree | strongly agree | strongly agree | strongly agree | strongly agree | strongly agree | strongly agree | strongly agree | strongly agree | strongly agree |
| strongly agree | strongly agree | strongly agree | strongly agree | strongly agree | strongly agree | strongly agree | strongly agree | strongly agree | strongly agree | strongly agree | strongly agree | strongly agree |
| strongly agree | strongly agree | strongly agree | strongly agree | strongly agree | strongly agree | strongly agree | strongly agree | strongly agree | strongly agree | strongly agree | strongly agree | strongly agree |
| strongly agree | strongly agree | strongly agree | strongly agree | strongly agree | strongly agree | strongly agree | strongly agree | strongly agree | strongly agree | strongly agree | strongly agree | strongly agree |
| strongly agree | strongly agree | strongly agree | strongly agree | strongly agree | strongly agree | strongly agree | strongly agree | strongly agree | strongly agree | strongly agree | strongly agree | strongly agree |
| strongly agree | strongly agree | strongly agree | strongly agree | strongly agree | strongly agree | strongly agree | strongly agree | strongly agree | strongly agree | strongly agree | strongly agree | strongly agree |
| strongly agree | strongly agree | strongly agree | strongly agree | strongly agree | strongly agree | strongly agree | strongly agree | strongly agree | strongly agree | strongly agree | strongly agree | strongly agree |
| strongly agree | strongly agree | strongly agree | strongly agree | strongly agree | strongly agree | strongly agree | strongly agree | strongly agree | strongly agree | strongly agree | strongly agree | strongly agree |
| strongly agree | strongly agree | strongly agree | strongly agree | strongly agree | strongly agree | strongly agree | strongly agree | strongly agree | strongly agree | strongly agree | strongly agree | strongly agree |
| strongly agree | strongly agree | strongly agree | strongly agree | strongly agree | strongly agree | strongly agree | strongly agree | strongly agree | strongly agree | strongly agree | strongly agree | strongly agree |
| strongly agree | strongly agree | strongly agree | strongly agree | strongly agree | strongly agree | strongly agree | strongly agree | strongly agree | strongly agree | strongly agree | strongly agree | strongly agree |
| strongly agree | strongly agree | strongly agree | strongly agree | strongly agree | strongly agree | strongly agree | strongly agree | strongly agree | strongly agree | strongly agree | strongly agree | strongly agree |
| strongly agree | strongly agree | strongly agree | strongly agree | strongly agree | strongly agree | strongly agree | strongly agree | strongly agree | strongly agree | strongly agree | strongly agree | strongly agree |
| strongly agree | strongly agree | strongly agree | strongly agree | strongly agree | strongly agree | strongly agree | strongly agree | strongly agree | strongly agree | strongly agree | strongly agree | strongly agree |
| strongly agree | strongly agree | strongly agree | strongly agree | strongly agree | strongly agree | strongly agree | strongly agree | strongly agree | strongly agree | strongly agree | strongly agree | strongly agree |
| strongly agree | strongly agree | strongly agree | strongly agree | strongly agree | strongly agree | strongly agree | strongly agree | strongly agree | strongly agree | strongly agree | strongly agree | strongly agree |
| strongly agree | strongly agree | strongly agree | strongly agree | strongly agree | strongly agree | strongly agree | strongly agree | strongly agree | strongly agree | strongly agree | strongly agree | strongly agree |
| strongly agree | strongly agree | strongly agree | strongly agree | strongly agree | strongly agree | strongly agree | strongly agree | strongly agree | strongly agree | strongly agree | strongly agree | strongly agree |
| strongly agree | strongly agree | strongly agree | strongly agree | strongly agree | strongly agree | strongly agree | strongly agree | strongly agree | strongly agree | strongly agree | strongly agree | strongly agree |
| strongly agree | strongly agree | strongly agree | strongly agree | strongly agree | strongly agree | strongly agree | strongly agree | strongly agree | strongly agree | strongly agree | strongly agree | strongly agree |
| strongly agree | strongly agree | strongly agree | strongly agree | strongly agree | strongly agree | strongly agree | strongly agree | strongly agree | strongly agree | strongly agree | strongly agree | strongly agree |
| strongly agree | strongly agree | strongly agree | strongly agree | strongly agree | strongly agree | strongly agree | strongly agree | strongly agree | strongly agree | strongly agree | strongly agree | strongly agree |
| strongly agree | strongly agree | strongly agree | strongly agree | strongly agree | strongly agree | strongly agree | strongly agree | strongly agree | strongly agree | strongly agree | strongly agree | strongly agree |
| strongly agree | strongly agree | strongly agree | strongly agree | strongly agree | strongly agree | strongly agree | strongly agree | strongly agree | strongly agree | strongly agree | strongly agree | strongly agree |
| strongly agree | strongly agree | strongly agree | strongly agree | strongly agree | strongly agree | strongly agree | strongly agree | strongly agree | strongly agree | strongly agree | strongly agree | strongly agree |
| strongly agree | strongly agree | strongly agree | strongly agree | strongly agree | strongly agree | strongly agree | strongly agree | strongly agree | strongly agree | strongly agree | strongly agree | strongly agree |
| strongly agree | strongly agree | strongly agree | strongly agree | strongly agree | strongly agree | strongly agree | strongly agree | strongly agree | strongly agree | strongly agree | strongly agree | strongly agree |
| strongly agree | strongly agree | strongly agree | strongly agree | strongly agree | strongly agree | strongly agree | strongly agree | strongly agree | strongly agree | strongly agree | strongly agree | strongly agree |
| strongly agree | strongly agree | strongly agree | strongly agree | strongly agree | strongly agree | strongly agree | strongly agree | strongly agree | strongly agree | strongly agree | strongly agree | strongly agree |
| strongly agree | strongly agree | strongly agree | strongly agree | strongly agree | strongly agree | strongly agree | strongly agree | strongly agree | strongly agree | strongly agree | strongly agree | strongly agree |
| strongly agree | strongly agree | strongly agree | strongly agree | strongly agree | strongly agree | strongly agree | strongly agree | strongly agree | strongly agree | strongly agree | strongly agree | strongly agree |
| strongly agree | strongly agree | strongly agree | strongly agree | strongly agree | strongly agree | strongly agree | strongly agree | strongly agree | strongly agree | strongly agree | strongly agree | strongly agree |
| strongly agree | strongly agree | strongly agree | strongly agree | strongly agree | strongly agree | strongly agree | strongly agree | strongly agree | strongly agree | strongly agree | strongly agree | strongly agree |
| strongly agree | strongly agree | strongly agree | strongly agree | strongly agree | strongly agree | strongly agree | strongly agree | strongly agree | strongly agree | strongly agree | strongly agree | strongly agree |
| strongly agree | strongly agree | strongly agree | strongly agree | strongly agree | strongly agree | strongly agree | strongly agree | strongly agree | strongly agree | strongly agree | strongly agree | strongly agree |
| strongly agree | strongly agree | strongly agree | strongly agree | strongly agree | strongly agree | strongly agree | strongly agree | strongly agree | strongly agree | strongly agree | strongly agree | strongly agree |
| strongly agree | strongly agree | strongly agree | strongly agree | strongly agree | strongly agree | strongly agree | strongly agree | strongly agree | strongly agree | strongly agree | strongly agree | strongly agree |
| strongly agree | strongly agree | strongly agree | strongly agree | strongly agree | strongly agree | strongly agree | strongly agree | strongly agree | strongly agree | strongly agree | strongly agree | strongly agree |
| strongly agree | strongly agree | strongly agree | strongly agree | strongly agree | strongly agree | strongly agree | strongly agree | strongly agree | strongly agree | strongly agree | strongly agree | strongly agree |
| strongly agree | strongly agree | strongly agree | strongly agree | strongly agree | strongly agree | strongly agree | strongly agree | strongly agree | strongly agree | strongly agree | strongly agree | strongly agree |
| strongly agree | strongly agree | strongly agree | strongly agree | strongly agree | strongly agree | strongly agree | strongly agree | strongly agree | strongly agree | strongly agree | strongly agree | strongly agree |
| strongly agree | strongly agree | strongly agree | strongly agree | strongly agree | strongly agree | strongly agree | strongly agree | strongly agree | strongly agree | strongly agree | strongly agree | strongly agree |
| strongly agree | strongly agree | strongly agree | strongly agree | strongly agree | strongly agree | strongly agree | strongly agree | strongly agree | strongly agree | strongly agree | strongly agree | strongly agree |
| strongly agree | strongly agree | strongly agree | strongly agree | strongly agree | strongly agree | strongly agree | strongly agree | strongly agree | strongly agree | strongly agree | strongly agree | strongly agree |
| strongly agree | strongly agree | strongly agree | strongly agree | strongly agree | strongly agree | strongly agree | strongly agree | strongly agree | strongly agree | strongly agree | strongly agree | strongly agree |
| strongly agree | strongly agree | strongly agree | strongly agree | strongly agree | strongly agree | strongly agree | strongly agree | strongly agree | strongly agree | strongly agree | strongly agree | strongly agree |
| strongly agree | strongly agree | strongly agree | strongly agree | strongly agree | strongly agree | strongly agree | strongly agree | strongly agree | strongly agree | strongly agree | strongly agree | strongly agree |
| strongly agree | strongly agree | strongly agree | strongly agree | strongly agree | strongly agree | strongly agree | strongly agree | strongly agree | strongly agree | strongly agree | strongly agree | strongly agree |
| strongly agree | strongly agree | strongly agree | strongly agree | strongly agree | strongly agree | strongly agree | strongly agree | strongly agree | strongly agree | strongly agree | strongly agree | strongly agree |
| strongly agree | strongly agree | strongly agree | strongly agree | strongly agree | strongly agree | strongly agree | strongly agree | strongly agree | strongly agree | strongly agree | strongly agree | strongly agree |
| strongly agree | strongly agree | strongly agree | strongly agree | strongly agree | strongly agree | strongly agree | strongly agree | strongly agree | strongly agree | strongly agree | strongly agree | strongly agree |
| strongly agree | strongly agree | strongly agree | strongly agree | strongly agree | strongly agree | strongly agree | strongly agree | strongly agree | strongly agree | strongly agree | strongly agree | strongly agree |
| strongly agree | strongly agree | strongly agree | strongly agree | strongly agree | strongly agree | strongly agree | strongly agree | strongly agree | strongly agree | strongly agree | strongly agree | strongly agree |
| strongly agree | strongly agree | strongly agree | strongly agree | strongly agree | strongly agree | strongly agree | strongly agree | strongly agree | strongly agree | strongly agree | strongly agree | strongly agree |
| strongly agree | strongly agree | strongly agree | strongly agree | strongly agree | strongly agree | strongly agree | strongly agree | strongly agree | strongly agree | strongly agree | strongly agree | strongly agree |
| strongly agree | strongly agree | strongly agree | strongly agree | strongly agree | strongly agree | strongly agree | strongly agree | strongly agree | strongly agree | strongly agree | strongly agree | strongly agree |
| strongly agree | strongly agree | strongly agree | strongly agree | strongly agree | strongly agree | strongly agree | strongly agree | strongly agree | strongly agree | strongly agree | strongly agree | strongly agree |
| strongly agree | strongly agree | strongly agree | strongly agree | strongly agree | strongly agree | strongly agree | strongly agree | strongly agree | strongly agree | strongly agree | strongly agree | strongly agree |
| strongly agree | strongly agree | strongly agree | strongly agree | strongly agree | strongly agree | strongly agree | strongly agree | strongly agree | strongly agree | strongly agree | strongly agree | strongly agree |
| strongly agree | strongly agree | strongly agree | strongly agree | strongly agree | strongly agree | strongly agree | strongly agree | strongly agree | strongly agree | strongly agree | strongly agree | strongly agree |
| strongly agree | strongly agree | strongly agree | strongly agree | strongly agree | strongly agree | strongly agree | strongly agree | strongly agree | strongly agree | strongly agree | strongly agree | strongly agree |
| strongly agree | strongly agree | strongly agree | strongly agree | strongly agree | strongly agree | strongly agree | strongly agree | strongly agree | strongly agree | strongly agree | strongly agree | strongly agree |
| strongly agree | strongly agree | strongly agree | strongly agree | strongly agree | strongly agree | strongly agree | strongly agree | strongly agree | strongly agree | strongly agree | strongly agree | strongly agree |
| strongly agree | strongly agree | strongly agree | strongly agree | strongly agree | strongly agree | strongly agree | strongly agree | strongly agree | strongly agree | strongly agree | strongly agree | strongly agree |
| strongly agree | strongly agree | strongly agree | strongly agree | strongly agree | strongly agree | strongly agree | strongly agree | strongly agree | strongly agree | strongly agree | strongly agree | strongly agree |
| strongly agree | strongly agree | strongly agree | strongly agree | strongly agree | strongly agree | strongly agree | strongly agree | strongly agree | strongly agree | strongly agree | strongly agree | strongly agree |
| strongly agree | strongly agree | strongly agree | strongly agree | strongly agree | strongly agree | strongly agree | strongly agree | strongly agree | strongly agree | strongly agree | strongly agree | strongly agree |
| strongly agree | strongly agree | strongly agree | strongly agree | strongly agree | strongly agree | strongly agree | strongly agree | strongly agree | strongly agree | strongly agree | strongly agree | strongly agree |
| strongly agree | strongly agree | strongly agree | strongly agree | strongly agree | strongly agree | strongly agree | strongly agree | strongly agree | strongly agree | strongly agree | strongly agree | strongly agree |
| strongly agree | strongly agree | strongly agree | strongly agree | strongly agree | strongly agree | strongly agree | strongly agree | strongly agree | strongly agree | strongly agree | strongly agree | strongly agree |
| strongly agree | strongly agree | strongly agree | strongly agree | strongly agree | strongly agree | strongly agree | strongly agree | strongly agree | strongly agree | strongly agree | strongly agree | strongly agree |
| strongly agree | strongly agree | strongly agree | strongly agree | strongly agree | strongly agree | strongly agree | strongly agree | strongly agree | strongly agree | strongly agree | strongly agree | strongly agree |
| strongly agree | strongly agree | strongly agree | strongly agree | strongly agree | strongly agree | strongly agree | strongly agree | strongly agree | strongly agree | strongly agree | strongly agree | strongly agree |
| strongly agree | strongly agree | strongly agree | strongly agree | strongly agree | strongly agree | strongly agree | strongly agree | strongly agree | strongly agree | strongly agree | strongly agree | strongly agree |
| strongly agree | strongly agree | strongly agree | strongly agree | strongly agree | strongly agree | strongly agree | strongly agree | strongly agree | strongly agree | strongly agree | strongly agree | strongly agree |
| strongly agree | strongly agree | strongly agree | strongly agree | strongly agree | strongly agree | strongly agree | strongly agree | strongly agree | strongly agree | strongly agree | strongly agree | strongly agree |
| strongly agree | strongly agree | strongly agree | strongly agree | strongly agree | strongly agree | strongly agree | strongly agree | strongly agree | strongly agree | strongly agree | strongly agree | strongly agree |
| strongly agree | strongly agree | strongly agree | strongly agree | strongly agree | strongly agree | strongly agree | strongly agree | strongly agree | strongly agree | strongly agree | strongly agree | strongly agree |
| strongly agree | strongly agree | strongly agree | strongly agree | strongly agree | strongly agree | strongly agree | strongly agree | strongly agree | strongly agree | strongly agree | strongly agree | strongly agree |
| strongly agree | strongly agree | strongly agree | strongly agree | strongly agree | strongly agree | strongly agree | strongly agree | strongly agree | strongly agree | strongly agree | strongly agree | strongly agree |
| strongly agree | strongly agree | strongly agree | strongly agree | strongly agree | strongly agree | strongly agree | strongly agree | strongly agree | strongly agree | strongly agree | strongly agree | strongly agree |
| strongly agree | strongly agree | strongly agree | strongly agree | strongly agree | strongly agree | strongly agree | strongly agree | strongly agree | strongly agree | strongly agree | strongly agree | strongly agree |
| strongly agree | strongly agree | strongly agree | strongly agree | strongly agree | strongly agree | strongly agree | strongly agree | strongly agree | strongly agree | strongly agree | strongly agree | strongly agree |
| strongly agree | strongly agree | strongly agree | strongly agree | strongly agree | strongly agree | strongly agree | strongly agree | strongly agree | strongly agree | strongly agree | strongly agree | strongly agree |
| strongly agree | strongly agree | strongly agree | strongly agree | strongly agree | strongly agree | strongly agree | strongly agree | strongly agree | strongly agree | strongly agree | strongly agree | strongly agree |
| strongly agree | strongly agree | strongly agree | strongly agree | strongly agree | strongly agree | strongly agree | strongly agree | strongly agree | strongly agree | strongly agree | strongly agree | strongly agree |
| strongly agree | strongly agree | strongly agree | strongly agree | strongly agree | strongly agree | strongly agree | strongly agree | strongly agree | strongly agree | strongly agree | strongly agree | strongly agree |
| strongly agree | strongly agree | strongly agree | strongly agree | strongly agree | strongly agree | strongly agree | strongly agree | strongly agree | strongly agree | strongly agree | strongly agree | strongly agree |
| strongly agree | strongly agree | strongly agree | strongly agree | strongly agree | strongly agree | strongly agree | strongly agree | strongly agree | strongly agree | strongly agree | strongly agree | strongly agree |
| strongly agree | strongly agree | strongly agree | strongly agree | strongly agree | strongly agree | strongly agree | strongly agree | strongly agree | strongly agree | strongly agree | strongly agree | strongly agree |
| strongly agree | strongly agree | strongly agree | strongly agree | strongly agree | strongly agree | strongly agree | strongly agree | strongly agree | strongly agree | strongly agree | strongly agree | strongly agree |
| strongly agree | strongly agree | strongly agree | strongly agree | strongly agree | strongly agree | strongly agree | strongly agree | strongly agree | strongly agree | strongly agree | strongly agree | strongly agree |
| strongly agree | strongly agree | strongly agree | strongly agree | strongly agree | strongly agree | strongly agree | strongly agree | strongly agree | strongly agree | strongly agree | strongly agree | strongly agree |
| strongly agree | strongly agree | strongly agree | strongly agree | strongly agree | strongly agree | strongly agree | strongly agree | strongly agree | strongly agree | strongly agree | strongly agree | strongly agree |
| strongly agree | strongly agree | strongly agree | strongly agree | strongly agree | strongly agree | strongly agree | strongly agree | strongly agree | strongly agree | strongly agree | strongly agree | strongly agree |
| strongly agree | strongly agree | strongly agree | strongly agree | strongly agree | strongly agree | strongly agree | strongly agree | strongly agree | strongly agree | strongly agree | strongly agree | strongly agree |
| strongly agree | strongly agree | strongly agree | strongly agree | strongly agree | strongly agree | strongly agree | strongly agree | strongly agree | strongly agree | strongly agree | strongly agree | strongly agree |
| strongly agree | strongly agree | strongly agree | strongly agree | strongly agree | strongly agree | strongly agree | strongly agree | strongly agree | strongly agree | strongly agree | strongly agree | strongly agree |
| strongly agree | strongly agree | strongly agree | strongly agree | strongly agree | strongly agree | strongly agree | strongly agree | strongly agree | strongly agree | strongly agree | strongly agree | strongly agree |
| strongly agree | strongly agree | strongly agree | strongly agree | strongly agree | strongly agree | strongly agree | strongly agree | strongly agree | strongly agree | strongly agree | strongly agree | strongly agree |
| strongly agree | strongly agree | strongly agree | strongly agree | strongly agree | strongly agree | strongly agree | strongly agree | strongly agree | strongly agree | strongly agree | strongly agree | strongly agree |
| strongly agree | strongly agree | strongly agree | strongly agree | strongly agree | strongly agree | strongly agree | strongly agree | strongly agree | strongly agree | strongly agree | strongly agree | strongly agree |
| strongly agree | strongly agree | strongly agree | strongly agree | strongly agree | strongly agree | strongly agree | strongly agree | strongly agree | strongly agree | strongly agree | strongly agree | strongly agree |
| strongly agree | strongly agree | strongly agree | strongly agree | strongly agree | strongly agree | strongly agree | strongly agree | strongly agree | strongly agree | strongly agree | strongly agree | strongly agree |
| disagree | disagree | disagree | disagree | disagree | disagree | strongly agree | disagree | strongly agree | strongly agree | disagree | strongly agree | strongly agree |
| strongly disagree | strongly disagree | strongly disagree | strongly disagree | strongly disagree | strongly disagree | strongly agree | strongly disagree | strongly agree | strongly agree | strongly disagree | strongly agree | strongly agree |
| disagree | disagree | disagree | disagree | disagree | disagree | strongly agree | disagree | strongly agree | strongly agree | disagree | strongly agree | strongly agree |
| disagree | disagree | disagree | disagree | disagree | disagree | strongly agree | disagree | strongly agree | strongly agree | disagree | strongly agree | strongly agree |
| disagree | disagree | disagree | disagree | disagree | disagree | strongly agree | disagree | strongly agree | strongly agree | disagree | strongly agree | strongly agree |
| disagree | disagree | disagree | disagree | disagree | disagree | strongly agree | disagree | strongly agree | strongly agree | disagree | strongly agree | strongly agree |
| strongly disagree | strongly disagree | strongly disagree | strongly disagree | strongly disagree | strongly disagree | strongly agree | strongly disagree | strongly agree | strongly agree | strongly disagree | strongly agree | strongly agree |
| disagree | disagree | disagree | disagree | disagree | disagree | strongly agree | disagree | strongly agree | strongly agree | disagree | strongly agree | strongly agree |
| strongly disagree | strongly disagree | strongly disagree | strongly disagree | strongly disagree | strongly disagree | strongly agree | strongly disagree | strongly agree | strongly agree | strongly disagree | strongly agree | strongly agree |
| Neutral | Neutral | Neutral | Neutral | Neutral | Neutral | strongly agree | Neutral | strongly agree | strongly agree | Neutral | strongly agree | strongly agree |
| disagree | disagree | disagree | disagree | disagree | disagree | strongly agree | disagree | strongly agree | strongly agree | disagree | strongly agree | strongly agree |
| disagree | disagree | disagree | disagree | disagree | disagree | strongly agree | disagree | strongly agree | strongly agree | disagree | strongly agree | strongly agree |
| disagree | disagree | disagree | disagree | disagree | disagree | strongly agree | disagree | strongly agree | strongly agree | disagree | strongly agree | strongly agree |
| disagree | disagree | disagree | disagree | disagree | disagree | disagree | disagree | disagree | disagree | disagree | disagree | disagree |
| disagree | disagree | disagree | disagree | disagree | disagree | disagree | disagree | disagree | disagree | disagree | disagree | disagree |
| disagree | disagree | disagree | disagree | disagree | disagree | disagree | disagree | disagree | disagree | disagree | disagree | disagree |
| disagree | disagree | disagree | disagree | disagree | disagree | disagree | disagree | disagree | disagree | disagree | disagree | disagree |
| strongly disagree | strongly disagree | strongly disagree | strongly disagree | strongly disagree | strongly disagree | strongly disagree | strongly disagree | strongly disagree | strongly disagree | strongly disagree | strongly disagree | strongly disagree |
| disagree | disagree | disagree | disagree | disagree | disagree | disagree | disagree | disagree | disagree | disagree | disagree | disagree |
| disagree | disagree | disagree | disagree | disagree | disagree | disagree | disagree | disagree | disagree | disagree | disagree | disagree |
| strongly disagree | strongly disagree | strongly disagree | strongly disagree | strongly disagree | strongly disagree | strongly disagree | strongly disagree | strongly disagree | strongly disagree | strongly disagree | strongly disagree | strongly disagree |
| strongly disagree | strongly disagree | strongly disagree | strongly disagree | strongly disagree | strongly disagree | strongly disagree | strongly disagree | strongly disagree | strongly disagree | strongly disagree | strongly disagree | strongly disagree |
| Neutral | Neutral | Neutral | Neutral | Neutral | Neutral | Neutral | Neutral | Neutral | Neutral | Neutral | Neutral | Neutral |
| disagree | disagree | disagree | disagree | disagree | disagree | disagree | disagree | disagree | disagree | disagree | disagree | disagree |
| strongly disagree | strongly disagree | strongly disagree | strongly disagree | strongly disagree | strongly disagree | strongly disagree | strongly disagree | strongly disagree | strongly disagree | strongly disagree | strongly disagree | strongly disagree |
| disagree | disagree | disagree | disagree | disagree | disagree | disagree | disagree | disagree | disagree | disagree | disagree | disagree |
| strongly disagree | strongly disagree | strongly disagree | strongly disagree | strongly disagree | strongly disagree | strongly disagree | strongly disagree | strongly disagree | strongly disagree | strongly disagree | strongly disagree | strongly disagree |
| disagree | disagree | disagree | disagree | disagree | disagree | disagree | disagree | disagree | disagree | disagree | disagree | disagree |
| strongly disagree | strongly disagree | strongly disagree | strongly disagree | strongly disagree | strongly disagree | strongly disagree | strongly disagree | strongly disagree | strongly disagree | strongly disagree | strongly disagree | strongly disagree |
| disagree | disagree | disagree | disagree | disagree | disagree | disagree | disagree | disagree | disagree | disagree | disagree | disagree |
| disagree | disagree | disagree | disagree | disagree | disagree | disagree | disagree | disagree | disagree | disagree | disagree | disagree |
| disagree | disagree | disagree | disagree | disagree | disagree | disagree | disagree | disagree | disagree | disagree | disagree | disagree |
| disagree | disagree | disagree | disagree | disagree | disagree | disagree | disagree | disagree | disagree | disagree | disagree | disagree |
| disagree | disagree | disagree | disagree | disagree | disagree | disagree | disagree | disagree | disagree | disagree | disagree | disagree |
| Neutral | Neutral | Neutral | Neutral | Neutral | Neutral | Neutral | Neutral | Neutral | Neutral | Neutral | Neutral | Neutral |
| disagree | disagree | disagree | disagree | disagree | disagree | disagree | disagree | disagree | disagree | disagree | disagree | disagree |
| Neutral | Neutral | Neutral | Neutral | Neutral | Neutral | Neutral | Neutral | Neutral | Neutral | Neutral | Neutral | Neutral |
| disagree | disagree | disagree | disagree | disagree | disagree | disagree | disagree | disagree | disagree | disagree | disagree | disagree |
| Neutral | Neutral | Neutral | Neutral | Neutral | Neutral | Neutral | Neutral | Neutral | Neutral | Neutral | Neutral | Neutral |
| disagree | disagree | disagree | disagree | disagree | disagree | disagree | disagree | disagree | disagree | disagree | disagree | disagree |
| disagree | disagree | disagree | disagree | disagree | disagree | disagree | disagree | disagree | disagree | disagree | disagree | disagree |
| disagree | disagree | disagree | disagree | disagree | disagree | disagree | disagree | disagree | disagree | disagree | disagree | disagree |
| disagree | disagree | disagree | disagree | disagree | disagree | disagree | disagree | disagree | disagree | disagree | disagree | disagree |
| disagree | disagree | disagree | disagree | disagree | disagree | disagree | disagree | disagree | disagree | disagree | disagree | disagree |
| Neutral | Neutral | Neutral | Neutral | Neutral | Neutral | Neutral | Neutral | Neutral | Neutral | Neutral | Neutral | Neutral |
| disagree | disagree | disagree | disagree | disagree | disagree | disagree | disagree | disagree | disagree | disagree | disagree | disagree |
| Neutral | Neutral | Neutral | Neutral | Neutral | Neutral | Neutral | Neutral | Neutral | Neutral | Neutral | Neutral | Neutral |
| disagree | disagree | disagree | disagree | disagree | disagree | disagree | disagree | disagree | disagree | disagree | disagree | disagree |
| Neutral | Neutral | Neutral | Neutral | Neutral | Neutral | Neutral | Neutral | Neutral | Neutral | Neutral | Neutral | Neutral |
| disagree | disagree | disagree | disagree | disagree | disagree | disagree | disagree | disagree | disagree | disagree | disagree | disagree |
| Neutral | Neutral | Neutral | Neutral | Neutral | Neutral | Neutral | Neutral | Neutral | Neutral | Neutral | Neutral | Neutral |
| Neutral | Neutral | Neutral | Neutral | Neutral | Neutral | Neutral | Neutral | Neutral | Neutral | Neutral | Neutral | Neutral |
| disagree | disagree | disagree | disagree | disagree | disagree | disagree | disagree | disagree | disagree | disagree | disagree | disagree |
| Neutral | Neutral | Neutral | Neutral | Neutral | Neutral | Neutral | Neutral | Neutral | Neutral | Neutral | Neutral | Neutral |
| disagree | disagree | disagree | disagree | disagree | disagree | disagree | disagree | disagree | disagree | disagree | disagree | disagree |
| Neutral | Neutral | Neutral | Neutral | Neutral | Neutral | Neutral | Neutral | Neutral | Neutral | Neutral | Neutral | Neutral |
| disagree | disagree | disagree | disagree | disagree | disagree | disagree | disagree | disagree | disagree | disagree | disagree | disagree |
| disagree | disagree | disagree | disagree | disagree | disagree | disagree | disagree | disagree | disagree | disagree | disagree | disagree |
| disagree | disagree | disagree | disagree | disagree | disagree | disagree | disagree | disagree | disagree | disagree | disagree | disagree |
| disagree | disagree | disagree | disagree | disagree | disagree | disagree | disagree | disagree | disagree | disagree | disagree | disagree |
| disagree | disagree | disagree | disagree | disagree | disagree | disagree | disagree | disagree | disagree | disagree | disagree | disagree |
| disagree | disagree | disagree | disagree | disagree | disagree | disagree | disagree | disagree | disagree | disagree | disagree | disagree |
| disagree | disagree | disagree | disagree | disagree | disagree | disagree | disagree | disagree | disagree | disagree | disagree | disagree |
| disagree | disagree | disagree | disagree | disagree | disagree | disagree | disagree | disagree | disagree | disagree | disagree | disagree |
| disagree | disagree | disagree | disagree | disagree | disagree | disagree | disagree | disagree | disagree | disagree | disagree | disagree |
| disagree | disagree | disagree | disagree | disagree | disagree | disagree | disagree | disagree | disagree | disagree | disagree | disagree |
| disagree | disagree | disagree | disagree | disagree | disagree | disagree | disagree | disagree | disagree | disagree | disagree | disagree |
| disagree | disagree | disagree | disagree | disagree | disagree | disagree | disagree | disagree | disagree | disagree | disagree | disagree |
| Neutral | Neutral | Neutral | Neutral | Neutral | Neutral | Neutral | Neutral | Neutral | Neutral | Neutral | Neutral | Neutral |
| disagree | disagree | disagree | disagree | disagree | disagree | disagree | disagree | disagree | disagree | disagree | disagree | disagree |
| disagree | disagree | disagree | disagree | disagree | disagree | disagree | disagree | disagree | disagree | disagree | disagree | disagree |
| disagree | disagree | disagree | disagree | disagree | disagree | disagree | disagree | disagree | disagree | disagree | disagree | disagree |
| disagree | disagree | disagree | disagree | disagree | disagree | disagree | disagree | disagree | disagree | disagree | disagree | disagree |
| disagree | disagree | disagree | disagree | disagree | disagree | disagree | disagree | disagree | disagree | disagree | disagree | disagree |
| Neutral | Neutral | Neutral | Neutral | Neutral | Neutral | Neutral | Neutral | Neutral | Neutral | Neutral | Neutral | Neutral |
| disagree | disagree | disagree | disagree | disagree | disagree | disagree | disagree | disagree | disagree | disagree | disagree | disagree |
| Neutral | Neutral | Neutral | Neutral | Neutral | Neutral | Neutral | Neutral | Neutral | Neutral | Neutral | Neutral | Neutral |

| market3 | market4 | market5 | perfprofitable | perturnover | perunemploy | average_gov.t | average_bus | average_managment | average_finacial | average_market | performance | PRE_1 |
| --- | --- | --- | --- | --- | --- | --- | --- | --- | --- | --- | --- | --- |
| strongly disagree | strongly disagree | strongly disagree | strongly disagree | strongly disagree | strongly disagree | 5 | 2 | 3 | 3 | 1 | 3 | 2.80587 |
| strongly disagree | strongly disagree | strongly disagree | strongly disagree | strongly disagree | strongly disagree | 5 | 2 | 3 | 3 | 1 | 3 | 2.80587 |
| strongly disagree | strongly disagree | strongly disagree | strongly disagree | strongly disagree | strongly disagree | 5 | 2 | 3 | 3 | 1 | 3 | 2.80587 |
| strongly disagree | strongly disagree | strongly disagree | strongly disagree | strongly disagree | strongly disagree | 5 | 2 | 2 | 3 | 1 | 3 | 2.58011 |
| strongly disagree | strongly disagree | strongly disagree | strongly disagree | strongly disagree | strongly disagree | 5 | 2 | 2 | 3 | 1 | 3 | 2.2473 |
| strongly disagree | strongly disagree | strongly disagree | strongly disagree | strongly disagree | strongly disagree | 5 | 2 | 2 | 3 | 1 | 3 | 2.58011 |
| strongly disagree | strongly disagree | strongly disagree | strongly disagree | strongly disagree | strongly disagree | 5 | 2 | 2 | 3 | 1 | 3 | 2.58011 |
| strongly disagree | strongly disagree | strongly disagree | strongly disagree | strongly disagree | strongly disagree | 5 | 2 | 2 | 3 | 1 | 3 | 2.58011 |
| strongly disagree | strongly disagree | strongly disagree | strongly disagree | strongly disagree | strongly disagree | 5 | 2 | 2 | 3 | 1 | 3 | 2.58011 |
| strongly disagree | strongly disagree | strongly disagree | strongly disagree | strongly disagree | strongly disagree | 5 | 2 | 2 | 3 | 1 | 3 | 2.58011 |
| strongly disagree | strongly disagree | strongly disagree | strongly disagree | strongly disagree | strongly disagree | 5 | 2 | 1 | 3 | 1 | 3 | 2.3995 |
| strongly disagree | strongly disagree | strongly disagree | strongly disagree | strongly disagree | strongly disagree | 5 | 2 | 1 | 3 | 1 | 3 | 2.3995 |
| strongly disagree | strongly disagree | strongly disagree | strongly disagree | strongly disagree | strongly disagree | 5 | 2 | 1 | 3 | 1 | 3 | 2.3995 |
| strongly disagree | strongly disagree | strongly disagree | strongly disagree | strongly disagree | strongly disagree | 5 | 2 | 1 | 3 | 1 | 3 | 2.3995 |
| strongly disagree | strongly disagree | strongly disagree | strongly disagree | strongly disagree | strongly disagree | 5 | 2 | 1 | 3 | 1 | 3 | 2.3995 |
| strongly disagree | strongly disagree | strongly disagree | strongly disagree | strongly disagree | strongly disagree | 5 | 3 | 1 | 3 | 2 | 3 | 2.57677 |
| strongly disagree | strongly disagree | strongly disagree | strongly disagree | strongly disagree | strongly disagree | 5 | 3 | 1 | 3 | 2 | 3 | 2.57677 |
| strongly disagree | strongly disagree | strongly disagree | strongly disagree | strongly disagree | strongly disagree | 5 | 3 | 1 | 3 | 2 | 3 | 2.57677 |
| strongly disagree | strongly disagree | strongly disagree | strongly disagree | strongly disagree | strongly disagree | 5 | 3 | 1 | 3 | 2 | 2 | 2.57677 |
| strongly agree | strongly disagree | strongly disagree | strongly disagree | strongly disagree | strongly disagree | 5 | 3 | 1 | 3 | 3 | 2 | 2.75404 |
| strongly agree | strongly disagree | strongly disagree | strongly disagree | strongly disagree | strongly disagree | 5 | 3 | 1 | 3 | 3 | 2 | 2.75404 |
| strongly agree | strongly disagree | strongly disagree | strongly disagree | strongly disagree | strongly disagree | 5 | 3 | 3 | 3 | 3 | 2 | 3.29587 |
| strongly agree | strongly disagree | strongly disagree | strongly disagree | strongly disagree | strongly disagree | 5 | 3 | 3 | 3 | 3 | 2 | 3.29587 |
| strongly agree | strongly disagree | strongly disagree | strongly disagree | strongly disagree | strongly disagree | 5 | 3 | 3 | 3 | 3 | 2 | 3.29587 |
| strongly agree | strongly disagree | strongly disagree | strongly disagree | strongly disagree | strongly disagree | 5 | 3 | 3 | 3 | 3 | 2 | 3.29587 |
| strongly agree | strongly disagree | strongly disagree | strongly disagree | strongly disagree | strongly disagree | 5 | 3 | 3 | 3 | 3 | 2 | 3.47314 |
| strongly agree | strongly disagree | strongly disagree | strongly disagree | strongly disagree | strongly disagree | 5 | 3 | 3 | 3 | 3 | 2 | 3.47314 |
| strongly agree | strongly disagree | strongly disagree | strongly disagree | strongly disagree | strongly disagree | 5 | 4 | 3 | 5 | 3 | 2 | 4.13875 |
| strongly agree | strongly disagree | strongly disagree | strongly agree | strongly disagree | strongly disagree | 5 | 4 | 3 | 5 | 3 | 2 | 4.13875 |
| strongly agree | strongly disagree | strongly disagree | strongly agree | strongly disagree | strongly disagree | 5 | 4 | 3 | 5 | 3 | 2 | 4.13875 |
| strongly agree | strongly agree | strongly disagree | strongly agree | strongly disagree | strongly disagree | 5 | 4 | 3 | 5 | 4 | 4 | 4.31603 |
| strongly agree | strongly agree | strongly disagree | strongly agree | strongly disagree | strongly disagree | 5 | 4 | 3 | 5 | 4 | 4 | 4.31603 |
| strongly agree | strongly agree | strongly disagree | strongly agree | strongly disagree | strongly disagree | 5 | 4 | 3 | 5 | 4 | 4 | 4.31603 |
| strongly agree | strongly agree | strongly disagree | strongly agree | strongly disagree | strongly disagree | 5 | 4 | 3 | 5 | 4 | 4 | 4.31603 |
| strongly agree | strongly agree | strongly disagree | strongly agree | strongly agree | strongly agree | 5 | 5 | 5 | 5 | 4 | 5 | 4.67725 |
| strongly agree | strongly agree | strongly disagree | strongly agree | strongly agree | strongly agree | 5 | 5 | 5 | 5 | 4 | 5 | 4.67725 |
| strongly agree | strongly agree | strongly agree | strongly agree | strongly agree | strongly agree | 5 | 5 | 5 | 5 | 5 | 5 | 4.85452 |
| strongly agree | strongly agree | strongly agree | strongly agree | strongly agree | strongly agree | 5 | 5 | 5 | 5 | 5 | 5 | 4.85452 |
| strongly agree | strongly agree | strongly agree | strongly agree | strongly agree | strongly agree | 5 | 5 | 5 | 5 | 5 | 5 | 4.85452 |
| strongly agree | strongly agree | strongly agree | strongly agree | strongly disagree | strongly disagree | 5 | 5 | 4 | 5 | 5 | 5 | 4.67391 |
| strongly agree | strongly agree | strongly agree | strongly agree | strongly disagree | strongly disagree | 5 | 5 | 4 | 5 | 5 | 5 | 4.67391 |
| strongly agree | strongly agree | strongly agree | strongly agree | strongly disagree | strongly disagree | 5 | 5 | 4 | 5 | 5 | 5 | 4.67391 |
| strongly agree | strongly agree | strongly agree | strongly agree | strongly disagree | strongly disagree | 5 | 5 | 5 | 5 | 5 | 5 | 4.85452 |
| strongly agree | strongly agree | strongly agree | strongly agree | strongly disagree | strongly disagree | 5 | 5 | 5 | 5 | 5 | 5 | 4.85452 |
| strongly agree | strongly agree | strongly agree | strongly agree | strongly disagree | strongly disagree | 5 | 5 | 5 | 5 | 5 | 5 | 4.85452 |
| strongly agree | strongly agree | strongly agree | strongly agree | strongly disagree | strongly disagree | 5 | 5 | 5 | 5 | 5 | 5 | 4.85452 |
| strongly agree | strongly agree | strongly agree | strongly agree | strongly disagree | strongly disagree | 5 | 5 | 5 | 5 | 5 | 5 | 4.85452 |
| strongly agree | strongly agree | strongly agree | strongly agree | strongly disagree | strongly disagree | 5 | 5 | 5 | 5 | 5 | 5 | 4.85452 |
| strongly agree | strongly agree | strongly agree | strongly agree | strongly disagree | strongly disagree | 5 | 5 | 5 | 5 | 5 | 5 | 4.85452 |
| strongly agree | strongly agree | strongly agree | strongly agree | strongly disagree | strongly disagree | 5 | 5 | 5 | 5 | 5 | 5 | 4.85452 |
| strongly agree | strongly agree | strongly agree | strongly agree | strongly disagree | strongly disagree | 5 | 5 | 5 | 5 | 5 | 5 | 4.85452 |
| strongly agree | strongly agree | strongly agree | strongly agree | strongly disagree | strongly disagree | 5 | 5 | 5 | 5 | 5 | 5 | 4.85452 |
| strongly agree | strongly agree | strongly agree | strongly agree | strongly disagree | strongly disagree | 5 | 5 | 5 | 5 | 5 | 5 | 4.85452 |
| strongly agree | strongly agree | strongly agree | strongly agree | strongly disagree | strongly disagree | 5 | 5 | 5 | 5 | 5 | 5 | 4.85452 |
| strongly agree | strongly agree | strongly agree | strongly agree | strongly disagree | strongly disagree | 5 | 5 | 5 | 5 | 5 | 5 | 4.85452 |
| strongly agree | strongly agree | strongly agree | strongly agree | strongly disagree | strongly disagree | 5 | 5 | 5 | 5 | 5 | 5 | 4.85452 |
| strongly agree | strongly agree | strongly agree | strongly agree | strongly disagree | strongly disagree | 5 | 5 | 5 | 5 | 5 | 5 | 4.85452 |
| strongly agree | strongly agree | strongly agree | strongly agree | strongly disagree | strongly disagree | 5 | 5 | 5 | 5 | 5 | 5 | 4.85452 |
| strongly agree | strongly agree | strongly agree | strongly agree | strongly disagree | strongly disagree | 5 | 5 | 5 | 5 | 5 | 5 | 4.85452 |
| strongly agree | strongly agree | strongly agree | strongly agree | strongly disagree | strongly disagree | 5 | 5 | 5 | 5 | 5 | 5 | 4.85452 |
| strongly agree | strongly agree | strongly agree | strongly agree | strongly disagree | strongly disagree | 5 | 5 | 5 | 5 | 5 | 5 | 4.85452 |
| strongly agree | strongly agree | strongly agree | strongly agree | strongly disagree | strongly disagree | 5 | 5 | 5 | 5 | 5 | 5 | 4.85452 |
| strongly agree | strongly agree | strongly agree | strongly agree | strongly disagree | strongly disagree | 5 | 5 | 5 | 5 | 5 | 5 | 4.85452 |
| strongly agree | strongly agree | strongly agree | strongly agree | strongly disagree | strongly disagree | 5 | 5 | 5 | 5 | 5 | 5 | 4.85452 |
| strongly agree | strongly agree | strongly agree | strongly agree | strongly disagree | strongly agree | 5 | 5 | 5 | 5 | 5 | 5 | 4.85452 |
| strongly agree | strongly agree | strongly agree | strongly agree | strongly disagree | strongly agree | 5 | 5 | 5 | 5 | 5 | 5 | 4.85452 |
| strongly agree | strongly agree | strongly agree | strongly agree | strongly disagree | strongly agree | 5 | 5 | 5 | 5 | 5 | 5 | 4.85452 |
| strongly agree | strongly agree | strongly agree | strongly agree | strongly agree | strongly agree | 5 | 5 | 5 | 5 | 5 | 5 | 4.85452 |
| strongly agree | strongly agree | strongly agree | strongly agree | strongly agree | strongly agree | 5 | 5 | 5 | 5 | 5 | 5 | 4.85452 |
| strongly agree | strongly agree | strongly agree | strongly agree | strongly agree | strongly agree | 5 | 5 | 5 | 5 | 5 | 5 | 4.85452 |
| strongly agree | strongly agree | strongly agree | strongly agree | strongly agree | strongly agree | 5 | 5 | 5 | 5 | 5 | 5 | 4.85452 |
| strongly agree | strongly agree | strongly agree | strongly agree | strongly agree | strongly agree | 5 | 5 | 5 | 5 | 5 | 5 | 4.85452 |
| strongly agree | strongly agree | strongly agree | strongly agree | strongly agree | strongly agree | 5 | 5 | 5 | 5 | 5 | 5 | 4.85452 |
| strongly agree | strongly agree | strongly agree | strongly agree | strongly agree | strongly agree | 5 | 5 | 5 | 5 | 5 | 5 | 4.85452 |
| strongly agree | strongly agree | strongly agree | strongly agree | strongly agree | strongly agree | 5 | 5 | 5 | 5 | 5 | 5 | 4.85452 |
| strongly agree | strongly agree | strongly agree | strongly agree | strongly agree | strongly agree | 5 | 5 | 5 | 5 | 5 | 5 | 4.85452 |
| strongly agree | strongly agree | strongly agree | strongly agree | strongly agree | strongly agree | 5 | 5 | 5 | 5 | 5 | 5 | 4.85452 |
| strongly agree | strongly agree | strongly agree | strongly agree | strongly agree | strongly agree | 5 | 5 | 5 | 5 | 5 | 5 | 4.85452 |
| strongly agree | strongly agree | strongly agree | strongly agree | strongly agree | strongly agree | 5 | 5 | 5 | 5 | 5 | 5 | 4.85452 |
| strongly agree | strongly agree | strongly agree | strongly agree | strongly agree | strongly agree | 5 | 5 | 5 | 5 | 5 | 5 | 4.85452 |
| strongly agree | strongly agree | strongly agree | strongly agree | strongly agree | strongly agree | 5 | 5 | 5 | 5 | 5 | 5 | 4.85452 |
| strongly agree | strongly agree | strongly agree | strongly agree | strongly agree | strongly agree | 5 | 5 | 5 | 5 | 5 | 5 | 4.85452 |
| strongly agree | strongly agree | strongly agree | strongly agree | strongly agree | strongly agree | 5 | 5 | 5 | 5 | 5 | 5 | 4.85452 |
| strongly agree | strongly agree | strongly agree | strongly agree | strongly agree | strongly agree | 5 | 5 | 5 | 5 | 5 | 5 | 4.85452 |
| strongly agree | strongly agree | strongly agree | strongly agree | strongly agree | strongly agree | 5 | 5 | 5 | 5 | 5 | 5 | 4.85452 |
| strongly agree | strongly agree | strongly agree | strongly agree | strongly agree | strongly agree | 5 | 5 | 5 | 5 | 5 | 5 | 4.85452 |
| strongly agree | strongly agree | strongly agree | strongly agree | strongly agree | strongly agree | 5 | 5 | 5 | 5 | 5 | 4 | 4.85452 |
| strongly agree | strongly agree | strongly agree | strongly agree | strongly agree | strongly agree | 5 | 5 | 5 | 5 | 5 | 4 | 4.85452 |
| strongly agree | strongly agree | strongly agree | strongly agree | strongly agree | strongly agree | 5 | 5 | 5 | 5 | 5 | 4 | 4.85452 |
| strongly agree | strongly agree | strongly agree | strongly agree | strongly agree | strongly agree | 5 | 5 | 5 | 5 | 5 | 4 | 4.85452 |
| strongly agree | strongly agree | strongly agree | strongly agree | strongly agree | strongly agree | 5 | 5 | 5 | 5 | 5 | 4 | 4.85452 |
| strongly agree | strongly agree | strongly agree | strongly agree | strongly agree | strongly agree | 5 | 5 | 5 | 5 | 5 | 4 | 4.85452 |
| strongly agree | strongly agree | strongly agree | strongly agree | strongly agree | strongly agree | 5 | 5 | 5 | 5 | 5 | 4 | 4.85452 |
| strongly agree | strongly agree | strongly agree | strongly agree | strongly agree | strongly agree | 5 | 5 | 5 | 5 | 5 | 4 | 4.85452 |
| strongly agree | strongly agree | strongly agree | strongly agree | strongly agree | strongly agree | 5 | 5 | 5 | 5 | 5 | 4 | 4.85452 |
| strongly agree | strongly agree | strongly agree | strongly agree | strongly agree | strongly agree | 5 | 5 | 5 | 5 | 5 | 4 | 4.85452 |
| strongly agree | strongly agree | strongly agree | strongly agree | strongly agree | strongly agree | 5 | 5 | 5 | 5 | 5 | 4 | 4.85452 |
| strongly agree | strongly agree | strongly agree | strongly agree | strongly agree | strongly agree | 5 | 5 | 5 | 5 | 5 | 5 | 4.85452 |
| strongly agree | strongly agree | strongly agree | strongly agree | strongly agree | strongly agree | 5 | 5 | 5 | 5 | 5 | 5 | 4.85452 |
| strongly agree | strongly agree | strongly agree | strongly agree | strongly agree | strongly agree | 5 | 5 | 5 | 5 | 5 | 5 | 4.85452 |
| strongly agree | strongly agree | strongly agree | strongly agree | strongly agree | strongly agree | 5 | 5 | 5 | 5 | 5 | 5 | 4.85452 |
| strongly agree | strongly agree | strongly agree | strongly agree | strongly agree | strongly agree | 5 | 5 | 5 | 5 | 5 | 5 | 4.85452 |
| strongly agree | strongly agree | strongly agree | strongly agree | strongly agree | strongly agree | 5 | 5 | 5 | 5 | 5 | 5 | 4.85452 |
| strongly agree | strongly agree | strongly agree | strongly agree | strongly agree | strongly agree | 5 | 5 | 5 | 5 | 5 | 5 | 4.85452 |
| strongly agree | strongly agree | strongly agree | strongly agree | strongly agree | strongly agree | 5 | 5 | 5 | 5 | 5 | 5 | 4.85452 |
| strongly agree | strongly agree | strongly agree | strongly agree | strongly agree | strongly agree | 5 | 5 | 5 | 5 | 5 | 5 | 4.85452 |
| strongly agree | strongly agree | strongly agree | strongly agree | strongly agree | strongly agree | 5 | 5 | 5 | 5 | 5 | 5 | 4.85452 |
| strongly agree | strongly agree | strongly agree | strongly agree | strongly agree | strongly agree | 5 | 5 | 5 | 5 | 5 | 5 | 4.85452 |
| strongly agree | strongly agree | strongly agree | strongly agree | strongly agree | strongly agree | 5 | 5 | 5 | 5 | 5 | 5 | 4.85452 |
| strongly agree | strongly agree | strongly agree | strongly agree | strongly agree | strongly agree | 5 | 5 | 5 | 5 | 5 | 5 | 4.85452 |
| strongly agree | strongly agree | strongly agree | strongly agree | strongly agree | strongly agree | 5 | 5 | 5 | 5 | 5 | 5 | 4.85452 |
| strongly agree | strongly agree | strongly agree | strongly agree | strongly agree | strongly agree | 5 | 5 | 5 | 5 | 5 | 5 | 4.85452 |
| strongly agree | strongly agree | strongly agree | strongly agree | strongly agree | strongly agree | 5 | 5 | 5 | 5 | 5 | 5 | 4.85452 |
| strongly agree | strongly agree | strongly agree | strongly agree | strongly agree | strongly agree | 5 | 5 | 5 | 5 | 5 | 5 | 4.85452 |
| strongly agree | strongly agree | strongly agree | strongly agree | strongly agree | strongly agree | 5 | 5 | 5 | 5 | 5 | 5 | 4.85452 |
| strongly agree | strongly agree | strongly agree | strongly agree | strongly agree | strongly agree | 5 | 5 | 5 | 5 | 5 | 5 | 4.85452 |
| strongly agree | strongly agree | strongly agree | strongly agree | strongly agree | strongly agree | 5 | 5 | 5 | 5 | 5 | 5 | 4.85452 |
| strongly agree | strongly agree | strongly agree | strongly agree | strongly agree | strongly agree | 5 | 5 | 5 | 5 | 5 | 5 | 4.85452 |
| strongly agree | strongly agree | strongly agree | strongly agree | strongly agree | strongly agree | 5 | 5 | 5 | 5 | 5 | 5 | 4.85452 |
| strongly agree | strongly agree | strongly agree | strongly agree | strongly agree | strongly agree | 5 | 5 | 5 | 5 | 5 | 5 | 4.85452 |
| strongly agree | strongly agree | strongly agree | strongly agree | strongly agree | strongly agree | 5 | 5 | 5 | 5 | 5 | 5 | 4.85452 |
| strongly agree | strongly agree | strongly agree | strongly agree | strongly agree | strongly agree | 5 | 5 | 5 | 5 | 5 | 5 | 4.85452 |
| strongly agree | strongly agree | strongly agree | strongly agree | strongly agree | strongly agree | 5 | 5 | 5 | 5 | 5 | 5 | 4.85452 |
| strongly agree | strongly agree | strongly agree | strongly agree | strongly agree | strongly agree | 5 | 5 | 5 | 5 | 5 | 5 | 4.85452 |
| strongly agree | strongly agree | strongly agree | strongly agree | strongly agree | strongly agree | 5 | 5 | 5 | 5 | 5 | 5 | 4.85452 |
| strongly agree | strongly agree | strongly agree | strongly agree | strongly agree | strongly agree | 5 | 5 | 5 | 5 | 5 | 5 | 4.85452 |
| strongly agree | strongly agree | strongly agree | strongly agree | strongly agree | strongly agree | 5 | 5 | 5 | 5 | 5 | 5 | 4.85452 |
| strongly agree | strongly agree | strongly agree | strongly agree | strongly agree | strongly agree | 5 | 5 | 5 | 5 | 5 | 5 | 4.85452 |
| strongly agree | strongly agree | strongly agree | strongly agree | strongly agree | strongly agree | 5 | 5 | 5 | 5 | 5 | 5 | 4.85452 |
| strongly agree | strongly agree | strongly agree | strongly agree | strongly agree | strongly agree | 5 | 5 | 5 | 5 | 5 | 5 | 4.85452 |
| strongly agree | strongly agree | strongly agree | strongly agree | strongly agree | strongly agree | 5 | 5 | 5 | 5 | 5 | 5 | 4.85452 |
| strongly agree | strongly agree | strongly agree | strongly agree | strongly agree | strongly agree | 5 | 5 | 5 | 5 | 5 | 5 | 4.85452 |
| strongly agree | strongly agree | strongly agree | strongly agree | strongly agree | strongly agree | 5 | 5 | 5 | 5 | 5 | 5 | 4.85452 |
| strongly agree | strongly agree | strongly agree | strongly agree | strongly agree | strongly agree | 5 | 5 | 5 | 5 | 5 | 4 | 4.85452 |
| strongly agree | strongly agree | strongly agree | strongly agree | strongly agree | strongly agree | 5 | 5 | 5 | 5 | 5 | 4 | 4.85452 |
| strongly agree | strongly agree | strongly agree | strongly agree | strongly agree | strongly agree | 5 | 5 | 5 | 5 | 5 | 4 | 4.85452 |
| strongly agree | strongly agree | strongly agree | strongly agree | strongly agree | strongly agree | 5 | 5 | 5 | 5 | 5 | 4 | 4.85452 |
| strongly agree | strongly agree | strongly agree | strongly agree | strongly agree | strongly agree | 5 | 5 | 5 | 5 | 5 | 4 | 4.85452 |
| strongly agree | strongly agree | strongly agree | strongly agree | strongly agree | strongly agree | 5 | 5 | 5 | 5 | 5 | 4 | 4.85452 |
| strongly agree | strongly agree | strongly agree | strongly agree | strongly agree | strongly agree | 5 | 5 | 5 | 5 | 5 | 4 | 4.85452 |
| strongly agree | strongly agree | strongly agree | strongly agree | strongly agree | strongly agree | 5 | 5 | 5 | 5 | 5 | 4 | 4.85452 |
| strongly agree | strongly agree | strongly agree | strongly agree | strongly agree | strongly agree | 5 | 5 | 5 | 5 | 5 | 4 | 4.85452 |
| strongly agree | strongly agree | strongly agree | strongly agree | strongly agree | strongly agree | 5 | 5 | 5 | 5 | 5 | 5 | 4.85452 |
| strongly agree | strongly agree | strongly agree | strongly agree | strongly agree | strongly agree | 5 | 5 | 5 | 5 | 5 | 5 | 4.85452 |
| strongly agree | strongly agree | strongly agree | strongly agree | strongly agree | strongly agree | 5 | 5 | 5 | 5 | 5 | 5 | 4.85452 |
| strongly agree | strongly agree | strongly agree | strongly agree | strongly agree | strongly agree | 5 | 5 | 5 | 5 | 5 | 5 | 4.85452 |
| strongly agree | strongly agree | strongly agree | strongly agree | strongly agree | strongly agree | 5 | 5 | 5 | 5 | 5 | 5 | 4.85452 |
| strongly agree | strongly agree | strongly agree | strongly agree | strongly agree | strongly agree | 5 | 5 | 5 | 5 | 5 | 5 | 4.85452 |
| strongly agree | strongly agree | strongly agree | strongly agree | strongly agree | strongly agree | 5 | 5 | 5 | 5 | 5 | 5 | 4.85452 |
| strongly agree | strongly agree | strongly agree | strongly agree | strongly agree | strongly agree | 5 | 5 | 5 | 5 | 5 | 5 | 4.85452 |
| strongly agree | strongly agree | strongly agree | strongly agree | strongly agree | strongly agree | 5 | 5 | 5 | 5 | 5 | 5 | 4.85452 |
| strongly agree | strongly agree | strongly agree | strongly agree | strongly agree | strongly agree | 5 | 5 | 5 | 5 | 5 | 5 | 4.85452 |
| strongly agree | strongly agree | strongly agree | strongly agree | strongly agree | strongly agree | 5 | 5 | 5 | 5 | 5 | 5 | 4.85452 |
| strongly agree | strongly agree | strongly agree | strongly agree | strongly agree | strongly agree | 5 | 5 | 5 | 5 | 5 | 5 | 4.85452 |
| strongly agree | strongly agree | strongly agree | strongly agree | strongly agree | strongly agree | 5 | 5 | 5 | 5 | 5 | 5 | 4.85452 |
| strongly agree | strongly agree | strongly agree | strongly agree | strongly agree | strongly agree | 5 | 5 | 5 | 5 | 5 | 5 | 4.85452 |
| strongly agree | strongly agree | strongly agree | strongly agree | strongly agree | strongly agree | 5 | 5 | 5 | 5 | 5 | 5 | 4.85452 |
| strongly agree | strongly agree | strongly agree | strongly agree | strongly agree | strongly agree | 5 | 5 | 5 | 5 | 5 | 5 | 4.85452 |
| strongly agree | strongly agree | strongly agree | strongly agree | strongly agree | strongly agree | 5 | 5 | 5 | 5 | 5 | 5 | 4.85452 |
| strongly agree | strongly agree | strongly agree | strongly agree | strongly agree | strongly agree | 5 | 5 | 5 | 5 | 5 | 5 | 4.85452 |
| strongly agree | strongly agree | strongly agree | strongly agree | strongly agree | strongly agree | 5 | 5 | 5 | 5 | 5 | 5 | 4.85452 |
| strongly agree | strongly agree | strongly agree | strongly agree | strongly agree | strongly agree | 5 | 5 | 5 | 5 | 5 | 5 | 4.85452 |
| strongly agree | strongly agree | strongly agree | strongly agree | strongly agree | strongly agree | 5 | 5 | 5 | 5 | 5 | 5 | 4.85452 |
| strongly agree | strongly agree | strongly agree | strongly agree | strongly agree | strongly agree | 5 | 5 | 5 | 5 | 5 | 5 | 4.85452 |
| strongly agree | strongly agree | strongly agree | strongly agree | strongly agree | strongly agree | 5 | 5 | 5 | 5 | 5 | 5 | 4.85452 |
| strongly agree | strongly agree | strongly agree | strongly agree | strongly agree | strongly agree | 5 | 5 | 5 | 5 | 5 | 5 | 4.85452 |
| strongly agree | strongly agree | strongly agree | strongly agree | strongly agree | strongly agree | 5 | 5 | 5 | 5 | 5 | 5 | 4.85452 |
| strongly agree | strongly agree | strongly agree | strongly agree | strongly agree | strongly agree | 5 | 5 | 5 | 5 | 5 | 5 | 4.85452 |
| strongly agree | strongly agree | strongly agree | strongly agree | strongly agree | strongly agree | 5 | 5 | 5 | 5 | 5 | 5 | 4.85452 |
| strongly agree | strongly agree | strongly agree | strongly agree | strongly agree | strongly agree | 5 | 5 | 5 | 5 | 5 | 5 | 4.85452 |
| strongly agree | strongly agree | strongly agree | strongly agree | strongly agree | strongly agree | 5 | 5 | 5 | 5 | 5 | 5 | 4.85452 |
| strongly agree | strongly agree | strongly agree | strongly agree | strongly agree | strongly agree | 5 | 5 | 5 | 5 | 5 | 5 | 4.85452 |
| strongly agree | strongly agree | strongly agree | strongly agree | strongly agree | strongly agree | 5 | 5 | 5 | 5 | 5 | 5 | 4.85452 |
| strongly agree | strongly agree | strongly agree | strongly agree | strongly agree | strongly agree | 5 | 5 | 5 | 5 | 5 | 5 | 4.85452 |
| strongly agree | strongly agree | strongly agree | strongly agree | strongly agree | strongly agree | 5 | 5 | 5 | 5 | 5 | 5 | 4.85452 |
| strongly agree | strongly agree | strongly agree | strongly agree | strongly agree | strongly agree | 5 | 5 | 5 | 5 | 5 | 5 | 4.85452 |
| strongly agree | strongly agree | strongly agree | strongly agree | strongly agree | strongly agree | 5 | 5 | 5 | 5 | 5 | 5 | 4.85452 |
| strongly agree | strongly agree | strongly agree | strongly agree | strongly agree | strongly agree | 5 | 5 | 5 | 5 | 5 | 5 | 4.85452 |
| strongly agree | strongly agree | strongly agree | strongly agree | strongly agree | strongly agree | 5 | 5 | 5 | 5 | 5 | 5 | 4.85452 |
| strongly agree | strongly agree | strongly agree | strongly agree | strongly agree | strongly agree | 5 | 5 | 5 | 5 | 5 | 5 | 4.85452 |
| strongly agree | strongly agree | strongly agree | strongly agree | strongly agree | strongly agree | 5 | 5 | 5 | 5 | 5 | 5 | 4.85452 |
| strongly agree | strongly agree | strongly agree | strongly agree | strongly agree | strongly agree | 5 | 5 | 5 | 5 | 5 | 5 | 4.85452 |
| strongly agree | strongly agree | strongly agree | strongly agree | strongly agree | strongly agree | 5 | 5 | 5 | 5 | 5 | 5 | 4.85452 |
| strongly agree | strongly agree | strongly agree | strongly agree | strongly agree | strongly agree | 5 | 5 | 5 | 5 | 5 | 5 | 4.85452 |
| strongly agree | strongly agree | strongly agree | strongly agree | strongly agree | strongly agree | 5 | 5 | 5 | 5 | 5 | 5 | 4.85452 |
| strongly agree | strongly agree | strongly agree | strongly agree | strongly agree | strongly agree | 5 | 5 | 5 | 5 | 5 | 5 | 4.85452 |
| strongly agree | strongly agree | strongly agree | strongly agree | strongly agree | strongly agree | 5 | 5 | 5 | 5 | 5 | 5 | 4.85452 |
| strongly agree | strongly agree | strongly agree | strongly agree | strongly agree | strongly agree | 5 | 5 | 5 | 5 | 5 | 5 | 4.85452 |
| strongly agree | strongly agree | strongly agree | strongly agree | strongly agree | strongly agree | 5 | 5 | 5 | 5 | 5 | 5 | 4.85452 |
| strongly agree | strongly agree | strongly agree | strongly agree | strongly agree | strongly agree | 5 | 5 | 5 | 5 | 5 | 5 | 4.85452 |
| strongly agree | strongly agree | strongly agree | strongly agree | strongly agree | strongly agree | 5 | 5 | 5 | 5 | 5 | 5 | 4.85452 |
| strongly agree | strongly agree | strongly agree | strongly agree | strongly agree | strongly agree | 5 | 5 | 5 | 5 | 5 | 5 | 4.85452 |
| strongly agree | strongly agree | strongly agree | strongly agree | strongly agree | strongly agree | 5 | 5 | 5 | 5 | 5 | 5 | 4.85452 |
| strongly agree | strongly agree | strongly agree | strongly agree | strongly agree | strongly agree | 5 | 5 | 5 | 5 | 5 | 5 | 4.85452 |
| strongly agree | strongly agree | strongly agree | strongly agree | strongly agree | strongly agree | 5 | 5 | 5 | 5 | 5 | 5 | 4.85452 |
| strongly agree | strongly agree | strongly agree | strongly agree | strongly agree | strongly agree | 5 | 5 | 5 | 5 | 5 | 5 | 4.85452 |
| strongly agree | strongly agree | strongly agree | strongly agree | strongly agree | strongly agree | 5 | 5 | 5 | 5 | 5 | 5 | 4.85452 |
| strongly agree | strongly agree | strongly agree | strongly agree | strongly agree | strongly agree | 5 | 5 | 5 | 5 | 5 | 5 | 4.85452 |
| strongly agree | strongly agree | strongly agree | strongly agree | strongly agree | strongly agree | 5 | 5 | 5 | 5 | 5 | 5 | 4.85452 |
| strongly agree | strongly agree | strongly agree | strongly agree | strongly agree | strongly agree | 5 | 5 | 5 | 5 | 5 | 5 | 4.85452 |
| strongly agree | strongly agree | strongly agree | strongly agree | strongly agree | strongly agree | 5 | 5 | 5 | 5 | 5 | 5 | 4.85452 |
| strongly agree | strongly agree | strongly agree | strongly agree | strongly agree | strongly agree | 5 | 5 | 5 | 5 | 5 | 5 | 4.85452 |
| strongly agree | strongly agree | strongly agree | strongly agree | strongly agree | strongly agree | 5 | 5 | 5 | 5 | 5 | 5 | 4.85452 |
| strongly agree | strongly agree | strongly agree | strongly agree | strongly agree | strongly agree | 5 | 5 | 5 | 5 | 5 | 5 | 4.85452 |
| strongly agree | strongly agree | strongly agree | strongly agree | strongly agree | strongly agree | 5 | 5 | 5 | 5 | 5 | 5 | 4.85452 |
| strongly agree | strongly agree | strongly agree | strongly agree | strongly agree | strongly agree | 5 | 5 | 5 | 5 | 5 | 5 | 4.85452 |
| strongly agree | strongly agree | strongly agree | strongly agree | strongly agree | strongly agree | 5 | 5 | 5 | 5 | 5 | 5 | 4.85452 |
| strongly agree | strongly agree | strongly agree | strongly agree | strongly agree | strongly agree | 5 | 5 | 5 | 5 | 5 | 5 | 4.85452 |
| strongly agree | strongly agree | strongly agree | strongly agree | strongly agree | strongly agree | 5 | 5 | 5 | 5 | 5 | 5 | 4.85452 |
| strongly agree | strongly agree | strongly agree | strongly agree | strongly agree | strongly agree | 5 | 5 | 5 | 5 | 5 | 5 | 4.85452 |
| strongly agree | strongly agree | strongly agree | strongly agree | strongly agree | strongly agree | 5 | 5 | 5 | 5 | 5 | 5 | 4.85452 |
| strongly agree | strongly agree | strongly agree | strongly agree | strongly agree | strongly agree | 5 | 5 | 5 | 5 | 5 | 5 | 4.85452 |
| strongly agree | strongly agree | strongly agree | strongly agree | strongly agree | strongly agree | 5 | 5 | 5 | 5 | 5 | 5 | 4.85452 |
| strongly agree | strongly agree | strongly agree | strongly agree | strongly agree | strongly agree | 5 | 5 | 5 | 5 | 5 | 5 | 4.85452 |
| strongly agree | strongly agree | strongly agree | strongly agree | strongly agree | strongly agree | 5 | 5 | 5 | 5 | 5 | 5 | 4.85452 |
| strongly agree | strongly agree | strongly agree | strongly agree | strongly agree | strongly agree | 5 | 5 | 5 | 5 | 5 | 5 | 4.85452 |
| strongly agree | strongly agree | strongly agree | strongly agree | strongly agree | strongly agree | 5 | 5 | 5 | 5 | 5 | 5 | 4.85452 |
| strongly agree | strongly agree | strongly agree | strongly agree | strongly agree | strongly agree | 5 | 5 | 5 | 5 | 5 | 5 | 4.85452 |
| strongly agree | strongly agree | strongly agree | strongly agree | strongly agree | strongly agree | 5 | 5 | 5 | 5 | 5 | 5 | 4.85452 |
| strongly agree | strongly agree | strongly agree | strongly agree | strongly agree | strongly agree | 5 | 5 | 5 | 5 | 5 | 5 | 4.85452 |
| strongly agree | strongly agree | strongly agree | strongly agree | strongly agree | strongly agree | 5 | 5 | 5 | 5 | 5 | 5 | 4.85452 |
| strongly agree | strongly agree | strongly agree | strongly agree | strongly agree | strongly agree | 5 | 5 | 5 | 5 | 5 | 5 | 4.85452 |
| strongly agree | strongly agree | strongly agree | strongly agree | strongly agree | strongly agree | 5 | 5 | 5 | 5 | 5 | 5 | 4.85452 |
| strongly agree | strongly agree | strongly agree | strongly agree | strongly agree | strongly agree | 5 | 5 | 5 | 5 | 5 | 5 | 4.85452 |
| strongly agree | strongly agree | strongly agree | strongly agree | strongly agree | strongly agree | 5 | 5 | 5 | 5 | 5 | 5 | 4.85452 |
| strongly agree | strongly agree | strongly agree | strongly agree | strongly agree | strongly agree | 5 | 5 | 5 | 5 | 5 | 5 | 4.85452 |
| strongly agree | strongly agree | strongly agree | strongly agree | strongly agree | strongly agree | 5 | 5 | 5 | 5 | 5 | 5 | 4.85452 |
| strongly agree | strongly agree | strongly agree | strongly agree | strongly agree | strongly agree | 5 | 5 | 5 | 5 | 5 | 5 | 4.85452 |
| strongly agree | strongly agree | strongly agree | strongly agree | strongly agree | strongly agree | 2 | 5 | 5 | 5 | 5 | 5 | 4.85452 |
| strongly agree | strongly agree | strongly agree | strongly agree | strongly agree | strongly agree | 4 | 5 | 5 | 5 | 5 | 5 | 4.85452 |
| strongly agree | strongly agree | strongly agree | strongly agree | strongly agree | strongly agree | 4 | 5 | 5 | 5 | 5 | 5 | 4.85452 |
| strongly agree | strongly agree | strongly agree | strongly agree | strongly agree | strongly agree | 4 | 5 | 5 | 5 | 5 | 5 | 4.85452 |
| strongly agree | strongly agree | strongly agree | strongly agree | strongly agree | strongly agree | 4 | 5 | 5 | 5 | 5 | 5 | 4.85452 |
| strongly agree | strongly agree | strongly agree | strongly agree | strongly agree | strongly agree | 4 | 5 | 5 | 5 | 5 | 5 | 4.85452 |
| strongly agree | strongly agree | strongly agree | strongly agree | strongly agree | strongly agree | 4 | 5 | 5 | 5 | 5 | 5 | 4.85452 |
| strongly agree | strongly agree | strongly agree | strongly agree | strongly agree | strongly agree | 4 | 5 | 5 | 5 | 5 | 5 | 4.85452 |
| strongly agree | strongly agree | strongly agree | strongly agree | strongly agree | strongly agree | 4 | 5 | 5 | 5 | 5 | 5 | 4.85452 |
| strongly agree | strongly agree | strongly agree | strongly agree | strongly agree | strongly agree | 4 | 5 | 5 | 5 | 5 | 5 | 4.85452 |
| strongly agree | strongly agree | strongly agree | strongly agree | strongly agree | strongly agree | 4 | 5 | 5 | 5 | 5 | 5 | 4.85452 |
| strongly agree | strongly agree | strongly agree | strongly agree | strongly agree | strongly agree | 3 | 5 | 5 | 5 | 5 | 5 | 4.85452 |
| strongly agree | strongly agree | strongly agree | strongly agree | strongly agree | strongly agree | 3 | 5 | 5 | 5 | 5 | 5 | 4.85452 |
| strongly agree | strongly agree | strongly agree | strongly agree | strongly agree | strongly agree | 3 | 5 | 5 | 5 | 5 | 5 | 4.85452 |
| strongly agree | strongly agree | strongly agree | strongly agree | strongly agree | strongly agree | 3 | 5 | 5 | 5 | 5 | 5 | 4.85452 |
| strongly agree | strongly agree | strongly agree | strongly agree | strongly agree | strongly agree | 3 | 5 | 5 | 5 | 5 | 5 | 4.85452 |
| strongly agree | strongly agree | strongly agree | strongly agree | strongly agree | strongly agree | 2 | 5 | 5 | 5 | 5 | 5 | 4.85452 |
| strongly agree | strongly agree | strongly agree | strongly agree | strongly agree | strongly agree | 2 | 5 | 5 | 5 | 5 | 5 | 4.85452 |
| strongly agree | strongly agree | strongly agree | strongly agree | strongly agree | strongly agree | 2 | 5 | 5 | 5 | 5 | 5 | 4.85452 |
| strongly agree | strongly agree | strongly agree | strongly agree | strongly agree | strongly agree | 3 | 5 | 5 | 5 | 5 | 5 | 4.85452 |
| strongly agree | strongly agree | strongly agree | strongly agree | strongly agree | strongly agree | 3 | 5 | 5 | 5 | 5 | 5 | 4.85452 |
| strongly agree | strongly agree | strongly agree | strongly agree | strongly agree | strongly agree | 3 | 5 | 5 | 5 | 5 | 5 | 4.85452 |
| strongly agree | strongly agree | strongly agree | strongly agree | strongly agree | strongly agree | 3 | 5 | 5 | 5 | 5 | 5 | 4.85452 |
| strongly agree | strongly agree | strongly agree | strongly agree | strongly agree | strongly agree | 2 | 5 | 5 | 5 | 5 | 5 | 4.85452 |
| strongly agree | strongly agree | strongly agree | strongly agree | strongly agree | strongly agree | 3 | 5 | 5 | 5 | 5 | 5 | 4.85452 |
| strongly agree | strongly agree | strongly agree | strongly agree | strongly agree | strongly agree | 2 | 5 | 5 | 5 | 5 | 5 | 4.85452 |
| strongly agree | strongly agree | strongly agree | strongly agree | strongly agree | strongly agree | 3 | 5 | 5 | 5 | 5 | 5 | 4.85452 |
| strongly agree | strongly agree | strongly agree | strongly agree | strongly agree | strongly agree | 3 | 5 | 5 | 5 | 5 | 5 | 4.85452 |
| strongly agree | strongly agree | strongly agree | strongly agree | strongly agree | strongly agree | 2 | 5 | 5 | 5 | 5 | 5 | 4.85452 |
| strongly agree | strongly agree | strongly agree | strongly agree | strongly agree | strongly agree | 2 | 5 | 5 | 5 | 5 | 5 | 4.85452 |
| strongly agree | strongly agree | strongly agree | strongly agree | strongly agree | strongly agree | 3 | 5 | 5 | 5 | 5 | 5 | 4.85452 |
| strongly agree | strongly agree | strongly agree | strongly agree | disagree | disagree | 2 | 2 | 2 | 4 | 5 | 4 | 3.67802 |
| strongly agree | strongly agree | strongly agree | strongly agree | strongly disagree | strongly disagree | 1 | 1 | 1 | 3 | 5 | 4 | 3.28585 |
| strongly agree | strongly agree | strongly agree | strongly agree | disagree | disagree | 2 | 2 | 2 | 4 | 5 | 4 | 3.67802 |
| strongly agree | strongly agree | strongly agree | strongly agree | disagree | disagree | 2 | 2 | 2 | 4 | 5 | 4 | 3.67802 |
| strongly agree | strongly agree | strongly agree | strongly agree | disagree | disagree | 2 | 2 | 2 | 4 | 5 | 4 | 3.67802 |
| strongly agree | strongly agree | strongly agree | strongly agree | disagree | disagree | 2 | 2 | 2 | 4 | 5 | 4 | 3.67802 |
| strongly agree | strongly agree | strongly agree | strongly agree | strongly disagree | strongly disagree | 2 | 1 | 1 | 3 | 5 | 4 | 3.28585 |
| strongly agree | strongly agree | strongly agree | strongly agree | disagree | disagree | 2 | 2 | 2 | 4 | 5 | 4 | 3.67802 |
| strongly agree | strongly agree | strongly agree | strongly agree | strongly disagree | strongly disagree | 1 | 1 | 1 | 3 | 5 | 3 | 3.28585 |
| strongly agree | strongly agree | strongly agree | strongly agree | Neutral | Neutral | 3 | 3 | 3 | 4 | 5 | 3 | 4.07019 |
| strongly agree | strongly agree | strongly agree | strongly agree | disagree | disagree | 2 | 2 | 2 | 4 | 5 | 3 | 3.67802 |
| strongly agree | strongly agree | strongly agree | strongly agree | disagree | disagree | 2 | 2 | 2 | 4 | 5 | 3 | 3.67802 |
| strongly agree | strongly agree | strongly agree | strongly agree | disagree | disagree | 2 | 2 | 2 | 4 | 5 | 3 | 3.67802 |
| disagree | disagree | disagree | disagree | disagree | disagree | 2 | 2 | 2 | 2 | 2 | 5 | 2.26444 |
| disagree | disagree | disagree | disagree | disagree | disagree | 2 | 2 | 2 | 2 | 2 | 2 | 2.26444 |
| disagree | disagree | disagree | disagree | disagree | disagree | 2 | 2 | 2 | 2 | 2 | 2 | 2.26444 |
| disagree | disagree | disagree | disagree | disagree | disagree | 2 | 2 | 2 | 2 | 2 | 2 | 2.26444 |
| strongly disagree | strongly disagree | strongly disagree | strongly disagree | strongly disagree | strongly disagree | 2 | 1 | 1 | 1 | 1 | 2 | 1.40108 |
| disagree | disagree | disagree | disagree | disagree | disagree | 2 | 2 | 2 | 2 | 2 | 2 | 2.26444 |
| disagree | disagree | disagree | disagree | disagree | disagree | 2 | 2 | 2 | 2 | 2 | 1 | 2.26444 |
| strongly disagree | strongly disagree | strongly disagree | strongly disagree | strongly disagree | strongly disagree | 2 | 1 | 1 | 1 | 1 | 1 | 1.40108 |
| strongly disagree | strongly disagree | strongly disagree | strongly disagree | strongly disagree | strongly disagree | 2 | 1 | 1 | 1 | 1 | 1 | 1.40108 |
| Neutral | Neutral | Neutral | Neutral | Neutral | Neutral | 2 | 3 | 3 | 3 | 3 | 1 | 3.1278 |
| disagree | disagree | disagree | disagree | disagree | disagree | 3 | 2 | 2 | 2 | 2 | 1 | 2.26444 |
| strongly disagree | strongly disagree | strongly disagree | strongly disagree | strongly disagree | strongly disagree | 2 | 1 | 1 | 1 | 1 | 3 | 1.40108 |
| disagree | disagree | disagree | disagree | disagree | disagree | 2 | 2 | 2 | 2 | 2 | 3 | 2.26444 |
| strongly disagree | strongly disagree | strongly disagree | strongly disagree | strongly disagree | strongly disagree | 2 | 1 | 1 | 1 | 1 | 3 | 1.40108 |
| disagree | disagree | disagree | disagree | disagree | disagree | 2 | 2 | 2 | 2 | 2 | 3 | 2.26444 |
| strongly disagree | strongly disagree | strongly disagree | strongly disagree | strongly disagree | strongly disagree | 2 | 1 | 1 | 1 | 1 | 3 | 1.40108 |
| disagree | disagree | disagree | disagree | disagree | disagree | 3 | 2 | 2 | 2 | 2 | 1 | 2.26444 |
| disagree | disagree | disagree | disagree | disagree | disagree | 2 | 2 | 2 | 2 | 2 | 1 | 2.26444 |
| disagree | disagree | disagree | disagree | disagree | disagree | 2 | 2 | 2 | 2 | 2 | 1 | 2.26444 |
| disagree | disagree | disagree | disagree | disagree | disagree | 2 | 2 | 2 | 2 | 2 | 1 | 2.26444 |
| disagree | disagree | disagree | disagree | disagree | disagree | 2 | 2 | 2 | 2 | 2 | 1 | 2.26444 |
| Neutral | Neutral | Neutral | Neutral | Neutral | Neutral | 2 | 3 | 3 | 3 | 3 | 1 | 3.1278 |
| disagree | disagree | disagree | disagree | disagree | disagree | 2 | 2 | 2 | 2 | 2 | 3 | 2.26444 |
| Neutral | Neutral | Neutral | Neutral | Neutral | Neutral | 3 | 3 | 3 | 3 | 3 | 3 | 3.1278 |
| disagree | disagree | disagree | disagree | disagree | disagree | 2 | 2 | 2 | 2 | 2 | 3 | 2.26444 |
| Neutral | Neutral | Neutral | Neutral | Neutral | Neutral | 2 | 3 | 3 | 3 | 3 | 3 | 3.1278 |
| disagree | disagree | disagree | disagree | disagree | disagree | 2 | 2 | 2 | 2 | 2 | 3 | 2.26444 |
| disagree | disagree | disagree | disagree | disagree | disagree | 2 | 2 | 2 | 2 | 2 | 3 | 2.26444 |
| disagree | disagree | disagree | disagree | disagree | disagree | 2 | 2 | 2 | 2 | 2 | 3 | 2.26444 |
| disagree | disagree | disagree | disagree | disagree | disagree | 2 | 2 | 2 | 2 | 2 | 3 | 2.26444 |
| disagree | disagree | disagree | disagree | disagree | disagree | 2 | 2 | 2 | 2 | 2 | 3 | 2.26444 |
| Neutral | Neutral | Neutral | Neutral | Neutral | Neutral | 2 | 3 | 3 | 3 | 3 | 3 | 3.1278 |
| disagree | disagree | disagree | disagree | disagree | disagree | 2 | 2 | 2 | 2 | 2 | 3 | 2.26444 |
| Neutral | Neutral | Neutral | Neutral | Neutral | Neutral | 2 | 3 | 3 | 3 | 3 | 3 | 3.1278 |
| disagree | disagree | disagree | disagree | disagree | disagree | 2 | 2 | 2 | 2 | 2 | 3 | 2.26444 |
| Neutral | Neutral | Neutral | Neutral | Neutral | Neutral | 2 | 3 | 3 | 3 | 3 | 3 | 3.1278 |
| disagree | disagree | disagree | disagree | disagree | disagree | 2 | 2 | 2 | 2 | 2 | 3 | 2.26444 |
| Neutral | Neutral | Neutral | Neutral | Neutral | Neutral | 2 | 3 | 3 | 3 | 3 | 3 | 3.1278 |
| Neutral | Neutral | Neutral | Neutral | Neutral | Neutral | 2 | 3 | 3 | 3 | 3 | 3 | 3.1278 |
| disagree | disagree | disagree | disagree | disagree | disagree | 2 | 2 | 2 | 2 | 2 | 3 | 2.26444 |
| Neutral | Neutral | Neutral | Neutral | Neutral | Neutral | 3 | 3 | 3 | 3 | 3 | 3 | 3.1278 |
| disagree | disagree | disagree | disagree | disagree | disagree | 2 | 2 | 2 | 2 | 2 | 3 | 2.26444 |
| Neutral | Neutral | Neutral | Neutral | Neutral | Neutral | 2 | 3 | 3 | 3 | 3 | 3 | 3.1278 |
| disagree | disagree | disagree | disagree | disagree | disagree | 2 | 2 | 2 | 2 | 2 | 3 | 2.26444 |
| disagree | disagree | disagree | disagree | disagree | disagree | 2 | 2 | 2 | 2 | 2 | 3 | 2.26444 |
| disagree | disagree | disagree | disagree | disagree | disagree | 2 | 2 | 2 | 2 | 2 | 2 | 2.26444 |
| disagree | disagree | disagree | disagree | disagree | disagree | 3 | 2 | 2 | 2 | 2 | 2 | 2.26444 |
| disagree | disagree | disagree | disagree | disagree | disagree | 2 | 2 | 2 | 2 | 2 | 2 | 2.26444 |
| disagree | disagree | disagree | disagree | disagree | disagree | 3 | 2 | 2 | 2 | 2 | 2 | 2.26444 |
| disagree | disagree | disagree | disagree | disagree | disagree | 3 | 2 | 2 | 2 | 2 | 2 | 2.26444 |
| disagree | disagree | disagree | disagree | disagree | disagree | 3 | 2 | 2 | 2 | 2 | 2 | 2.26444 |
| disagree | disagree | disagree | disagree | disagree | disagree | 3 | 2 | 2 | 2 | 2 | 2 | 2.26444 |
| disagree | disagree | disagree | disagree | disagree | disagree | 2 | 2 | 2 | 2 | 2 | 2 | 2.26444 |
| disagree | disagree | disagree | disagree | disagree | disagree | 3 | 2 | 2 | 2 | 2 | 2 | 2.26444 |
| disagree | disagree | disagree | disagree | disagree | disagree | 2 | 2 | 2 | 2 | 2 | 2 | 2.26444 |
| Neutral | Neutral | Neutral | Neutral | Neutral | Neutral | 3 | 3 | 3 | 3 | 3 | 2 | 3.1278 |
| disagree | disagree | disagree | disagree | disagree | disagree | 2 | 2 | 2 | 2 | 2 | 2 | 2.26444 |
| disagree | disagree | disagree | disagree | disagree | disagree | 2 | 2 | 2 | 2 | 2 | 2 | 2.26444 |
| disagree | disagree | disagree | disagree | disagree | disagree | 3 | 2 | 2 | 2 | 2 | 2 | 2.26444 |
| disagree | disagree | disagree | disagree | disagree | disagree | 3 | 2 | 2 | 2 | 2 | 2 | 2.26444 |
| disagree | disagree | disagree | disagree | disagree | disagree | 3 | 2 | 2 | 2 | 2 | 2 | 2.26444 |
| Neutral | Neutral | Neutral | Neutral | Neutral | Neutral | 3 | 3 | 3 | 3 | 3 | 2 | 3.1278 |
| disagree | disagree | disagree | disagree | disagree | disagree | 2 | 2 | 2 | 2 | 2 | 2 | 2.26444 |
| Neutral | Neutral | Neutral | Neutral | Neutral | Neutral | 2 | 3 | 3 | 3 | 3 | 2 | 3.1278 |

| ZPR_1 | ZRE_1 | SRE_1 | COO_1 | COV_1 |
| --- | --- | --- | --- | --- |
| -1.24766 | 0.34612 | 0.35465 | 0.00157 | 1.06098 |
| -1.24766 | 0.34612 | 0.35465 | 0.00157 | 1.06098 |
| -1.24766 | 0.34612 | 0.35465 | 0.00157 | 1.06098 |
| -1.4574 | 0.74865 | 0.76682 | 0.00722 | 1.05436 |
| -1.76659 | 1.34202 | 1.35889 | 0.01167 | 1.01484 |
| -1.4574 | 0.74865 | 0.76682 | 0.00722 | 1.05436 |
| -1.4574 | 0.74865 | 0.76682 | 0.00722 | 1.05436 |
| -1.4574 | 0.74865 | 0.76682 | 0.00722 | 1.05436 |
| -1.4574 | 0.74865 | 0.76682 | 0.00722 | 1.05436 |
| -1.4574 | 0.74865 | 0.76682 | 0.00722 | 1.05436 |
| -1.6252 | 1.07067 | 1.10395 | 0.01923 | 1.06032 |
| -1.6252 | 1.07067 | 1.10395 | 0.01923 | 1.06032 |
| -1.6252 | 1.07067 | 1.10395 | 0.01923 | 1.06032 |
| -1.6252 | 1.07067 | 1.10395 | 0.01923 | 1.06032 |
| -1.6252 | 1.07067 | 1.10395 | 0.01923 | 1.06032 |
| -1.4605 | 0.7546 | 0.77153 | 0.00675 | 1.05049 |
| -1.4605 | 0.7546 | 0.77153 | 0.00675 | 1.05049 |
| -1.4605 | 0.7546 | 0.77153 | 0.00675 | 1.05049 |
| -1.4605 | -1.02835 | -1.05141 | 0.01254 | 1.04403 |
| -1.29581 | -1.34441 | -1.37168 | 0.01927 | 1.02992 |
| -1.29581 | -1.34441 | -1.37168 | 0.01927 | 1.02992 |
| -0.79242 | -2.31048 | -2.32269 | 0.0143 | 0.95799 |
| -0.79242 | -2.31048 | -2.32269 | 0.0143 | 0.95799 |
| -0.79242 | -2.31048 | -2.32269 | 0.0143 | 0.95799 |
| -0.79242 | -2.31048 | -2.32269 | 0.0143 | 0.95799 |
| -0.62773 | -2.62654 | -2.63361 | 0.00934 | 0.93518 |
| -0.62773 | -2.62654 | -2.63361 | 0.00934 | 0.93518 |
| -0.00934 | -3.81329 | -3.87093 | 0.1141 | 0.86705 |
| -0.00934 | -3.81329 | -3.87093 | 0.1141 | 0.86705 |
| -0.00934 | -3.81329 | -3.87093 | 0.1141 | 0.86705 |
| 0.15535 | -0.56346 | -0.56935 | 0.0017 | 1.02938 |
| 0.15535 | -0.56346 | -0.56935 | 0.0017 | 1.02938 |
| 0.15535 | -0.56346 | -0.56935 | 0.0017 | 1.02938 |
| 0.15535 | -0.56346 | -0.56935 | 0.0017 | 1.02938 |
| 0.49095 | 0.57545 | 0.57837 | 0.00085 | 1.01832 |
| 0.49095 | 0.57545 | 0.57837 | 0.00085 | 1.01832 |
| 0.65564 | 0.25938 | 0.25995 | 0.00007 | 1.01573 |
| 0.65564 | 0.25938 | 0.25995 | 0.00007 | 1.01573 |
| 0.65564 | 0.25938 | 0.25995 | 0.00007 | 1.01573 |
| 0.48784 | 0.5814 | 0.58378 | 0.0007 | 1.01626 |
| 0.48784 | 0.5814 | 0.58378 | 0.0007 | 1.01626 |
| 0.48784 | 0.5814 | 0.58378 | 0.0007 | 1.01626 |
| 0.65564 | 0.25938 | 0.25995 | 0.00007 | 1.01573 |
| 0.65564 | 0.25938 | 0.25995 | 0.00007 | 1.01573 |
| 0.65564 | 0.25938 | 0.25995 | 0.00007 | 1.01573 |
| 0.65564 | 0.25938 | 0.25995 | 0.00007 | 1.01573 |
| 0.65564 | 0.25938 | 0.25995 | 0.00007 | 1.01573 |
| 0.65564 | 0.25938 | 0.25995 | 0.00007 | 1.01573 |
| 0.65564 | 0.25938 | 0.25995 | 0.00007 | 1.01573 |
| 0.65564 | 0.25938 | 0.25995 | 0.00007 | 1.01573 |
| 0.65564 | 0.25938 | 0.25995 | 0.00007 | 1.01573 |
| 0.65564 | 0.25938 | 0.25995 | 0.00007 | 1.01573 |
| 0.65564 | 0.25938 | 0.25995 | 0.00007 | 1.01573 |
| 0.65564 | 0.25938 | 0.25995 | 0.00007 | 1.01573 |
| 0.65564 | 0.25938 | 0.25995 | 0.00007 | 1.01573 |
| 0.65564 | 0.25938 | 0.25995 | 0.00007 | 1.01573 |
| 0.65564 | 0.25938 | 0.25995 | 0.00007 | 1.01573 |
| 0.65564 | 0.25938 | 0.25995 | 0.00007 | 1.01573 |
| 0.65564 | 0.25938 | 0.25995 | 0.00007 | 1.01573 |
| 0.65564 | 0.25938 | 0.25995 | 0.00007 | 1.01573 |
| 0.65564 | 0.25938 | 0.25995 | 0.00007 | 1.01573 |
| 0.65564 | 0.25938 | 0.25995 | 0.00007 | 1.01573 |
| 0.65564 | 0.25938 | 0.25995 | 0.00007 | 1.01573 |
| 0.65564 | 0.25938 | 0.25995 | 0.00007 | 1.01573 |
| 0.65564 | 0.25938 | 0.25995 | 0.00007 | 1.01573 |
| 0.65564 | 0.25938 | 0.25995 | 0.00007 | 1.01573 |
| 0.65564 | 0.25938 | 0.25995 | 0.00007 | 1.01573 |
| 0.65564 | 0.25938 | 0.25995 | 0.00007 | 1.01573 |
| 0.65564 | 0.25938 | 0.25995 | 0.00007 | 1.01573 |
| 0.65564 | 0.25938 | 0.25995 | 0.00007 | 1.01573 |
| 0.65564 | 0.25938 | 0.25995 | 0.00007 | 1.01573 |
| 0.65564 | 0.25938 | 0.25995 | 0.00007 | 1.01573 |
| 0.65564 | 0.25938 | 0.25995 | 0.00007 | 1.01573 |
| 0.65564 | 0.25938 | 0.25995 | 0.00007 | 1.01573 |
| 0.65564 | 0.25938 | 0.25995 | 0.00007 | 1.01573 |
| 0.65564 | 0.25938 | 0.25995 | 0.00007 | 1.01573 |
| 0.65564 | 0.25938 | 0.25995 | 0.00007 | 1.01573 |
| 0.65564 | 0.25938 | 0.25995 | 0.00007 | 1.01573 |
| 0.65564 | 0.25938 | 0.25995 | 0.00007 | 1.01573 |
| 0.65564 | 0.25938 | 0.25995 | 0.00007 | 1.01573 |
| 0.65564 | 0.25938 | 0.25995 | 0.00007 | 1.01573 |
| 0.65564 | 0.25938 | 0.25995 | 0.00007 | 1.01573 |
| 0.65564 | 0.25938 | 0.25995 | 0.00007 | 1.01573 |
| 0.65564 | 0.25938 | 0.25995 | 0.00007 | 1.01573 |
| 0.65564 | 0.25938 | 0.25995 | 0.00007 | 1.01573 |
| 0.65564 | 0.25938 | 0.25995 | 0.00007 | 1.01573 |
| 0.65564 | -1.52357 | -1.52689 | 0.00254 | 0.9883 |
| 0.65564 | -1.52357 | -1.52689 | 0.00254 | 0.9883 |
| 0.65564 | -1.52357 | -1.52689 | 0.00254 | 0.9883 |
| 0.65564 | -1.52357 | -1.52689 | 0.00254 | 0.9883 |
| 0.65564 | -1.52357 | -1.52689 | 0.00254 | 0.9883 |
| 0.65564 | -1.52357 | -1.52689 | 0.00254 | 0.9883 |
| 0.65564 | -1.52357 | -1.52689 | 0.00254 | 0.9883 |
| 0.65564 | -1.52357 | -1.52689 | 0.00254 | 0.9883 |
| 0.65564 | -1.52357 | -1.52689 | 0.00254 | 0.9883 |
| 0.65564 | -1.52357 | -1.52689 | 0.00254 | 0.9883 |
| 0.65564 | -1.52357 | -1.52689 | 0.00254 | 0.9883 |
| 0.65564 | 0.25938 | 0.25995 | 0.00007 | 1.01573 |
| 0.65564 | 0.25938 | 0.25995 | 0.00007 | 1.01573 |
| 0.65564 | 0.25938 | 0.25995 | 0.00007 | 1.01573 |
| 0.65564 | 0.25938 | 0.25995 | 0.00007 | 1.01573 |
| 0.65564 | 0.25938 | 0.25995 | 0.00007 | 1.01573 |
| 0.65564 | 0.25938 | 0.25995 | 0.00007 | 1.01573 |
| 0.65564 | 0.25938 | 0.25995 | 0.00007 | 1.01573 |
| 0.65564 | 0.25938 | 0.25995 | 0.00007 | 1.01573 |
| 0.65564 | 0.25938 | 0.25995 | 0.00007 | 1.01573 |
| 0.65564 | 0.25938 | 0.25995 | 0.00007 | 1.01573 |
| 0.65564 | 0.25938 | 0.25995 | 0.00007 | 1.01573 |
| 0.65564 | 0.25938 | 0.25995 | 0.00007 | 1.01573 |
| 0.65564 | 0.25938 | 0.25995 | 0.00007 | 1.01573 |
| 0.65564 | 0.25938 | 0.25995 | 0.00007 | 1.01573 |
| 0.65564 | 0.25938 | 0.25995 | 0.00007 | 1.01573 |
| 0.65564 | 0.25938 | 0.25995 | 0.00007 | 1.01573 |
| 0.65564 | 0.25938 | 0.25995 | 0.00007 | 1.01573 |
| 0.65564 | 0.25938 | 0.25995 | 0.00007 | 1.01573 |
| 0.65564 | 0.25938 | 0.25995 | 0.00007 | 1.01573 |
| 0.65564 | 0.25938 | 0.25995 | 0.00007 | 1.01573 |
| 0.65564 | 0.25938 | 0.25995 | 0.00007 | 1.01573 |
| 0.65564 | 0.25938 | 0.25995 | 0.00007 | 1.01573 |
| 0.65564 | 0.25938 | 0.25995 | 0.00007 | 1.01573 |
| 0.65564 | 0.25938 | 0.25995 | 0.00007 | 1.01573 |
| 0.65564 | 0.25938 | 0.25995 | 0.00007 | 1.01573 |
| 0.65564 | 0.25938 | 0.25995 | 0.00007 | 1.01573 |
| 0.65564 | 0.25938 | 0.25995 | 0.00007 | 1.01573 |
| 0.65564 | 0.25938 | 0.25995 | 0.00007 | 1.01573 |
| 0.65564 | 0.25938 | 0.25995 | 0.00007 | 1.01573 |
| 0.65564 | 0.25938 | 0.25995 | 0.00007 | 1.01573 |
| 0.65564 | 0.25938 | 0.25995 | 0.00007 | 1.01573 |
| 0.65564 | 0.25938 | 0.25995 | 0.00007 | 1.01573 |
| 0.65564 | 0.25938 | 0.25995 | 0.00007 | 1.01573 |
| 0.65564 | 0.25938 | 0.25995 | 0.00007 | 1.01573 |
| 0.65564 | 0.25938 | 0.25995 | 0.00007 | 1.01573 |
| 0.65564 | 0.25938 | 0.25995 | 0.00007 | 1.01573 |
| 0.65564 | -1.52357 | -1.52689 | 0.00254 | 0.9883 |
| 0.65564 | -1.52357 | -1.52689 | 0.00254 | 0.9883 |
| 0.65564 | -1.52357 | -1.52689 | 0.00254 | 0.9883 |
| 0.65564 | -1.52357 | -1.52689 | 0.00254 | 0.9883 |
| 0.65564 | -1.52357 | -1.52689 | 0.00254 | 0.9883 |
| 0.65564 | -1.52357 | -1.52689 | 0.00254 | 0.9883 |
| 0.65564 | -1.52357 | -1.52689 | 0.00254 | 0.9883 |
| 0.65564 | -1.52357 | -1.52689 | 0.00254 | 0.9883 |
| 0.65564 | -1.52357 | -1.52689 | 0.00254 | 0.9883 |
| 0.65564 | 0.25938 | 0.25995 | 0.00007 | 1.01573 |
| 0.65564 | 0.25938 | 0.25995 | 0.00007 | 1.01573 |
| 0.65564 | 0.25938 | 0.25995 | 0.00007 | 1.01573 |
| 0.65564 | 0.25938 | 0.25995 | 0.00007 | 1.01573 |
| 0.65564 | 0.25938 | 0.25995 | 0.00007 | 1.01573 |
| 0.65564 | 0.25938 | 0.25995 | 0.00007 | 1.01573 |
| 0.65564 | 0.25938 | 0.25995 | 0.00007 | 1.01573 |
| 0.65564 | 0.25938 | 0.25995 | 0.00007 | 1.01573 |
| 0.65564 | 0.25938 | 0.25995 | 0.00007 | 1.01573 |
| 0.65564 | 0.25938 | 0.25995 | 0.00007 | 1.01573 |
| 0.65564 | 0.25938 | 0.25995 | 0.00007 | 1.01573 |
| 0.65564 | 0.25938 | 0.25995 | 0.00007 | 1.01573 |
| 0.65564 | 0.25938 | 0.25995 | 0.00007 | 1.01573 |
| 0.65564 | 0.25938 | 0.25995 | 0.00007 | 1.01573 |
| 0.65564 | 0.25938 | 0.25995 | 0.00007 | 1.01573 |
| 0.65564 | 0.25938 | 0.25995 | 0.00007 | 1.01573 |
| 0.65564 | 0.25938 | 0.25995 | 0.00007 | 1.01573 |
| 0.65564 | 0.25938 | 0.25995 | 0.00007 | 1.01573 |
| 0.65564 | 0.25938 | 0.25995 | 0.00007 | 1.01573 |
| 0.65564 | 0.25938 | 0.25995 | 0.00007 | 1.01573 |
| 0.65564 | 0.25938 | 0.25995 | 0.00007 | 1.01573 |
| 0.65564 | 0.25938 | 0.25995 | 0.00007 | 1.01573 |
| 0.65564 | 0.25938 | 0.25995 | 0.00007 | 1.01573 |
| 0.65564 | 0.25938 | 0.25995 | 0.00007 | 1.01573 |
| 0.65564 | 0.25938 | 0.25995 | 0.00007 | 1.01573 |
| 0.65564 | 0.25938 | 0.25995 | 0.00007 | 1.01573 |
| 0.65564 | 0.25938 | 0.25995 | 0.00007 | 1.01573 |
| 0.65564 | 0.25938 | 0.25995 | 0.00007 | 1.01573 |
| 0.65564 | 0.25938 | 0.25995 | 0.00007 | 1.01573 |
| 0.65564 | 0.25938 | 0.25995 | 0.00007 | 1.01573 |
| 0.65564 | 0.25938 | 0.25995 | 0.00007 | 1.01573 |
| 0.65564 | 0.25938 | 0.25995 | 0.00007 | 1.01573 |
| 0.65564 | 0.25938 | 0.25995 | 0.00007 | 1.01573 |
| 0.65564 | 0.25938 | 0.25995 | 0.00007 | 1.01573 |
| 0.65564 | 0.25938 | 0.25995 | 0.00007 | 1.01573 |
| 0.65564 | 0.25938 | 0.25995 | 0.00007 | 1.01573 |
| 0.65564 | 0.25938 | 0.25995 | 0.00007 | 1.01573 |
| 0.65564 | 0.25938 | 0.25995 | 0.00007 | 1.01573 |
| 0.65564 | 0.25938 | 0.25995 | 0.00007 | 1.01573 |
| 0.65564 | 0.25938 | 0.25995 | 0.00007 | 1.01573 |
| 0.65564 | 0.25938 | 0.25995 | 0.00007 | 1.01573 |
| 0.65564 | 0.25938 | 0.25995 | 0.00007 | 1.01573 |
| 0.65564 | 0.25938 | 0.25995 | 0.00007 | 1.01573 |
| 0.65564 | 0.25938 | 0.25995 | 0.00007 | 1.01573 |
| 0.65564 | 0.25938 | 0.25995 | 0.00007 | 1.01573 |
| 0.65564 | 0.25938 | 0.25995 | 0.00007 | 1.01573 |
| 0.65564 | 0.25938 | 0.25995 | 0.00007 | 1.01573 |
| 0.65564 | 0.25938 | 0.25995 | 0.00007 | 1.01573 |
| 0.65564 | 0.25938 | 0.25995 | 0.00007 | 1.01573 |
| 0.65564 | 0.25938 | 0.25995 | 0.00007 | 1.01573 |
| 0.65564 | 0.25938 | 0.25995 | 0.00007 | 1.01573 |
| 0.65564 | 0.25938 | 0.25995 | 0.00007 | 1.01573 |
| 0.65564 | 0.25938 | 0.25995 | 0.00007 | 1.01573 |
| 0.65564 | 0.25938 | 0.25995 | 0.00007 | 1.01573 |
| 0.65564 | 0.25938 | 0.25995 | 0.00007 | 1.01573 |
| 0.65564 | 0.25938 | 0.25995 | 0.00007 | 1.01573 |
| 0.65564 | 0.25938 | 0.25995 | 0.00007 | 1.01573 |
| 0.65564 | 0.25938 | 0.25995 | 0.00007 | 1.01573 |
| 0.65564 | 0.25938 | 0.25995 | 0.00007 | 1.01573 |
| 0.65564 | 0.25938 | 0.25995 | 0.00007 | 1.01573 |
| 0.65564 | 0.25938 | 0.25995 | 0.00007 | 1.01573 |
| 0.65564 | 0.25938 | 0.25995 | 0.00007 | 1.01573 |
| 0.65564 | 0.25938 | 0.25995 | 0.00007 | 1.01573 |
| 0.65564 | 0.25938 | 0.25995 | 0.00007 | 1.01573 |
| 0.65564 | 0.25938 | 0.25995 | 0.00007 | 1.01573 |
| 0.65564 | 0.25938 | 0.25995 | 0.00007 | 1.01573 |
| 0.65564 | 0.25938 | 0.25995 | 0.00007 | 1.01573 |
| 0.65564 | 0.25938 | 0.25995 | 0.00007 | 1.01573 |
| 0.65564 | 0.25938 | 0.25995 | 0.00007 | 1.01573 |
| 0.65564 | 0.25938 | 0.25995 | 0.00007 | 1.01573 |
| 0.65564 | 0.25938 | 0.25995 | 0.00007 | 1.01573 |
| 0.65564 | 0.25938 | 0.25995 | 0.00007 | 1.01573 |
| 0.65564 | 0.25938 | 0.25995 | 0.00007 | 1.01573 |
| 0.65564 | 0.25938 | 0.25995 | 0.00007 | 1.01573 |
| 0.65564 | 0.25938 | 0.25995 | 0.00007 | 1.01573 |
| 0.65564 | 0.25938 | 0.25995 | 0.00007 | 1.01573 |
| 0.65564 | 0.25938 | 0.25995 | 0.00007 | 1.01573 |
| 0.65564 | 0.25938 | 0.25995 | 0.00007 | 1.01573 |
| 0.65564 | 0.25938 | 0.25995 | 0.00007 | 1.01573 |
| 0.65564 | 0.25938 | 0.25995 | 0.00007 | 1.01573 |
| 0.65564 | 0.25938 | 0.25995 | 0.00007 | 1.01573 |
| 0.65564 | 0.25938 | 0.25995 | 0.00007 | 1.01573 |
| 0.65564 | 0.25938 | 0.25995 | 0.00007 | 1.01573 |
| 0.65564 | 0.25938 | 0.25995 | 0.00007 | 1.01573 |
| 0.65564 | 0.25938 | 0.25995 | 0.00007 | 1.01573 |
| 0.65564 | 0.25938 | 0.25995 | 0.00007 | 1.01573 |
| 0.65564 | 0.25938 | 0.25995 | 0.00007 | 1.01573 |
| 0.65564 | 0.25938 | 0.25995 | 0.00007 | 1.01573 |
| 0.65564 | 0.25938 | 0.25995 | 0.00007 | 1.01573 |
| 0.65564 | 0.25938 | 0.25995 | 0.00007 | 1.01573 |
| 0.65564 | 0.25938 | 0.25995 | 0.00007 | 1.01573 |
| 0.65564 | 0.25938 | 0.25995 | 0.00007 | 1.01573 |
| 0.65564 | 0.25938 | 0.25995 | 0.00007 | 1.01573 |
| 0.65564 | 0.25938 | 0.25995 | 0.00007 | 1.01573 |
| 0.65564 | 0.25938 | 0.25995 | 0.00007 | 1.01573 |
| 0.65564 | 0.25938 | 0.25995 | 0.00007 | 1.01573 |
| 0.65564 | 0.25938 | 0.25995 | 0.00007 | 1.01573 |
| 0.65564 | 0.25938 | 0.25995 | 0.00007 | 1.01573 |
| 0.65564 | 0.25938 | 0.25995 | 0.00007 | 1.01573 |
| 0.65564 | 0.25938 | 0.25995 | 0.00007 | 1.01573 |
| 0.65564 | 0.25938 | 0.25995 | 0.00007 | 1.01573 |
| 0.65564 | 0.25938 | 0.25995 | 0.00007 | 1.01573 |
| 0.65564 | 0.25938 | 0.25995 | 0.00007 | 1.01573 |
| 0.65564 | 0.25938 | 0.25995 | 0.00007 | 1.01573 |
| 0.65564 | 0.25938 | 0.25995 | 0.00007 | 1.01573 |
| 0.65564 | 0.25938 | 0.25995 | 0.00007 | 1.01573 |
| 0.65564 | 0.25938 | 0.25995 | 0.00007 | 1.01573 |
| 0.65564 | 0.25938 | 0.25995 | 0.00007 | 1.01573 |
| 0.65564 | 0.25938 | 0.25995 | 0.00007 | 1.01573 |
| 0.65564 | 0.25938 | 0.25995 | 0.00007 | 1.01573 |
| 0.65564 | 0.25938 | 0.25995 | 0.00007 | 1.01573 |
| 0.65564 | 0.25938 | 0.25995 | 0.00007 | 1.01573 |
| 0.65564 | 0.25938 | 0.25995 | 0.00007 | 1.01573 |
| 0.65564 | 0.25938 | 0.25995 | 0.00007 | 1.01573 |
| 0.65564 | 0.25938 | 0.25995 | 0.00007 | 1.01573 |
| 0.65564 | 0.25938 | 0.25995 | 0.00007 | 1.01573 |
| 0.65564 | 0.25938 | 0.25995 | 0.00007 | 1.01573 |
| -0.43739 | 0.57407 | 0.59074 | 0.00514 | 1.06725 |
| -0.80173 | 1.27329 | 1.34135 | 0.04937 | 1.09909 |
| -0.43739 | 0.57407 | 0.59074 | 0.00514 | 1.06725 |
| -0.43739 | 0.57407 | 0.59074 | 0.00514 | 1.06725 |
| -0.43739 | 0.57407 | 0.59074 | 0.00514 | 1.06725 |
| -0.43739 | 0.57407 | 0.59074 | 0.00514 | 1.06725 |
| -0.80173 | 1.27329 | 1.34135 | 0.04937 | 1.09909 |
| -0.43739 | 0.57407 | 0.59074 | 0.00514 | 1.06725 |
| -0.80173 | -0.50966 | -0.53691 | 0.00791 | 1.11934 |
| -0.07304 | -1.90809 | -1.93282 | 0.02437 | 0.99259 |
| -0.43739 | -1.20888 | -1.24397 | 0.02278 | 1.05191 |
| -0.43739 | -1.20888 | -1.24397 | 0.02278 | 1.05191 |
| -0.43739 | -1.20888 | -1.24397 | 0.02278 | 1.05191 |
| -1.75067 | 4.87737 | 4.91575 | 0.09544 | 0.75992 |
| -1.75067 | -0.47149 | -0.47519 | 0.00089 | 1.02534 |
| -1.75067 | -0.47149 | -0.47519 | 0.00089 | 1.02534 |
| -1.75067 | -0.47149 | -0.47519 | 0.00089 | 1.02534 |
| -2.55277 | 1.06784 | 1.0832 | 0.0085 | 1.02681 |
| -1.75067 | -0.47149 | -0.47519 | 0.00089 | 1.02534 |
| -1.75067 | -2.25444 | -2.27217 | 0.02039 | 0.96565 |
| -2.55277 | -0.71511 | -0.72539 | 0.00381 | 1.03487 |
| -2.55277 | -0.71511 | -0.72539 | 0.00381 | 1.03487 |
| -0.94857 | -3.79376 | -3.80785 | 0.02697 | 0.85287 |
| -1.75067 | -2.25444 | -2.27217 | 0.02039 | 0.96565 |
| -2.55277 | 2.85079 | 2.89179 | 0.06056 | 0.94043 |
| -1.75067 | 1.31147 | 1.32179 | 0.0069 | 1.00666 |
| -2.55277 | 2.85079 | 2.89179 | 0.06056 | 0.94043 |
| -1.75067 | 1.31147 | 1.32179 | 0.0069 | 1.00666 |
| -2.55277 | 2.85079 | 2.89179 | 0.06056 | 0.94043 |
| -1.75067 | -2.25444 | -2.27217 | 0.02039 | 0.96565 |
| -1.75067 | -2.25444 | -2.27217 | 0.02039 | 0.96565 |
| -1.75067 | -2.25444 | -2.27217 | 0.02039 | 0.96565 |
| -1.75067 | -2.25444 | -2.27217 | 0.02039 | 0.96565 |
| -1.75067 | -2.25444 | -2.27217 | 0.02039 | 0.96565 |
| -0.94857 | -3.79376 | -3.80785 | 0.02697 | 0.85287 |
| -1.75067 | 1.31147 | 1.32179 | 0.0069 | 1.00666 |
| -0.94857 | -0.22786 | -0.22871 | 0.0001 | 1.01903 |
| -1.75067 | 1.31147 | 1.32179 | 0.0069 | 1.00666 |
| -0.94857 | -0.22786 | -0.22871 | 0.0001 | 1.01903 |
| -1.75067 | 1.31147 | 1.32179 | 0.0069 | 1.00666 |
| -1.75067 | 1.31147 | 1.32179 | 0.0069 | 1.00666 |
| -1.75067 | 1.31147 | 1.32179 | 0.0069 | 1.00666 |
| -1.75067 | 1.31147 | 1.32179 | 0.0069 | 1.00666 |
| -1.75067 | 1.31147 | 1.32179 | 0.0069 | 1.00666 |
| -0.94857 | -0.22786 | -0.22871 | 0.0001 | 1.01903 |
| -1.75067 | 1.31147 | 1.32179 | 0.0069 | 1.00666 |
| -0.94857 | -0.22786 | -0.22871 | 0.0001 | 1.01903 |
| -1.75067 | 1.31147 | 1.32179 | 0.0069 | 1.00666 |
| -0.94857 | -0.22786 | -0.22871 | 0.0001 | 1.01903 |
| -1.75067 | 1.31147 | 1.32179 | 0.0069 | 1.00666 |
| -0.94857 | -0.22786 | -0.22871 | 0.0001 | 1.01903 |
| -0.94857 | -0.22786 | -0.22871 | 0.0001 | 1.01903 |
| -1.75067 | 1.31147 | 1.32179 | 0.0069 | 1.00666 |
| -0.94857 | -0.22786 | -0.22871 | 0.0001 | 1.01903 |
| -1.75067 | 1.31147 | 1.32179 | 0.0069 | 1.00666 |
| -0.94857 | -0.22786 | -0.22871 | 0.0001 | 1.01903 |
| -1.75067 | 1.31147 | 1.32179 | 0.0069 | 1.00666 |
| -1.75067 | 1.31147 | 1.32179 | 0.0069 | 1.00666 |
| -1.75067 | -0.47149 | -0.47519 | 0.00089 | 1.02534 |
| -1.75067 | -0.47149 | -0.47519 | 0.00089 | 1.02534 |
| -1.75067 | -0.47149 | -0.47519 | 0.00089 | 1.02534 |
| -1.75067 | -0.47149 | -0.47519 | 0.00089 | 1.02534 |
| -1.75067 | -0.47149 | -0.47519 | 0.00089 | 1.02534 |
| -1.75067 | -0.47149 | -0.47519 | 0.00089 | 1.02534 |
| -1.75067 | -0.47149 | -0.47519 | 0.00089 | 1.02534 |
| -1.75067 | -0.47149 | -0.47519 | 0.00089 | 1.02534 |
| -1.75067 | -0.47149 | -0.47519 | 0.00089 | 1.02534 |
| -1.75067 | -0.47149 | -0.47519 | 0.00089 | 1.02534 |
| -0.94857 | -2.01081 | -2.01828 | 0.00758 | 0.97054 |
| -1.75067 | -0.47149 | -0.47519 | 0.00089 | 1.02534 |
| -1.75067 | -0.47149 | -0.47519 | 0.00089 | 1.02534 |
| -1.75067 | -0.47149 | -0.47519 | 0.00089 | 1.02534 |
| -1.75067 | -0.47149 | -0.47519 | 0.00089 | 1.02534 |
| -1.75067 | -0.47149 | -0.47519 | 0.00089 | 1.02534 |
| -0.94857 | -2.01081 | -2.01828 | 0.00758 | 0.97054 |
| -1.75067 | -0.47149 | -0.47519 | 0.00089 | 1.02534 |
| -0.94857 | -2.01081 | -2.01828 | 0.00758 | 0.97054 |
